# A smart and responsive crystalline porous organic cage membrane with switchable pore apertures for graded molecular sieving

**DOI:** 10.1038/s41563-021-01168-z

**Published:** 2022-01-10

**Authors:** Ai He, Zhiwei Jiang, Yue Wu, Hadeel Hussain, Jonathan Rawle, Michael E. Briggs, Marc A. Little, Andrew G. Livingston, Andrew I. Cooper

**Affiliations:** 1 Department of Chemistry and Materials Innovation Factory, University of Liverpool, Liverpool, UK; 2 Department of Chemical Engineering, Imperial College London, South Kensington, London, UK; 3 School of Engineering and Materials Science, Queen Mary University of London, London, UK; 4 Diamond Light Source, Didcot, UK; 5 Leverhulme Research Centre for Functional Materials Design, University of Liverpool, Liverpool, UK

**Keywords:** Materials science, Two-dimensional materials, Porous materials, Nanoscale materials, Self-assembly

## Abstract

Membranes with high selectivity offer an attractive route to molecular separations, where technologies such as distillation and chromatography are energy intensive. However, it remains challenging to fine tune the structure and porosity in membranes, particularly to separate molecules of similar size. Here, we report a process for producing composite membranes that comprise crystalline porous organic cage films fabricated by interfacial synthesis on a polyacrylonitrile support. These membranes exhibit ultrafast solvent permeance and high rejection of organic dyes with molecular weights over 600 g mol^−1^. The crystalline cage film is dynamic, and its pore aperture can be switched in methanol to generate larger pores that provide increased methanol permeance and higher molecular weight cut-offs (1,400 g mol^−1^). By varying the water/methanol ratio, the film can be switched between two phases that have different selectivities, such that a single, ‘smart’ crystalline membrane can perform graded molecular sieving. We exemplify this by separating three organic dyes in a single-stage, single-membrane process.

## Abstract (web summary)

The separation of multicomponent mixtures is performed by distillation, as multiple-membrane cascades are too complex. Here, a porous organic cage composite undergoes solid-state transformation in solvent; this alters pore size, enabling graded separation of three dyes with a single membrane.

## Main

Porous organic cages (POCs)^1,2^ are discrete molecules with intrinsic cavities that can create porosity in molecular crystals^1^, amorphous solids^3^ and porous liquids^4^. The adsorption properties of POCs can sometimes be predicted in silico from knowledge of their molecular structures in isolation^5,6^. However, the adsorption properties of POC materials are also affected by their solid-state packing^2,7^. For example, extrinsic pores in POC crystals can selectively adsorb guests, including rare gases^8^. Indeed, inefficient packing of POCs can generate solids with considerably more porosity than would be expected from the cage cavities alone^2,7^. This combination of intrinsic and extrinsic porosity determines the functionality of POC-based materials in selective adsorption processes.

Most separation studies involving POCs have used molecular crystals^2,7^, which can exhibit slow adsorption kinetics. Also, many POC crystals rely on selective adsorption governed by thermodynamics, rather than kinetics, which limits their practical use in size- and shape-selective membrane filters. Given their solution processability, however, there is scope to develop crystalline POC-based membranes that operate by selectively removing guests that are either too large or that have the wrong shape to diffuse through the POC pore structure.

There is growing interest in membrane technologies that perform industrial and environmentally relevant separations where two or more solutes are separated one from each other, as in distillation or chromatography, as opposed to separations where a whole set of solutes is concentrated, such as in evaporation or seawater reverse osmosis^9–13^. A major advantage of membranes is that they can perform separations in the liquid phase, which is often more practically useful than vapour phase separations.

Membranes for liquid separations are typically produced using phase inversion, which can be followed by coating^14^ or interfacial polymerization^15^. This produces amorphous polymer networks with a modest degree of pore tunability. There is a strong demand to develop membranes with more tunable and modular pore structures. Various porous solids, including zeolites^16^, POCs^1,2^, organic polymers^17^, metal–organic frameworks^18^, covalent organic frameworks (COFs)^19^ and hydrogen-bonded organic frameworks^20^ have been explored. Banerjee et al. reported COF films with 1.4 to 2.6 nm pores that showed good performance in dye rejection^21^. Dichtel et al. reported COF films with 3.4 nm pores and tunable thicknesses over the range of 100 μm to 2.5 nm that rejected Rhodamine WT from water^22^. The same team also reduced the effective pore size of their COF membrane to 3.3 and 3.2 nm using reticular chemistry^23^. In addition to COFs^24,25^, metal–organic frameworks and their composites have been used to produce membranes^24,26^. However, it remains challenging to produce continuous nanofiltration membranes with extended porous frameworks that perform exclusively as size-based molecular sieves rather than selective adsorbents^27^. POCs are solution processable and their solid-state structures are defined by non-covalent intermolecular interactions, which can be switched using chemical stimuli to alter their bulk porosity^28,29^. As such, POCs are intriguing but relatively unexplored candidates for new types of membrane materials^30–37^.

Many practically important molecular separations involve ternary systems or more complex mixtures—for example, separating multiple hydrocarbon fractions from light crude oil by distillation, pervaporation or organic solvent reverse osmosis^38,39^; purification of fatty acids^40,41^, such as the practical recovery of omega-3 polyunsaturated fatty acids from fish oil by nanofiltration^42^; or sieving out by-products from reactions, for example in the liquid-phase peptide synthesis of pharmaceuticals^43^. To achieve equivalent separations for complex mixtures using membranes, cascades of membranes with graded molecular weight cut-offs (MWCOs) have been developed^44^, using phase inversion (polymeric membranes)^45^ or sol–gel processing (ceramic membranes)^46^ by manipulating the recipe for dope solution or fabrication conditions to produce multiple membranes with a variety of pore sizes. This places membranes at a disadvantage for ternary and higher separations—by contrast, a single distillation or chromatography column can produce multiple fractions with differing compositions. Separating more than binary solute systems using a membrane cascade requires multiple pumped recycle streams and complex fluid controls^47^. While solvent gradients are used in chromatography to modulate solid–liquid interactions, to the best of our knowledge, there are as yet no reports of membranes that respond to solvent gradients by changing their solute selectivity.

**Fig. 1:**
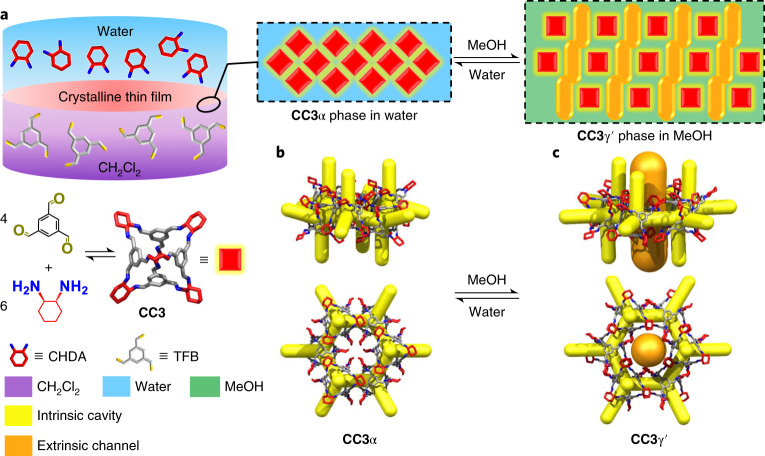
Synthesis of a crystalline CC3 film and its crystal structures. **a**, Scheme showing the interfacial synthesis method used to fabricate crystalline **CC3** films, which were subsequently adhered to a PAN support. These crystalline cage films can cycle between two different forms, **CC3**α-PAN and **CC3**γ′-PAN, by cycling the solvent between water and MeOH. CH_2_Cl_2_, dichloromethane. **b**, **CC3**α structure with its 3D pore network shown in yellow. **c**, The **CC3**γ′ structure, formed by soaking in MeOH, has additional extrinsic solvent-filled channels, shown here in orange, that open up additional porosity in the membrane in response to the MeOH solvent.

Here, we report the fabrication of close-packed and defect-free films of a shape-persistent imine POC, **CC3**, which grow at the liquid–liquid interface between water and dichloromethane (Fig. 1a). These films comprise highly crystalline domains of **CC3** in its most thermodynamically stable polymorph, **CC3**α (Fig. 1b). By coating the **CC3**α film on polyacrylonitrile (PAN), we produce a continuous membrane (**CC3**α-PAN) that has excellent permeance for both polar and non-polar solvents, including water (43.0 l m^−2^ h^−1^ bar^−1^) and toluene (55.9 l m^−2^ h^−1^ bar^−1^). Furthermore, we found that it is possible to rapidly and reversibly switch the membrane pore aperture using common solvents. Exposure of the non-covalent crystal packing of **CC3** in methanol (MeOH) induces a rapid phase transition from **CC3**α to a different crystalline phase, **CC3**γ′, which is less densely packed. This systematically increases the effective pore aperture of the resulting membrane, **CC3**γ′-PAN (Fig. 1c). This switching property of the **CC3**γ′﻿-PAN membrane allows the permeation of larger organic dyes that can be rejected in water while the large pore apertures are turned ‘off’ in the **CC3**α-PAN membrane. This switchable porosity is reversible, and surprisingly, it does not compromise the continuity of the membrane. This allowed us to separate three organic dyes with different sizes via graded sieving using a single membrane.

## Fabrication of crystalline CC3 films

**Fig. 2:**
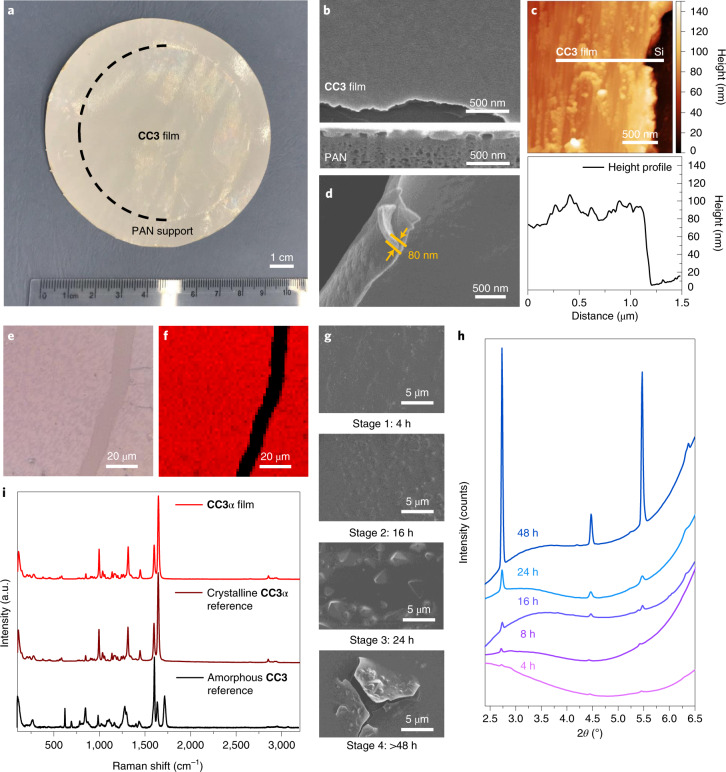
Characterization of a CC3α film. **a**, Photograph of composite membrane **CC3**α-PAN with a diameter of 7.4 cm. **b**, SEM image of **CC3**α-PAN showing the surface morphology of the **CC3**α film. Shown below is the cross-sectional FIB-SEM image of **CC3**α-PAN. **c**, AFM height image (top) and the height profile (bottom) of **CC3**α film transferred onto a silicon (Si) wafer. **d**, SEM image of a free-standing **CC3**α film, where the film was deliberately buckled to show its thickness. **e**,**f**, Raman microscope image (**e**) and Raman map (**f**) of a **CC3**α film on a glass support, where we purposely scratched the film before the measurement to expose the glass support (black stripe in **f**). The red regions on a **CC3**α film had comparable Raman spectra to the crystalline **CC3**α reference sample. **g**, SEM images of **CC3**α-PAN-*X* h-0.8% membranes formed at different reaction times, showing four stages of **CC3**α film formation. **h**, Out-of-plane GIXRD (wavelength, *λ* = 0.689 Å) patterns of **CC3**α-PAN-*X* h-0.8% membranes fabricated using reaction times between 4 and 48 hours (2*θ* refers to the scattering angle). **i**, Raman spectra of **CC3**α film, a crystalline **CC3**α reference^1^ and an amorphous **CC3** reference^3^. Source data

Continuous films with highly crystalline domains of **CC3** were produced using a combined interfacial condensation reaction and crystallization process at a water–dichloromethane interface (Fig. 1a). This interfacial process allows the two-component reaction of **CC3**, which is synthesized via a [4 + 6] cycloimination reaction using 1,3,5-triformylbenzene (TFB) and (1*R*,2*R*)-1,2-diaminecyclohexane (CHDA), while simultaneously directing the formation of **CC3** films at the interface (Methods). Continuous and free-standing **CC3** films were transferred from the liquid–liquid interface onto various substrates (for example, glass, steel mesh, carbon tape and silicon wafers; Supplementary Fig. 1) for further analysis of the crystallinity and surface morphology. Before performing permeance and dye rejection studies, the **CC3** film was coated onto a PAN support by filtration to form the composite membrane (Fig. 2a). The resulting membrane, referred to hereafter as **CC3**-PAN, was free of macroscopic defects up to at least 7.4 cm in diameter using this preparation process, with no evidence of delamination after cutting the membrane into smaller pieces (Supplementary Fig. 2). The **CC3** film was characterized by Fourier transform infrared spectroscopy (Supplementary Fig. 3), Raman spectroscopy, nuclear magnetic resonance (NMR) spectroscopy (Supplementary Fig. 4), scanning electron microscopy (SEM), focused ion beam SEM (FIB-SEM), X-ray diffraction and atomic force microscopy (AFM). For spectroscopic measurements, a crystalline **CC3**α sample was used as a reference^1^. **CC3**α has a three-dimensional (3D) diamondoid pore structure and is the thermodynamically most stable polymorph **CC3** (ref. ^1^). A Raman map was performed on an 80 × 80 μm^2^ **CC3** film deposited on glass (Fig. 2e,f and Supplementary Fig. 5), which indicated that the **CC3** film comprised crystalline domains with the same solid-state structure as the **CC3**α polymorph (Fig. 2i). SEM images showed a continuous, apparently defect-free film in the **CC3**-PAN composite (Fig. 2b and Supplementary Fig. 6a) with a thickness of ~80 nm measured on a free-standing film (Fig. 2d), which contained embedded, octahedral **CC3** crystals (Supplementary Fig. 7). Cross-sectional SEM images were obtained after step-by-step FIB trenching and polishing of both **CC3**-PAN (Supplementary Fig. 8) and a **CC3** film coated on a silicon wafer (Supplementary Fig. 9). The FIB-SEM images showed a clear boundary between the **CC3** film layered on top of the supports. After transferring the as-synthesized **CC3** film onto a silicon wafer, we performed AFM measurements to investigate the film thickness further. AFM again confirmed that the **CC3** film was continuous with a constant thickness of ~80 nm (Fig. 2c and Supplementary Figs. 6b, 8 and 9).

A key advantage of interfacial synthesis is that it can create continuous films of the product^15,21^. Here, we also modified the reaction conditions to optimize the thickness, continuity and crystallinity of the **CC3** film (Fig. 2g,h). This allowed us to create **CC3** films from the interfacial reaction that were four times thinner than the **CC3** film created by spin coating^40^. To further confirm the crystalline structure of the film, we performed a series of powder X-ray diffraction (PXRD) and grazing incidence X-ray diffraction (GIXRD) measurements on **CC3**-PAN (Methods). These diffraction measurements revealed that the **CC3** film was crystalline and had the same structure as **CC3**α (Supplementary Figs. 10 and 11).

To further investigate the crystallization process of **CC3** films at the solvent interface, we varied the reaction time from 4 to 96 hours and manipulated the reagent concentrations from 0.2 to 2.5 wt%. We use the nomenclature **CC3**α-PAN-*X* h-*Y*% to refer to the membranes made with *X* hours of reaction time and *Y* weight percent of the reagents. SEM, FIB-SEM and AFM revealed that thicker films with larger crystals were produced as the reaction time and reagent concentrations were increased (Supplementary Figs. 12–21). By contrast, using a reagent concentration of 0.2 wt% resulted in poorly crystalline **CC3** membranes (Supplementary Figs. 14 and 15). For the reactions with reagent concentrations of 0.8 wt%, the **CC3** film thickness increased with reaction time (30–600 nm from the 4–60 h reactions; Supplementary Figs. 16 and 17), and the FIB-SEM images revealed triangle/octahedral-shaped crystals embedded in the **CC3** films from the 32 and 48 h reactions (Supplementary Figs. 18–20). By contrast, from the reactions with reagent concentrations of 2.0%, multiple **CC3** films were found stacked on top of one another (Supplementary Fig. 21). We suggest that the interfacial synthesis occurs in four stages (Fig. 2g): Stage 1 (0–4 hours), interfacial polymerization of a continuous oligomeric film at the dichloromethane–water interface; Stage 2 (4–16 hours), self-sorting of the reactants and oligomers into the **CC3**α product and the formation of a partially reacted, semi-cage film; Stage 3 (24–48 hours), crystallization of **CC3**α and the formation of octahedral crystals in the film; and Stage 4 (48–96 hours), formation of defects in the film caused by larger octahedral crystals creating cracks and imperfections. GIXRD measurements demonstrated the crystallization process across these stages, where the crystallinity increased with a longer reaction time (Fig. 2h). We therefore focused attention on the properties of **CC3**α-PAN-24 h-0.8%, referred to hereafter as **CC3**α-PAN.

## Membrane performance of CC3α-PAN

To determine the permeance and dye rejection performance of **CC3**α-PAN, we performed filtration experiments in dead-end cells using solvents and dyes with different sizes and chemical functionalities (Supplementary Table 1 and Supplementary Figs. 22 and 23).

**Fig. 3:**
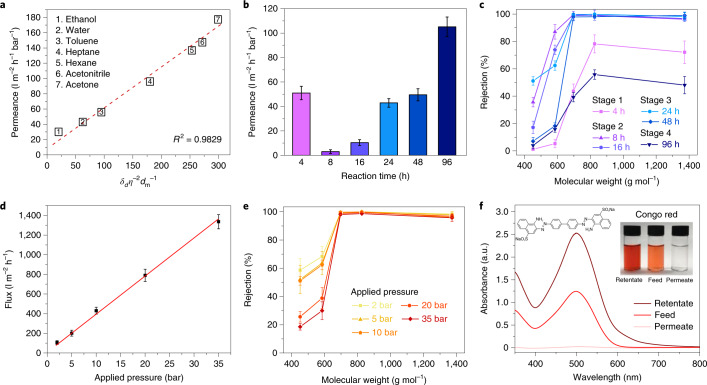
Nanofiltration performance of CC3α membranes. **a**, Plot showing pure solvent permeances versus their combined solvent properties (viscosity *η*, molar diameter *d*_m_ and solubility parameter *δ*_*d*_) for **CC3**α-PAN, where *R*^2^ is the coefficient of determination for the function. Hansen solubility parameter (*δ*) and the physical properties of each organic solvent are listed in Supplementary Table 2. **b**, Water permeance for **CC3**α-PAN-*X* h-0.8% membranes fabricated using reaction times that ranged between 4 and 96 hours. **c**, Dye rejection measurements for **CC3**α-PAN-*X* h-0.8% membranes in water. **d**,**e**, Water flux (**d**) and dye rejections (**e**) of a **CC3**α-PAN membrane under a range of applied pressures. **f**, Ultraviolet–visible absorption spectra of Congo red in water before (feed) and after (permeate and retentate) selectivity tests performed with **CC3**α-PAN. Insets show photographs of the feed, permeate and retentate solutions and the molecular structure of Congo red. Dye rejection was calculated using the intensity of the maximum absorption peak in the permeate and the feed, and equation (3) in the Methods. Mass balance calculations were performed using the maximum absorption peak values of the feed, permeate and retentate, with equation (4). All error bars depict the standard deviation (s.d.) of the data points obtained from at least three independent membranes. Source data

With a water contact angle of 94° (Supplementary Fig. 24), the **CC3**α-PAN membrane was stable in a range of polar and non-polar solvents (Supplementary Fig. 25), proving that these solvents do not dissolve **CC3**. This led to ultrafast solvent permeances (Fig. 3a; Supplementary Fig. 26 for blank PAN data). We attribute this to the 3D interconnected porosity through the **CC3**α crystals in the film. By comparison, an amorphous **CC3** membrane prepared by spin coating (Supplementary Section 1.2) exhibited a 30-fold lower solvent permeance under the same testing conditions (Supplementary Fig. 27), although it should also be noted that the amorphous **CC3** membrane was four times thicker^30^. Apparently, the crystalline **CC3**α-PAN provides sufficient robustness to support the interconnected channels under high applied pressures. To further confirm the importance of crystallinity, **CC3**α membranes with different crystallinity levels were fabricated at each of the four reaction stages simply by controlling the reaction time. A partially crystalline membrane (**CC3**α-PAN-8 h-0.8%) at Stage 2 exhibited a water permeance of 3.0 l m^−2^ h^−1^ bar^−1^; that is, an order of magnitude lower than the fully crystalline Stage 3 membrane (49.5 l m^−2^ h^−1^ bar^−1^ for **CC3**α-PAN-48 h-0.8%) prepared with prolonged reaction times (Fig. 3b). **CC3**α-PAN-8 h-0.8% and **CC3**α-PAN-48 h-0.8% exhibited the same MWCO, as determined by filtering a range of dyes through the membranes (Fig. 3c). By comparison, amorphous oligomeric membranes produced in Stage 1 (**CC3**α-PAN-4 h-0.8%) and cracked, highly crystalline membranes produced in Stage 4 (**CC3**α-PAN-96 h-0.8%) exhibited unexpectedly higher water permeances but failed to achieve comparable separation performances, indicating that they contained physical defects.

Two limitations of membranes produced from other crystalline porous materials, such as COFs, are poor stability at high pressures^21^ and the interference of adsorption processes^27^. Here, the **CC3**α-PAN membrane was tested under a range of applied pressures, up to a maximum of 35 bar. The water flux increased linearly with increasing applied pressure (Fig. 3d) without affecting the MWCO (Fig. 3e). Longer duration studies demonstrated the mechanical robustness of **CC3**α-PAN and showed consistent dye rejection (99.7% for rose bengal) and water permeance (~43 l m^−2^ h^−1^ bar^−1^) over 20 hours (Supplementary Fig. 28). The applied pressure of 35 bar is an order of magnitude higher than that used for liquid filtration through COF membranes^21^, which suggests that these **CC3**α-PAN membranes might be more competitive for separations that require higher pressures.

To confirm that dye adsorption did not contribute to the selectivity performance of **CC3**α-PAN, mass balance calculations were used to measure the dye concentration in the retentate. After permeating 48 ml of Congo red from the 100 ml feed, the absorption intensity of Congo red in the retentate increased from 1.24 to 2.53, while its absorption intensity in the permeate was only 0.02. In combination, these values are consistent with ~100% dye rejection (Fig. 3f and Supplementary Figs. 29 and 30). These measurements agree with the colourless membrane surface observed after the dye filtration (Supplementary Fig. 2). In addition, soaking powdered crystals of **CC3**α (100 mg) in the dye solution (100 ml) did not lead to adsorption in the crystals after seven days (Supplementary Fig. 31). These results all indicate that the dyes were rejected by the membrane.

## Switchable pore aperture for graded sieving

Previous studies showed that certain POCs can be switched between multiple polymorphs to modify their porosity^28,29^. The solid-state structure of **CC3** has been directed into different polymorphs by crystallization from specific solvents^48^, but until now, the solid-state transformation of **CC3** crystals was not explored, to the best of our knowledge.

**Fig. 4:**
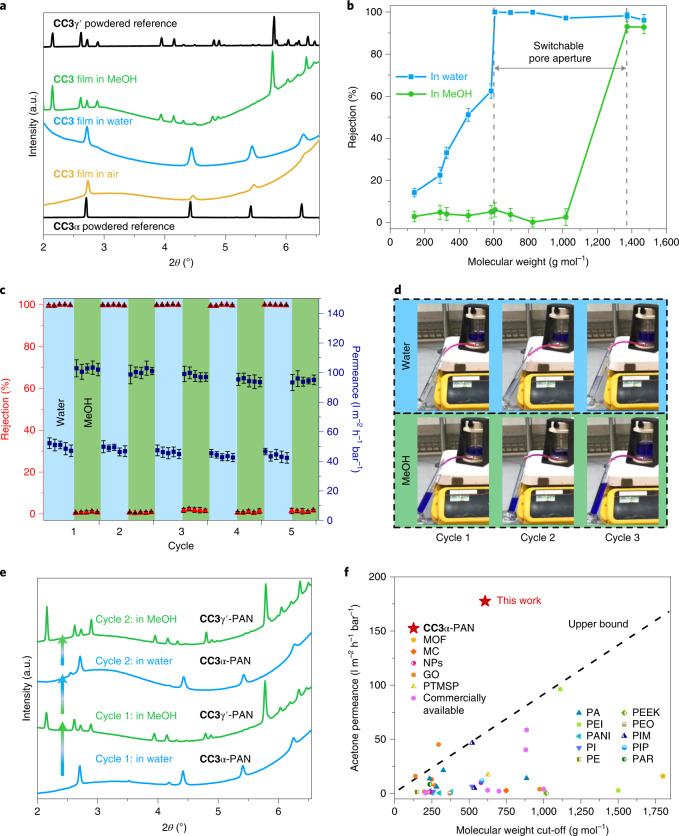
X-ray diffraction characterization and switchable separation performance of CC3-PAN membranes. **a**, GIXRD of **CC3**α-PAN in air and water, and **CC3**γ′-PAN in MeOH. Experimental PXRD patterns of **CC3**α and **CC3**γ′ powders are included as references. **b**, MWCO curve for **CC3**α-PAN in water and **CC3**γ′-PAN in MeOH containing 20 ppm dye solutes. The MWCO was determined by interpolating from the plot of rejection against the molecular weight of the dyes and corresponds to the molecular weight for which rejection reaches 90%. All error bars depict the s.d. of the data points obtained from at least three independent membranes. **c**, Reversible dye rejection of BB and solvent permeance of the **CC3**-PAN membrane observed upon switching the feedstock solvent between water and MeOH. All error bars denote the s.d. for measurements from at least three independent membranes. **d**, Photographs of **CC3**-PAN filtration dead-end cell captured from Supplementary Video 1 during the different cycles; BB is rejected in water by **CC3**α-PAN while **CC3**γ′-PAN does not reject BB in MeOH. **e**, In situ GIXRD patterns showing the reversible phase transition between **CC3**α-PAN and **CC3**γ′-PAN, by cycling between water and MeOH. **f**, Acetone permeance versus MWCO of general solutes in acetone for nanofiltration membranes reported in the literature and **CC3**α-PAN. MOF, metal–organic framework; MC, macrocycle; NPs, nanoparticles; GO, graphene oxide; PTMSP, poly(1-(trimethylsilyl)-1-propyne); PA, polyamide; PEI, polyethyleneimine; PANI, polyaniline; PI, polyimide; PE, polyethylene; PEEK, poly(ether ether ketone); PEO, poly(ethylene oxide); PIM, polymers of intrinsic microporosity; PIP, piperazine; PAR, polyacrylate (Supplementary Table 9 for full details). Source data

We found that both air-dried and water-solvated films exhibited the same diffraction patterns as the reference peaks of **CC3**α powders measured by PXRD (Fig. 4a). A series of GIXRD patterns were then recorded after submerging the membrane in various organic solvents (Supplementary Figs. 32 and 33). The crystalline **CC3**α film transformed into a new structure when submerged in MeOH (Fig. 4a). By indexing the GIXRD pattern, we confirmed this was a MeOH-solvated **CC3** phase (**CC3**γ′; Supplementary Fig. 34) that was isolated previously by crystallizing **CC3** from dichloromethane and MeOH (ref. ^49^). The **CC3**γ′ structure is very different from its thermodynamically most stable polymorph, **CC3**α (ref. ^50^), where the cage packs in a window-to-window arrangement to generate a diamondoid pore network (yellow channels in Fig. 1b). By contrast, the **CC3** molecules in the **CC3**γ′ phase are packed less densely, thus providing large extrinsic pores between hexagonally arranged **CC3** molecules (orange channels in Fig. 1c).

To investigate the structural transformation between **CC3**α-PAN and **CC3**γ′-PAN, we performed a series of in situ GIXRD measurements while dosing the membrane surface with solvent vapour and after coating the membrane surface in a thin solvent layer (Methods). **CC3**γ′-PAN formed by immersion in MeOH transformed back into **CC3**α-PAN after being immersed in water (Fig. 4e), with evidence of both phases found when the membrane was immersed in a mixture of water and MeOH (Supplementary Figs. 35 and 36). High-resolution PXRD also confirmed that **CC3**γ′ cleanly transforms into **CC3**α after thermally desolvating a powdered sample of **CC3**γ′ suspended in MeOH in a capillary (Supplementary Fig. 37).

We next used MeOH rather than water to dissolve the dyes and filtered these solutions through the **CC3**-PAN membrane under the same conditions. Interestingly, the MWCO shifted from 600 g mol^−1^ in water to 1,400 g mol^−1^ in MeOH for the same membrane (Fig. 4b and Supplementary Figs. 38 and 39). By contrast, a commercial Synder NDX nanofiltration membrane with a comparable MWCO (500–700 g mol^−1^) exhibited similar rejection behaviour in both water and MeOH (Supplementary Figs. 40 and 41). We attribute this dramatic change in MWCO to the phase transformation to **CC3**γ′-PAN in MeOH. We further investigated how crystallinity influences the switchable pore aperture by measuring dye rejection of **CC3**-PAN membranes with lower crystallinity (fabricated using lower concentrations or shorter reaction times; Supplementary Figs. 42–45). **CC3**-PAN-4 h-0.8% rejected 78.2% of brilliant blue (BB) dye from water compared to 52.7% from MeOH, while the less crystalline **CC3**-PAN-4 h-0.2% had a less distinct BB rejection performance (68.6% from water versus 52.8% from MeOH). Hence, the high crystallinity in the **CC3** membrane is essential for regulating its separation performance after switching its pore aperture using a solvent stimulus.

We next performed molecular separations while cycling between **CC3**α-PAN and **CC3**γ′-PAN using a single membrane and water and MeOH feedstocks containing the BB dye. We found that both water and MeOH permeances remained high after cycling between **CC3**α-PAN and **CC3**γ′-PAN (Fig. 4c, Supplementary Tables 5–7 and Supplementary Video 1). More importantly, the rejection of BB switches between ~100% in water to ~0% in MeOH in each cycle (Fig. 4c,d); that is, the membrane can be switched ‘on’ and ‘off’ using a solvent. The reversible transition between **CC3**α-PAN and **CC3**γ′-PAN appears to be complete within the one minute it takes to switch the feedstock (Supplementary Video 1) and creates alternative diffusion pathways through the membrane structure. From in situ GIXRD measurements on solvated **CC3** films, while performing two consecutive cycles, we found that the composite membrane transformed cleanly between **CC3**α-PAN and **CC3**γ′-PAN when the solvent was switched between water and MeOH and back again (Fig. 4e). We attributed this switching phenomenon solely to the phase transition of **CC3** films, rather than swelling of the membranes. To validate this, the **CC3**-PAN membrane was soaked in acetone and acetonitrile, and the nanofiltration tests were repeated. **CC3**-PAN exhibits comparable MWCOs in acetone and acetonitrile to those observed in water (Supplementary Figs. 41 and 46) because the same phase, **CC3**α-PAN, is present in these solvents (Supplementary Figs. 32 and 33). Remarkably, the acetone permeance of **CC3**α-PAN reached 177 l m^−2^ h^−1^ bar^−1^ with a MWCO of ~600 g mol^−1^, which is well above the upper bound performance for nanofiltration membranes reported in the literature (Fig. 4f, Supplementary Fig. 47 and Supplementary Table 9).

**Fig. 5:**
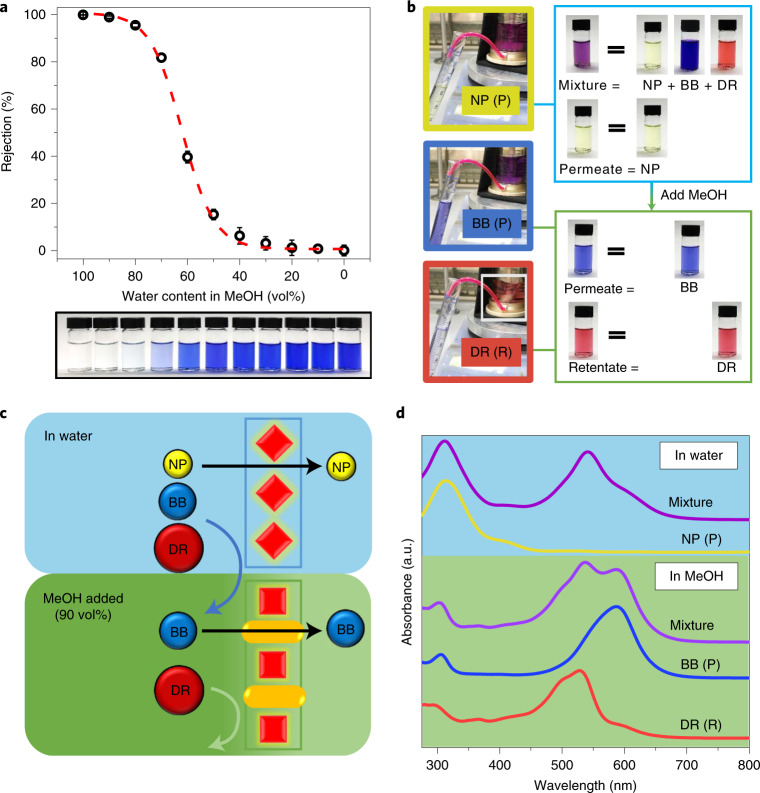
Mixture fitting and graded sieving using a single switchable membrane. **a**, BB rejection in mixtures of water and MeOH (v/v) for **CC3**-PAN (top), and photographs of the permeates (bottom). All error bars depict the s.d. of the data points obtained from at least three independent membranes. The red dashed line was fitted as the logistic function (*y* = 1/(1 + exp(−16.1(*x* – 0.617))); Supplementary Section 1.4). **b**, Photographs showing the ternary molecular separation in a filtration dead-end cell, the nascent mixture feedstock, the permeate (P) collected in the first and second step, and the retentate (R) collected in the second step. **c**, Scheme showing ternary molecular separation of three dyes (DR, BB and NP) using one single membrane (**CC3**-PAN) in a continuous process: Step 1, **CC3**α-PAN in water (blue background) allows permeation of only NP, leaving BB and DR in the retentate. Step 2, 90 vol% MeOH (green background) was added into the retentate to transform the membrane structure to **CC3**γ′-PAN, which allows permeation of only BB, leaving DR in the retentate. **d**, Ultraviolet–visible absorption spectra of the mixture containing three molecules in water, permeate from water, mixture and permeate from 90 vol% of MeOH in water and the remaining retentate. Note, the maximum absorbance wavelength for BB is 551 nm in water and 587 nm in MeOH; BB also shows absorbance at 305 nm in MeOH, while NP shows its maximum absorbance at 312 nm in the same solvent. Source data

A series of water and MeOH feedstocks containing the dye BB were used to determine the dynamic transformation between **CC3**α-PAN and **CC3**γ′-PAN (Fig. 5a). Understanding this dynamic transformation allowed us to manipulate the pore aperture in a single **CC3**-PAN membrane by simply adjusting the water concentration in a water–MeOH mixture, without any activation processes^51^ or the use of multiple membranes^21^. To demonstrate this, we performed a graded sieving experiment to separate molecules from a ternary mixture using a single membrane. Initially, a water feedstock containing three dyes, 4-nitrophenol (NP; yellow, 139 g mol^−1^), BB (blue, 826 g mol^−1^) and direct red 80 (DR; red, 1,373 g mol^−1^) was filtered through the **CC3**-PAN membrane (Fig. 5c). Since **CC3**-PAN adopts its **CC3**α-PAN structure in water, the narrower pore aperture allowed only the smallest molecule, NP, to diffuse through the membrane, while the larger molecules, BB and DR, were rejected. Excess water was used for flushing residual NP from the retentate, and this process was repeated until the NP concentration in the permeate was below 1%. Subsequently, 90 vol% of MeOH was added into the water retentate to generate a feedstock that transformed the membrane structure to **CC3**γ′-PAN with the larger pore aperture. BB could then diffuse through the membrane alone, while DR was retained in the cell (Fig. 5b,d). Finally, excess MeOH was used to flush any residual BB from the cell to leave only DR in the retentate, where it could be collected in pure form (Supplementary Section 1.4).

## Conclusion and outlook

Continuous, defect-free POC membranes can achieve high permeances for a range of organic solvents—in some cases exceeding upper performance bounds—while also showing excellent separation performances. These highly ordered crystalline POC membranes exhibit a switchable phase transition between two crystalline forms, **CC3**α-PAN and **CC3**γ′-PAN. This allows graded sieving to separate a mixture of three organic dyes using a single, smart membrane and creates a membrane-based parallel to the widespread and highly effective use of solvent gradients in chromatography^52^. POC membranes with switchable pore apertures could also lead to new applications in triggered drug delivery^53^, biosensors^54^ or fermentation/fractionation processes^55^.

While the current synthesis process makes it challenging to scale and implement these POC membranes in commercial processes, it is conceivable that a more scalable production method might be developed by exploiting the solution processability of these molecular cages. Future efforts will focus on using computational methods, such as crystal structure prediction, to design POC crystals with specific properties that can be designed from first principles.

## Methods

### Interfacial synthesis of crystalline CC3 films

An aqueous solution of CHDA (0.26 g, 2.24 mmol, 0.8 wt%) in water (32 ml) was carefully layered on top of a dichloromethane solution (30 ml) that contained TFB (0.24 g, 1.48 mmol, 0.8 wt%) and was stored in a glass dish with an inner diameter of 7.4 cm (Fig. 1a). The interfacial reaction was covered and kept at room temperature (~19–21 °C) for between 4 and 96 hours (typically, 24 hours). The continuous crystalline **CC3** film that grew at the dichloromethane–water interface was then isolated as a free-standing film that could be layered directly onto different substrates, including glass, steel mesh, carbon tape and silicon wafers. To perform liquid permeation studies, the **CC3** film was transferred onto a PAN support to form composite **CC3**-PAN membranes, which were then soaked in pure water for 1 day (Supplementary Figs. 48 and 49; Supplementary Section 1.2 for full experimental details and Supplementary Fig. 50 for the reaction set-up). Fabrication of PAN supports via phase inversion is presented in Supplementary Section 1.2.

### X-ray diffraction

GIXRD measurements were performed using the I07 beamline at Diamond Light Source in the United Kingdom (wavelength, *λ* = 0.689 Å), using a vertical (2 + 2)-type diffractometer equipped with a Pilatus 100 K area detector^56^. Membrane samples were cut into 1 × 2 cm^2^ pieces and stuck onto glass supports, which were then mounted on a hexapod (PI-Micos) to allow independent alignment with six degrees of freedom during the data collection (Supplementary Fig. 51a). The measurements were conducted by moving the detector while maintaining a fixed sample position. The grazing incidence angle is set at 2°. Data collection was performed at room temperature using in-plane (over the 2*θ* range 3–40°, 0.50° step size) and out-of-plane (over the 2*θ* range 2–40°, 0.25° step size) measurement geometries, and GIXRD scans were processed in DAWN 2 (ref. ^57^). GIXRD patterns were refined by Pawley refinement through TOPAS Academic^58^. High-resolution synchrotron PXRD data were collected using the I11 beamline at Diamond Light Source (*λ* = 0.827 Å). The full PXRD details are presented in the Supplementary Information.

For the in situ GIXRD measurements performed on solvated samples, pieces of Mylar film were used to cover the membrane surface with a thin layer of solvent (water, MeOH, acetone and acetonitrile) during the GIXRD scans (Supplementary Fig. 51b). To investigate the reversible transformation between **CC3**α-PAN and **CC3**γ′-PAN, a membrane sample was removed from water without drying and covered with 1.0 ml of a MeOH solvent layer before recording the GIXRD data (Supplementary Fig. 35). To more closely mimic the reversible membrane separation experiment where the feedstock was cycled between water and MeOH, a **CC3**-PAN sample was removed from water without drying, soaked in 100 ml MeOH for 1 minute and covered with a thin layer of MeOH (1.0 ml) before the GIXRD measurement. The same process was repeated with the identical **CC3**-PAN sample using water or MeOH (Fig. 4e). For the in situ measurements performed using solvent vapours, nitrogen gas was bubbled through a 2 l bottle that contained the organic solvent at a flow rate of 10 l min^−1^. The ‘wet gas’ generated during this process was then continually flowed over the membrane sample during the full measurement and Mylar film was used to seal the sample environment.

### Separation measurements

Solvent permeance and dye rejection measurements were performed using a Sterlitech HP4750 dead-end membrane filtration system (Supplementary Fig. 22). We also used a commercial bench-scale 50 ml transparent Merck Millipore Amicon dead-end stirred cell, which was connected to an 800 ml Merck Millipore Amicon RC800 reservoir, to visualize the filtration process (Supplementary Fig. 23). During these measurements, the feedstocks were kept under a 10 bar nitrogen pressure (3 bar for Merck Millipore Amicon cells) at room temperature, and the feedstock was continually stirred using a stirring bar rotating at 400 r.p.m. The Hansen solubility parameter (*δ*) and the physical properties of the organic solvents (Supplementary Table 2) were used to investigate the relationships between pure solvent permeances and the combined solvent properties.

Flux *J* (l m^−2^ h^−1^) was calculated according to the following equation:

1 $$J = {\Delta}V/\left( {A \times {\Delta}t} \right)$$

where ∆*V* is the volume of permeate collected in litres in a given amount of time, *A* is the membrane surface area in square metres and ∆*t* is the time in hours between the start and end of the measurement.

Solvent permeance *P* (l m^−2^ h^−1^ bar^−1^) was calculated according to the following equation:

2 $$P = {\Delta}V/\left( {A \times {\Delta}t \times p} \right)$$

where ∆*V* is the volume of permeate collected in litres in a given amount of time, *A* is the membrane surface area in square metres, ∆*t* is the time in hours between the start and end of the measurement and *p* is the transmembrane pressure. To calculate solvent permeance, typically, 0.2 l of pure solvent or dye feedstock (20 ppm dye concentration) was added to the feedstock tank. The cell was then pressurized to 10 bar under nitrogen. The solvent permeate was then calculated based on the amount of time it took ~0.1 l of pure solvent or dye feedstock to flow through the membrane (Supplementary Tables 3 and 4 for full details). The retentate was collected after each measurement. Error bars (s.d.) were calculated by the STDEV.P function using data obtained from at least three independent membranes.

For the dye rejection measurements, a series of dye feedstock solutions in different solvents (water, MeOH, acetone and acetonitrile) were prepared with a dye concentration of 20 ppm using the following dyes: reactive red 120 (1,470 g mol^−1^), DR (1,373 g mol^−1^), rose bengal (1,018 g mol^−1^), BB (826 g mol^−1^), Congo red (697 g mol^−1^), protoporphyrin IX disodium (607 g mol^−1^), acid fuchsin (585 g mol^−1^), sunset yellow (452 g mol^−1^), methyl orange (327 g mol^−1^), neutral red (289 g mol^−1^) and NP (139 g mol^−1^; Supplementary Table 1 for full details). Ultraviolet–visible spectroscopy was used to measure the dye concentration in the permeate to calculate dye rejection performance. Dye rejection, *R* (%), of the membranes was calculated as follows:

3 $$R\% = \left( {1 - C_{\mathrm{p}}/C_{\mathrm{f}}} \right) \times 100$$

where *C*_p_ and *C*_f_ represent the dye concentrations in the permeate (*C*_p_) and feed (*C*_f_). Dye concentrations in the permeate and feed were determined using a Cary 5000 ultraviolet/visible/near-infrared spectrometer with the wavelengths specified in Supplementary Table 1. The MWCO was determined by interpolating from the plot of rejection against the molecular weight of the dyes and corresponds to the molecular weight for which rejection is 90%. During these measurements, the volume and the concentration of the permeate and the retentate were measured, and the mass balance of the feed solution could be calculated as follows:

4 $$C_{\mathrm{f}} \times V_{\mathrm{f}} = C_{\mathrm{p}} \times V_{\mathrm{p}} + C_{\mathrm{r}} \times V_{\mathrm{r}}$$

where *C*_f_, *C*_p_ and *C*_r_ are the dye concentrations in parts per million (grams per litre) of the feed, permeate and retentate, respectively; *V*_f_, *V*_p_ and *V*_r_ represent the volume of the feed, permeate and retentate in litres, respectively. Typically, 0.2 l of the feed solution was added into the cell, then 0.1 l permeate was collected and 0.1 l retentate was left in the cell. Reversible filtration tests, membrane absorption tests, long-term operation information, membrane stability tests, water and MeOH feedstock mixture separation experiments and graded sieving experiments for the ternary system are presented in Supplementary Section 1.4. The set-up of a commercial bench-scale dead-end stirred filtration unit with transparent cells (a 50 ml transparent Merck Millipore Amicon dead-end stirred cell connected to an 800 ml Merck Millipore Amicon RC800 reservoir) is shown in Supplementary Fig. 23. Reversible filtration measurement data in water and MeOH are shown in Supplementary Tables 5–7, and dye rejection measurement data in a water and MeOH mixture are shown in Supplementary Table 8.

## Online content

Any methods, additional references, Nature Research reporting summaries, source data, extended data, supplementary information, acknowledgements, peer review information; details of author contributions and competing interests; and statements of data and code availability are available at 10.1038/s41563-021-01168-z.

## Supplementary information

Supplementary Information Supplementary Figs. 1–51, Tables 1–9, Discussion, Materials and Methods, and refs. 1–47.

Supplementary Video 1 Video showing the switchable membrane selectivity at ×40 actual speed.

Supplementary Data 1 Statistical source data used for the Supplementary Information figures 3, 4, 10, 11, 32–34, 37, 42–44 and 46.

## Data Availability

Source data are provided with this paper. In addition, source data are deposited in the University of Liverpool Research Data Catalogue (10.17638/datacat.liverpool.ac.uk/1512). Further details can be obtained from the authors upon request.

## Source data

Source Data Fig. 2 Statistical source data for Fig. 2.
Source Data Fig. 3 Statistical source data for Fig. 3.
Source Data Fig. 4 Statistical source data for Fig. 4.
Source Data Fig. 5 Statistical source data for Fig. 5.
