# Supplementary material for: A smart and responsive crystalline porous organic cage membrane with switchable pore apertures for graded molecular sieving
Source: Nat Mater. 2022 Jan 10;21(4):463–70. doi: 10.1038/s41563-021-01168-z (PMC8971131; doi:10.1038/s41563-021-01168-z)
Supplement: Supplementary file 1 — Supplementary Figs. 1–51, Tables 1–9, Discussion, Materials and Methods, and refs. 1–47. [file 41563_2021_1168_MOESM1_ESM.pdf]

nature portfolio

https://doi.org/10.1038/s41563-021-01168-z

Supplementary information

A smart and responsive crystalline porous organic cage membrane with switchable pore apertures for graded molecular sieving

In the format provided by the authors and unedited

Supplementary Information for

A Smart and Responsive Crystalline Porous Organic Cage Membrane with Switchable Pore Apertures for Graded Molecular Sieving

Ai He[1], Zhiwei Jiang[2,3], Yue Wu[1], Hadeel Hussain[4], Jonathan Rawle[4], Michael E. Briggs[1], Marc A. Little[1], Andrew G. Livingston[2,3*], Andrew I. Cooper[1,5*]

[1]Department of Chemistry and Materials Innovation Factory, University of Liverpool, 51 Oxford Street, Liverpool, UK.

[2]Department of Chemical Engineering, Imperial College London, South Kensington, London, UK.

[3]School of Engineering and Materials Science, Queen Mary University of London, London, UK.

[4]Diamond Light Source, Didcot, UK.

[5]Leverhulme Research Centre for Functional Materials Design, University of Liverpool, Liverpool, UK.

These authors contributed equally: Ai He, Zhiwei Jiang.

*Corresponding authors. Email: a.livingtson@qmul.ac.uk; aicooper@liverpool.ac.uk

This PDF file includes:

1.0 Materials and Methods

2.0 Materials Characterisation

Tables S1 to S9

Figures S1 to S47

3.0 Additional Discussion

Figures S48 to S51

4.0 References

1.0 Materials and Methods

1.1 Materials.

1,3,5-Triformylbenzene (TFB) was purchased from Manchester Organics, UK. (1*R*,2*R*)-1,2-diaminocyclohexane (CHDA), reactive red 120 (RR, 1470 $g·mol^{-1}$), direct red 80 (DR, 1373 $g·mol^{-1}$), rose bengal (RB, 1018 $g·mol^{-1}$), brilliant blue (BB, 826 $g·mol^{-1}$), Congo red (CR, 697 $g·mol^{-1}$), protoporphyrin IX disodium (PPIX, 607 $g·mol^{-1}$), acid fuchsin (ACF, 585 $g·mol^{-1}$), sunset yellow (SY, 452 $g·mol^{-1}$), methyl orange (MO, 327 $g·mol^{-1}$), neutral red (NR, 289 $g·mol^{-1}$), 4-nitrophenol (NP, 139 $g·mol^{-1}$), and all solvents (HPLC grade) were purchased from Sigma-Aldrich. The commercially available membranes, SolSep-NF030306, SolSep-169, and SolSep-NF010206 were supplied by SolSep, Netherland; MPF-50 were supplied by Koch Membrane Systems, US; HITK-T1 was manufactured by Hermsdorfer Institut für Technische Keramik, Germany; and FSTi-128 was manufactured by Flemish Institute for Technological Research, Belgium. All materials were used as received.

1.2 Membrane fabrication

Fabrication of polyacrylonitrile (PAN) supports via phase inversion. Polyacrylonitrile (PAN) support membranes were cast using a continuous casting machine (Sepratek, South Korea). The dope solution comprised 11 wt.% polyacrylonitrile powder dissolved in a mixture of 44.5 wt.% dimethyl sulfoxide (DMSO) and 44.5 wt.% 1,3-dioxolane and allowed stirring overnight at 75 °C. After cooling down to room temperature and degassing, the PAN membrane was then cast onto a PET non-woven fabric (Hirose RO grade). The casting speed was controlled by the winder tension at 4 rpm with a knife gap 120 µm. After casting, the support was immediately immersed into a water bath at 60 °C for 3 hours, followed by drying at room temperature.

Fabrication of crystalline CC3 film. Continuous crystalline cage films of **CC3** were produced using an interfacial reaction and crystallisation process that occurred at the interface between an immiscible aqueous phase and an organic phase as presented in the Methods section. The continuous crystalline **CC3** film that grew at the dichloromethane-water interface was then isolated as a free-standing film that could then be adhered directly onto different substrates, including glass, carbon tape, steel mesh, silicon wafer, and anodic aluminum oxide for further analysis and to determine crystallinity and surface morphology (**Figure S1**).

The following procedure was used to produce the continuous crystalline composite **CC3**-PAN membranes for the liquid permeation studies: A PAN sheet covered a sintered disc at the bottom of an AdvanTec® glass filtration funnel with an effective diameter of 7.4 cm. The interfacial reaction was carried out as previously described in the Methods section. After 24 hours, the solvent was removed by filtration (**Figure S47**), which enabled the **CC3** film to be transferred directly onto the PAN support (**Figure S2**). For the membrane thickness analysis measurements performed using AFM, we placed a silicon wafer disc on top of the PAN support and used the same reaction procedure. After removing the

solvent by filtration, the **CC3** film was transferred onto a silicon wafer. Note, the diameter of the silicon wafer was <5.0 cm so that the solvent could be removed by filtration.

Fabrication of amorphous CC3 membrane via a spin-coating method. The amorphous **CC3** membrane was fabricated using a WS-650Mz-23NPP spin-coater (Laurell, USA). A **CC3** chloroform solution was prepared by sonicating/stirring **CC3** (50 mg) in chloroform (5 g, 3.7 mL) for 10 minutes. The suspension was then filtered using a PTFE syringe filter (0.2 μm). The edges of a 6×6 cm sized PAN membrane piece was taped to a glass slide that was then attached to the spin-coater by suction. The saturated **CC3** chloroform solution (1 mL) was added dropwise onto the membrane. After the addition, the slide was rotated at 500 rpm for 15 seconds and then 1500 rpm for 45 seconds. The amorphous **CC3** membrane was cut into 4.7 cm for the filtration experiment. The membrane was subjected to analysis by PXRD (**Figure S10b**).

1.3 Characterisation Methods

Nuclear Magnetic Resonance (NMR). NMR spectra were recorded on a Bruker 400 NMR spectrometer at 400 MHz (^{1}H) and referenced against the residual ^{1}H signal of the solvent.

Fourier Transform Infrared Spectroscopy (FT-IR). FT-IR spectra were recorded on a Bruker Tensor 27 spectrometer. Samples were cut into size and recorded in transmission mode using 16 scans with a resolution of 4 cm^{-1}.

Raman Mapping. Raman spectroscopy was performed using an inVia™ Reflex® Qontor Confocal Raman Microscope. Images of the **CC3** films were captured using the 50x objective and the WiRE spectral acquisition wizard was used for data collections and spectral analysis. Spectral analysis was performed using the following settings: 785 nm laser (power 5%); 1200 l/mm (633/780) grating; Renishaw Centrus 5134M5 detector; 50x L objective; 10 seconds exposure time with 3 accumulations; line focus mode; and spectra were recorded over the range 102 cm^{-1} to 3202 cm^{-1}. Cosmic ray deletion was performed using the WiRE software before spectra were analysed. The Raman map was performed by integrating multiple scans over the target, using a step size of 1×1 pixel. For the Raman maps, crystalline **CC3-α**[1] and amorphous **CC3**[2] samples were used as references.

Scanning Electron Microscopy (SEM). High-resolution images of the **CC3** films were recorded using a Hitachi S-4800 cold field emission scanning electron microscope (FE-SEM). The samples were appended to 15 mm Hitachi M4 aluminium stubs using either silver dag or an adhesive high purity carbon tab. The samples were then coated with a 2 nm layer of gold (Au) or cadmium using an Emitech K550X automated sputter coater. The FE-SEM measurement scale bar was calibrated using a certified SIRA calibration standard. Imaging was conducted at a working distance of 8 mm and a voltage of 3 kV using a mix of upper and lower secondary electron detectors.

Focused Ion Beam Scanning Electron Microscopy (FIB-SEM). The cross-sectional structure of the CC3 films was studied using a Tescan S8000G FIB-SEM. The specimens were attached to aluminum

(Al) stubs using carbon tabs, and silver (Ag) dag was applied to aid conductivity. After the dag had dried, the specimens were Au-coated using a Quorum Q150T ES coater (~10nm). The specimens were studied at a working distance of 6mm, tilted at 55 degrees from horizontal (so normal to the ion beam), using the ion beam for trenching and polishing (30kV and 1nA for trenching, and 30kV and 250pA for polishing) and the electron beam for imaging (5kV, 30pA, and 10kV, 60pA). Surfaces to be sectioned were coated with a protective layer of platinum (Pt), ~18um long by ~3um wide by ~1um thick, before milling (deposited using the ion beam at 30kV, 150pA). Micrographs were acquired using ultra-high-resolution mode at various stages of the study using the ET detector (two micrographs were also taken using the LEBSE detector).

Atomic Force Microscope (AFM). AFM imaging was used to characterise the morphology of the surface of the **CC3** films with a Multimode 8 (Bruker, CA, USA) model equipped with E - type or J - type pizzo scanner. The samples were attached to a magnetic sample disk and scanned under tapping mode using PointProbe® Plus silicon-SPM probes (PPP-NCH, Nanosensors™, Switzerland) with a typical tip radius of less than 7 nm and the cantilever resonance frequency was in the range of 204 - 497 kHz with a nominal spring constant of 42 $N \cdot m^{-1}$. Raster scanning was performed at a speed of 0.50 lines per second with a resolution of 512 points per line at a speed of 0.2 – 1.0 Hz. Gwyddion 2.44 SPM data visualisation and analysis software was used to process the AFM images. To measure film thickness, free-standing **CC3** films were transferred to silicon wafers and dried. A scratch was made to expose the wafer surface and allow measurements of the height from the silicon wafer surface to the **CC3** film surface. The thickness was estimated from the height difference between the silicon wafer and the **CC3** film using a one-dimensional statistical function.

Water Contact Angle (WCA) Measurements. WCA measurements were performed using a DSA100 expert drop shape analyser with the following measurement conditions, drop phase: water; surrounding phase: air; drop type: sessile drop; drop volume: 4.15 ± 0.5 μL; fitting method: Young–Laplace equation. Before recording these measurements, the samples were cut into 1×4 cm sized pieces and appended onto glass slides using double-sided tape.

Powder X-ray Diffraction (PXRD). Laboratory PXRD patterns were collected in transmission mode on samples held on thin Mylar film in aluminium well plates on a Panalytical Empyrean diffractometer, equipped with a high throughput screening XYZ stage, X-ray focusing mirror, and PIXcel detector, using Cu-Kα (λ = 1.541 Å) radiation. PXRD patterns were measured over the 2θ range 4-50° in 0.013° steps for 30 minutes. High-resolution synchrotron PXRD patterns were collected using the I11 beamline at Diamond Light Source (λ = 0.827 Å), which is equipped with a Mythen II position-sensitive detector[3,4]. Samples recorded at Diamond Light Source were loaded in borosilicate glass capillaries that were spun to improve powder averaging during data acquisition. PXRD patterns were refined by Pawley refinement in TOPAS Academic[5].

1.4 Membrane performance evaluation

Reversible filtration test. For the reversible filtration tests, feedstocks that contained BB dissolved in water and MeOH at a concentration of 20 ppm were used in the six-step procedure decried below. The dye concentrations in the permeate and retentate are listed in **Table S5**:

1) A BB water solution (20 ppm, 0.2 L) was added to the filtration cell, the cell was pressurised to 10 bar under nitrogen, and ~0.1 L of the feedstock was permeated through the **CC3**-PAN membrane.
2) The water permeate was collected to determine the dye concentration, and the remaining retentate solution was removed from the filtration cell.
3) The filtration cell, including the **CC3**-PAN membrane, was washed with MeOH (0.1 L) and air-dried.
4) A BB MeOH solution (20 ppm, 0.2 L) was added to the filtration cell, the cell was pressurised to 10 bar under nitrogen, and ~0.1 L of the feedstock was permeated through the **CC3**-PAN membrane.
5) The MeOH permeate was collected to determine the dye concentration, and the remaining retentate solution was removed from the filtration cell.
6) The filtration cell, including the **CC3**-PAN membrane, was washed with MeOH (0.1 L) and air-dried.

Here, we define the steps described above as "one cycle", and multiple cycles were performed using the same procedure, with the exact experimental details listed in **Table S5**.

To visualise the filtration process in real-time, we used a commercial bench-scale 50 mL transparent Merck Millipore Amicon® dead-end stirred cell which is connected to an 800 mL Merck Millipore Amicon® RC800 reservoir with a modified procedure: As shown in **Figure S23**, a **CC3**α-PAN membrane sample with a diameter of 42 mm was fixed onto the support at the base of the cell. The cell was then sealed using an O-ring and pressurised to 3 bar under nitrogen. To perform the filtration measurements, 50 mL of BB water feedstock (20 ppm dye concentration) was added to the feedstock tank. The solvent permeance was then calculated based on the amount of time it took a certain amount of dye feedstock to flow through the membrane, and the rejection was calculated using the dye concentration in feed and permeate. When the 50 mL feed permeated the membrane, another 50 mL of BB MeOH feedstock (20 ppm dye concentration) was added into the tank without further washing of the filtration system. The permeate was then collected, and the permeance and rejection of the membrane were calculated using the same equation. Multiple cycles were performed with the same procedure. A continual agitation speed of 400 rpm was used during the filtration experiments.

Membrane absorption test. Mass balance calculations were used to confirm that dye rejections were due to separation processes instead of dye adsorption by the **CC3**-PAN membrane. Membrane samples of **CC3**-PAN with a diameter of 4.7 cm were also immersed in 100 mL of a 20 ppm BB dye solution for

one week and the dye concentration in the solution was monitored by UV-vis absorption to confirm that the dyes were not absorbed by the membrane. 100 mg powder crystals of **CC3**α were also immersed into aqueous solutions containing 20 ppm dyes (100 mL) for one week to check whether the bulk powders absorb the dyes.

Long-term operation. Long-term evaluation of the **CC3**-PAN membrane performance was tested in a 50 mL Merck Millipore Amicon® dead-end stirred cell which is connected to an 800 mL Merck Millipore Amicon® RC800 reservoir. ~1 L rose bengal aqueous solution (20 ppm) was added into both cells, and a **CC3**α-PAN membrane sample with a diameter of 42 mm was fixed on the support at the base of the cell, and sealed using an O-ring. To enable to operate the dye filtration process in a longer time (*e.g.*, 20 hours), the feedstocks were kept under lower pressure (1 bar) at room temperature, and the feedstock was continually stirred using a stirrer bar rotating at 400 rpm.

Membrane stability test. Freshly prepared water feedstock solutions that contained 20 ppm of BB were used to determine the membrane continuity and stability after measuring its organic solvent permeance. By measuring rejection performance before and after the solvent permeances, we could determine that the membrane remained intact during the organic solvent permeance measurements.

Water and MeOH feedstock mixture separation measurements. Separations performed with varying water:MeOH volumetric ratios (9:1, 8:2, 7:3, 6:4, 5:5, 4:6, 3:7, 2:8, 1:9) that contained 20 ppm of BB were used to determine the dynamic behaviour of the **CC3**-PAN membrane. Separately, 0.2 L of each feedstock was charged into the dead-end stirred cell, and the cell was pressurised to 10 bar. 0.1 L of permeate was collected, and its UV-vis absorption spectrum was recorded to calculate the dye rejection % using *Eq.* 3. Between each measurement, the surface of the **CC3**-PAN membrane was washed with pure water:MeOH mixtures that matched the solvent ratio used during the subsequent measurement. The calculated dye rejection % *versus* water content in MeOH is shown in **Figure 5**. By programing in R language, the S-shaped curve (sigmoid curve) was found to fit the logistic function:

$$y = \frac{1}{1+e^{-k\cdot(x-x_0)}} \qquad (Eq.S1)$$

where k is the logistic growth rate (steepness of the curve), and x_0 is the x value of the sigmoid midpoint. The parameters k and x_0 were calculated using Nonlinear Least Squares (NLS) function in R languange[6].

Graded sieving experiments. Three-component graded sieving dye separations were carried out using DR, BB, and NP dyes in MeOH and the following experimental procedure:

1) 100 mL of a MeOH feedstock that contained 20 ppm of the dyes DR, BB, and NP was added to the dead-end stirred cell. The cell was pressurised to 10 bar under nitrogen, and 50 mL of permeate was collected.
2) DR, BB, and NP have different UV absorption wavelengths (DR: 528 nm, BB: 586 nm, NP: 312 nm, in MeOH), enabling the rejection of each dye to be calculated using *Eq.* 3 and the UV-

absorption spectra of the permeate. The remaining MeOH feedstock was removed for the dead-end stirred cell and discarded.

3) To 10 mL of the MeOH permeate, 90 mL of water was added to generate a 10/90 vol/vol MeOH/water feedstock, which was subsequently was added into the dead-end stirred cell. The cell was pressurised to 10 bar under nitrogen, and 50 mL of permeate was collected.
4) Due to the dilution of MeOH feedstock after adding 90% by volume of water, we concentrated the 50 mL of MeOH/water permeate to 5 mL before recording its UV-absorption spectra. UV-absorption data for the as-collected dilute and concentrated permeate are listed in **Table S8**. The rejection of each dye was then calculated using *Eq*. 3.

Ternary separation experiment. Three-component dye separations were carried out using DR, BB, and NP dyes in water and the experimental procedure listed below. The experiment was performed using a commercial bench-scale 50 mL transparent Merck Millipore Amicon® dead-end stirred cell connected to an 800 mL Merck Millipore Amicon® RC800 reservoir. No intermediate handling of the membrane or feedstock was undertaken during the molecular separation.

1) 100 mL of a water feedstock that contained 20 ppm of the dyes, DR, BB, and NP was added to the transparent dead-end stirred cell. The cell was pressurised to 3 bar under nitrogen, and 90 mL of permeate was collected.
2) DR, BB, and NP have different UV absorption wavelengths (DR: 528 nm, BB: 551 nm, NP: 317 nm, in water), enabling the rejection of each dye to be calculated using *Eq*. 3 and the UV-absorption spectra of the permeate.
3) 10 mL of feedstock was kept in the cell. To wash out the remaining NP from the retentate, 90 mL of water was added into the cell, and another 90 mL of permeate was collected. Step 3 was repeated until the NP concentration in the retentate was < 1%, as determined by UV-vis absorption.
4) 90 mL of MeOH was then added into the 10 mL of water retentate to form a new feedstock that contained 90 vol.% of MeOH. The cell was pressurised to 3 bar under nitrogen and 90 mL of the permeate was collected.
5) The rejection of each dye was calculated using *Eq*. 3 and the UV absorption spectra of the permeate.
6) 10 mL of the water/MeOH feedstock was kept in the cell. To wash out the remaining BB from the retentate, 90 mL of MeOH was added into the cell, and another 90 mL of permeate was collected. Step 3 was repeated with MeOH until the BB concentration in the retentate was < 1%, as determined by UV-vis absorption.

2.0 Materials Characterisation

Table S1. Organic dye molecules used in the membrane performance experiments.

Organic Dyes	Molecular Weight (g·mol^{-1})	Molecular Structure	Absorbance Wavelength (nm)[a]
**Reactive Red 120 (RR)**	1470	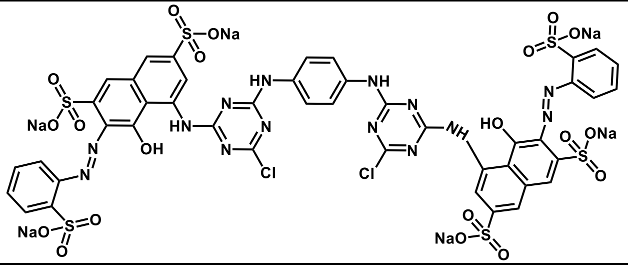	540
**Direct Red 80 (DR)**	1373	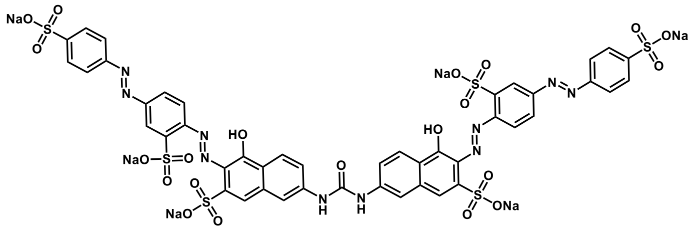	528
**Rose Bengal (RB)**	1018	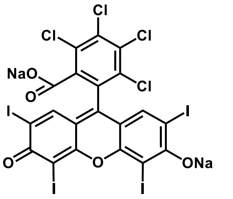	549
**Brilliant Blue (BB)**	826	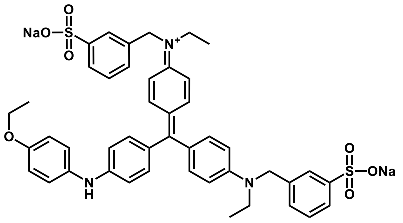	551
**Congo Red (CR)**	697	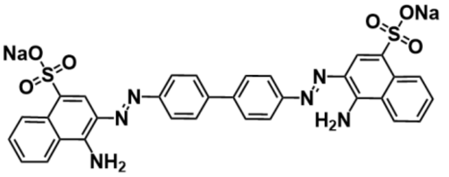	498
**Protopor-phyrin IX Disodium (PP IX)**	607	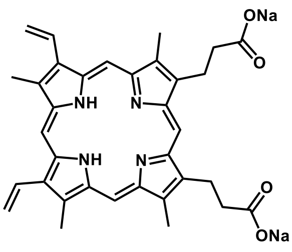	366

[a]wavelength of maximum absorbance in water.

Table S1 (cont.). Organic dye molecules used in the membrane performance experiments.

Organic Dyes	Molecular Weight (g/mol)	Molecular Structure	Absorbance Wavelength (nm)[a]
**Acid Fuchsin (ACF)**	585	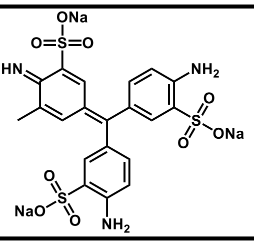	546
**Sunset Yellow (SY)**	452	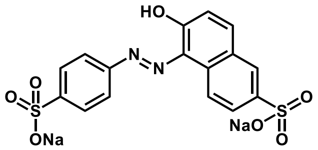	482
**Methyl Orange (MO)**	327	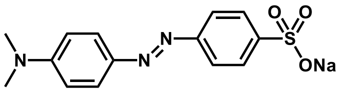	465
**Neutral Red (NR)**	289	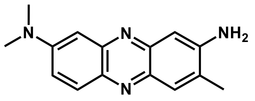	520
**4-Nitrophenol (NP)**	139	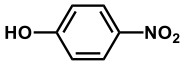	317

[a]wavelength of maximum absorbance in water.

Table S2. Hansen solubility parameter (δ) and the physical properties of the organic solvents that were used to determine solvent permeance for **CC3**-PAN.

Solvents	Molecular weight $(g{\cdot}mol^{-1})$*	†Hansen solubility parameter $(MPa^{1/2})$			Molar volume (V_m)‡ $(cm^3\ mol^{-1})$	d_m§ (nm)	Viscosity at 30 °C $(\times 10^{-3}\ Pa{\cdot}S)$
		δ_d	δ_p	δ_h			
Water	18.02	15.5	16.0	42.3	18.0	0.39	0.80^{A}
MeOH	32.04	14.7	12.3	22.3	40.7	0.51	0.49^{B}
Ethanol	46.07	15.8	8.8	19.4	58.7	0.57	1.17^{Γ}
Acetonitrile	41.05	15.3	18.0	6.1	52.9	0.55	0.32^{B}
Acetone	58.08	15.5	10.4	7.0	73.9	0.62	0.29^{Γ}
Hexane	86.18	14.9	0	0	131.4	0.75	0.28^{Δ}
Heptane	100.21	15.3	0	0	147.5	0.78	0.33^{Γ}
Toluene	92.13	18	1.4	2	106.9	0.70	0.52^{Γ}

* Molecular weight taken from CRC Handbook of Chemistry and Physics, see reference[7].

† Hansen solubility parameter, δ_d = solubility parameter due to dispersion forces, δ_p = solubility parameter due to dipole forces, and δ_h = solubility parameter due to hydrogen bonding (or in general due to donor-acceptor interactions). See reference[8].

‡ See reference[9,10].

§ Molar diameter (d_m) was calculated from reference[11] using molar volume (V_m) of the solvent molecule: $d_m = 2 \times (3V_m/4\pi N_A)^{1/3}$; where N_A is the Avogardo constant.

A See reference[12].

B Calculated from the Lewis and Squires chart[10,13].

Γ Calculated from the experimental values given in reference[10].

Δ See reference[14].

Table S3. Dye rejection measurement data for **CC3**-PAN determined using water and MeOH feedstocks.

Solvent	Dye	Sample Collected	Volume (mL)	Wavelength[a] (nm)	Absorption	Rejection (%)	Ref. Figure
Water	RR	Feed	100	536	1.00	96.10 ± 2.80	Fig. S30 (a)
		Permeate	50	538	0.05		
		Retentate	50	538	1.91		
Water	DR	Feed	120	528	1.28	98.21 ± 1.52	Fig. S30 (b)
		Permeate	59	528	0.03		
		Retentate	61	530	2.47		
Water	RB	Feed	100	549	1.66	97.11 ± 0.56	Fig. S30 (c)
		Permeate	54	551	0.03		
		Retentate	46	550	3.53		
Water	BB	Feed	120	551	1.42	99.89 ± 0.05	Fig. S30 (d)
		Permeate	57	551	0.02		
		Retentate	63	551	2.67		
Water	CR	Feed	100	498	1.24	99.73 ± 0.12	Fig. 3f
		Permeate	52	498	0.02		
		Retentate	48	498	2.53		
Water	PPIX	Feed	120	366	1.13	99.96 ± 0.03	Fig. S30 (e)
		Permeate	66	366	0.01		
		Retentate	54	368	2.47		
Water	ACF	Feed	100	546	1.69	62.42 ± 3.40	Fig. S30 (f)
		Permeate	54	546	0.64		
		Retentate	46	548	2.68		
Water	SY	Feed	100	482	1.59	51.14 ± 3.12	Fig. S30 (g)
		Permeate	54	482	0.72		
		Retentate	46	482	2.00		
Water	MO	Feed	100	465	2.12	33.07 ± 2.66	Fig. S30 (h)
		Permeate	49	464	1.56		
		Retentate	51	464	2.37		
Water	NR	Feed	100	520	2.11	22.43 ± 3.82	Fig. S30 (i)
		Permeate[b]	52	520	0.63		
				455	0.44		
		Retentate	48	520	2.79		
Water	NP	Feed	100	317	2.07	14.17 ± 2.05	Fig. S30 (j)
		Permeate	45	318	1.70		
		Retentate	55	318	2.19		
MeOH	RR	Feed	100	540	1.11	92.78 ± 3.10	Fig. S39 (a)
		Permeate	52	538	0.07		
		Retentate	48	538	2.41		

Table S3 continues on following page S12.

Table S3 (cont.). Dye rejection measurement data for **CC3**-PAN determined using water and MeOH feedstocks.

Solvent	Dye	Sample Collected	Volume (mL)	Wavelength[a] (nm)	Absorption	Rejection (%)	Ref. Figure
MeOH	DR	Feed	100	526	1.36	93.02 ± 2.54	Fig. S39 (b)
		Permeate	52	528	0.07		
		Retentate	48	528	2.42		
MeOH	RB	Feed	100	558	2.21	2.52 ± 2.05	Fig. S38 (c)
		Permeate	57	558	2.08		
		Retentate	43	558	2.50		
MeOH	BB	Feed	100	588	1.98	0.76 ± 0.26	Fig. S39 (d)
		Permeate	51	588	1.96		
		Retentate	49	587	2.03		
MeOH	CR	Feed	100	502	1.46	3.70 ± 2.47	Fig. S39 (e)
		Permeate	46	502	1.38		
		Retentate	54	504	1.52		
MeOH	PPIX	Feed	120	394	3.51	5.96 ± 3.41	Fig. S39 (f)
		Permeate	55	394	3.33		
		Retentate	65	394	3.60		
MeOH	ACF	Feed	100	552	2.57	5.21 ± 3.02	Fig. 39(g)
		Permeate	58	552	2.37		
		Retentate	42	553	2.73		
MeOH	SY	Feed	100	481	1.79	3.24 ± 2.63	Fig. S39 (h)
		Permeate	59	480	1.63		
		Retentate	41	480	1.87		
MeOH	MO	Feed	100	422	2.76	4.05 ± 2.95	Fig. S39 (i)
		Permeate	52	422	2.64		
		Retentate	48	424	2.85		
MeOH	NR	Feed	100	534	3.51	4.82 ± 3.28	Fig. S39 (j)
		Permeate[b]	46	536	1.69		
				466	1.18		
		Retentate	54	536	4.56		
MeOH	NP	Feed	100	311	2.14	2.77 ± 2.05	Fig. S39 (k)
		Permeate	55	312	2.13		
		Retentate	45	310	2.14		

[a] Wavelength of maximum absorbance. [b] Wavelength of maximum absorbance of the peak at the red colour absorbance (520 nm) and orange colour absorbance (455 nm). As NR is a pH indicator changing from red to yellow between pH 6.8 and 8.0, the dye concentration was calculated using the absorbance of both yellow and red peaks.

Table S4. Solvent permeance data for **CC3**-PAN.

Solvent	Time (min)	Permeated Volume (mL)	Permeance ($L.m^{-2}.h^{-1}.bar^{-1}$)	Ref. Figure
Water	2	30.0	43.0 ± 4.20	Fig.3a [2]
	6	72.5		
	10	112.0		
	28	281.5		
	40	359.5		
MeOH	2	40.0	85.1 ± 5.68	Fig. 4c
	5	97.0		
	10	174.0		
	21	383.5		
	30	534.5		
Ethanol	2	15.0	30.1 ± 5.25	Fig. 3a [1]
	6	43.5		
	12	84.0		
	25	162.5		
	40	252.0		
Acetonitrile	2	67.5	147.5 ± 9.57	Fig. 3a [6]
	5	158.0		
	10	312.5		
	15	465.0		
	20	617.5		
Acetone	1	39.5	177.4 ± 10.23	Fig. 3a [7]
	3	114.0		
	6	226.0		
	10	374.0		
	15	556.9		

Table S4 continues on following page S14.

Table S4 (cont.). Solvent permeance data for **CC3**-PAN.

Hexane	1	32.0	136.86 ± 10.89	Fig. 3a [5]
	5	151.5		
	10	294.0		
	14	406.0		
	20	573.0		
Heptane	2	48.0	96.02 ± 8.45	Fig. 3a [4]
	5	107.5		
	10	208.5		
	18	369.0		
	25	502.5		
Toluene	3	40.5	55.89 ± 6.03	Fig. 3a [3]
	5	65.5		
	11	137.5		
	20	242.0		
	30	351.0		

Table S5. Reversible dye rejection data for **CC3**-PAN, recorded while switching the feedstock between 20 ppm BB dissolved in water and MeOH feedstocks.

Cycle	Solvent	Dye	Sample Collected	Volume (mL)	Wavelength (nm)	Absorption	Rejection (%)
1	Water	BB	Feed	110	552	1.3823	
			Permeate 1	11	551	0.0018	99.87
			Permeate 2	13	551	0.0006	99.96
			Permeate 3	14	551	0.0018	99.87
			Permeate 4	12	551	0.0021	99.85
			Permeate 5	9	551	0.0028	99.80
			Retentate	51	551	2.2893	
1	MeOH	BB	Feed	110	587	1.6239	
			Permeate 1	9	587	1.6090	0.92
			Permeate 2	15	587	1.6041	1.22
			Permeate 3	10	587	1.595	1.78
			Permeate 4	11	587	1.5935	1.87
			Permeate 5	10	587	1.6077	1.00
			Retentate	55	587	1.7284	
2	Water	BB	Feed	100	551	1.2468	
			Permeate 1	6	551	0.0023	99.83
			Permeate 2	11	551	0.0025	99.82
			Permeate 3	8	551	0.0023	99.83
			Permeate 4	10	551	0.0017	99.88
			Permeate 5	12	551	0.0023	99.83
			Retentate	53	552	2.7621	
2	MeOH	BB	Feed	100	587	1.6959	
			Permeate 1	12	587	1.6811	0.87
			Permeate 2	10	587	1.6922	0.22
			Permeate 3	18	587	1.6827	0.78
			Permeate 4	6	586	1.6723	1.39
			Permeate 5	7	586	1.6906	0.31
			Retentate	47	587	1.7140	
3	Water	BB	Feed	100	552	1.3208	
			Permeate 1	17	552	0.0018	99.86
			Permeate 2	15	552	0.0020	99.85
			Permeate 3	11	551	0.0037	99.72
			Permeate 4	7	551	0.0021	99.84
			Permeate 5	8	551	0.0025	99.81
			Retentate	42	551	3.1182	

Table S5 continues on following page S16.

3	MeOH	BB	Feed	100	587	1.6648	
			Permeate 1	12	587	1.6410	1.43
			Permeate 2	21	586	1.6134	3.09
			Permeate 3	9	587	1.6263	2.31
			Permeate 4	11	587	1.6275	2.24
			Permeate 5	6	587	1.6475	1.04
			Retentate	41	587	1.6976	
4	Water	BB	Feed	100	552	1.3546	
			Permeate 1	10	551	0.0023	99.83
			Permeate 2	8	551	0.0018	99.87
			Permeate 3	7	551	0.0018	99.87
			Permeate 4	8	551	0.0024	99.82
			Permeate 5	11	551	0.0020	99.85
			Retentate	56	552	2.4845	
4	MeOH	BB	Feed	100	587	1.6761	
			Permeate 1	15	586	1.6555	1.23
			Permeate 2	17	587	1.6587	1.04
			Permeate 3	10	587	1.6416	2.06
			Permeate 4	8	587	1.6733	0.17
			Permeate 5	6	587	1.6598	0.97
			Retentate	44	587	1.6664	
5	Water	BB	Feed	100	550	1.3024	
			Permeate 1	10	551	0.0001	99.99
			Permeate 2	15	551	0.0001	99.99
			Permeate 3	14	551	0.0035	99.73
			Permeate 4	7	551	0.0051	99.61
			Permeate 5	11	551	0.0014	99.89
			Retentate	43	551	2.8876	
5	MeOH	BB	Feed	100	587	1.6548	
			Permeate 1	21	586	1.6753	3.20
			Permeate 2	7	586	1.6584	2.00
			Permeate 3	6	587	1.6885	2.99
			Permeate 4	10	587	1.6823	1.23
			Permeate 5	11	587	1.6548	1.59
			Retentate	45	587	1.7529	
6	Water	BB	Feed	100	552	1.3620	
			Permeate 1	12	551	0.0022	99.84
			Permeate 2	13	552	0.0019	99.86
			Permeate 3	11	551	0.0020	99.85
			Permeate 4	8	551	0.0044	99.68
			Permeate 5	9	551	0.0034	99.75
			Retentate	47	551	2.5413	

Table S6. Reversible dye rejection data for three **CC3**-PAN samples, recorded while switching the feedstock between 20 ppm BB dissolved in water and MeOH feedstocks. The details of membrane sample I are shown in **Table S5**.

		Rejection for BB (%)			Average Value (%)	Standard Deviation (%)
Cycle	Solvent	Sample I	Sample II	Sample III		
1	Water	99.87	98.61	100.00	99.49	0.50
		99.96	98.99	99.35	99.43	0.40
		99.87	99.99	99.99	99.95	0.06
		99.85	99.99	99.98	99.94	0.07
		99.80	99.73	99.61	99.71	0.08
	MeOH	0.92	0.39	0.01	0.44	0.37
		1.22	0.02	0.41	0.55	0.50
		1.78	0.31	0.00	0.70	0.77
		1.87	0.54	0.68	1.03	0.60
		0.99	0.17	0.84	0.67	0.36
2	Water	99.83	99.35	99.99	99.72	0.27
		99.82	99.49	99.77	99.69	0.15
		99.83	99.77	99.99	99.86	0.09
		99.88	99.32	99.99	99.73	0.29
		99.83	99.12	99.71	99.55	0.31
	MeOH	0.87	0.32	0.71	0.63	0.23
		0.22	0.24	0.56	0.34	0.16
		0.78	0.87	0.01	0.55	0.39
		1.39	0.08	0.08	0.51	0.62
		0.31	0.85	1.25	0.80	0.38
3	Water	99.86	99.36	99.99	99.74	0.27
		99.85	99.50	99.92	99.76	0.18
		99.72	99.97	99.99	99.89	0.12
		99.84	99.99	99.96	99.93	0.07
		99.81	99.77	99.97	99.85	0.08
	MeOH	1.43	2.99	0.64	1.69	0.98
		3.09	2.33	1.20	2.21	0.77
		2.31	0.38	2.83	1.84	1.05
		2.24	0.01	2.97	1.74	1.26
		1.04	0.64	2.58	1.42	0.84

Table S6 continues on following page S18.

4	Water	99.83	99.32	99.08	99.41	0.31
		99.87	98.97	99.28	99.37	0.38
		99.87	99.99	99.98	99.95	0.05
		99.82	99.91	99.99	99.91	0.07
		99.85	99.90	99.16	99.64	0.34
	MeOH	1.23	0.02	0.58	0.61	0.49
		1.04	0.01	0.53	0.53	0.42
		2.06	0.02	1.18	1.08	0.84
		0.17	0.01	1.20	0.46	0.53
		0.97	0.01	2.83	1.27	1.17
5	Water	99.99	99.84	99.91	99.91	0.06
		99.99	99.86	99.99	99.95	0.06
		99.73	99.85	99.83	99.80	0.05
		99.61	99.99	99.68	99.76	0.16
		99.89	99.75	99.45	99.70	0.18
	MeOH	3.20	0.05	0.98	1.41	1.32
		2.00	0.35	1.32	1.22	0.68
		2.99	0.01	0.69	1.23	1.28
		1.23	0.01	1.34	0.86	0.60
		1.59	0.96	2.37	1.64	0.58

Table S7. Solvent permeance data for three **CC3**-PAN samples, recorded while switching the feedstock between 20 ppm BB dissolved in water and MeOH feedstocks.

		Permeance ($L.m^{-2}.h$-$1.bar^{-1}$)			Average Value ($L.m$-$^{2}.h^{-1}.bar^{-1}$)	Standard Deviation ($L.m^{-2}.h$-$1.bar^{-1}$)
Cycle	Solvent	Sample I	Sample II	Sample III		
1	Water	49.36	50.16	57.32	52.28	3.58
		45.60	52.55	54.94	51.03	3.96
		47.77	51.35	54.14	51.09	2.61
		42.87	50.16	52.55	48.53	4.12
		43.50	45.38	52.55	47.14	3.90
	MeOH	100.19	110.87	97.55	102.87	5.76
		106.62	100.89	94.16	100.56	5.09
		102.74	106.67	98.96	102.79	3.15
		102.63	109.23	98.27	103.38	4.51
		107.70	100.63	97.77	102.03	4.17
2	Water	55.32	48.88	45.51	49.90	4.07
		50.16	50.64	45.88	48.89	2.14
		47.38	52.32	48.77	49.49	2.08
		43.50	49.91	45.60	46.34	2.67
		42.99	51.74	45.79	46.84	3.65
	MeOH	95.54	93.15	107.48	98.72	6.27
		95.54	103.56	101.91	100.34	3.46
		98.72	104.97	95.54	99.74	3.92
		101.51	109.43	97.93	102.95	4.80
		100.70	104.20	98.31	101.07	2.42
3	Water	51.94	47.32	42.99	47.42	3.65
		51.14	43.95	43.44	46.18	3.52
		50.74	42.04	43.69	45.49	3.77
		50.95	43.22	44.99	46.39	3.31
		47.77	40.62	46.38	44.93	3.09
	MeOH	95.54	106.45	95.21	99.07	5.22
		95.54	105.09	98.82	99.82	3.96
		93.95	102.71	96.43	97.69	3.69
		97.76	100.16	92.61	96.84	3.15
		99.50	98.57	92.85	96.97	2.94

Table S7 continues on following page S20.

4	Water	45.38	42.35	48.60	45.44	2.55
		43.22	47.43	42.44	44.36	2.19
		39.44	43.72	45.03	42.73	2.39
		41.22	41.44	47.83	43.50	3.07
		38.83	45.43	43.44	42.57	2.77
	MeOH	95.54	90.32	100.32	95.39	4.08
		92.36	95.54	100.76	96.22	3.46
		88.85	95.99	97.77	94.20	3.85
		91.23	90.03	100.32	93.86	4.59
		90.63	92.05	97.93	93.54	3.16
5	Water	43.60	47.77	48.57	46.65	2.18
		40.83	41.40	47.77	43.33	3.15
		40.03	45.38	48.38	44.60	3.45
		38.44	42.99	47.80	43.08	3.82
		38.22	41.80	46.99	42.34	3.60
	MeOH	90.00	88.99	101.02	93.34	5.45
		98.60	88.60	100.76	95.99	5.30
		95.99	89.57	95.99	93.85	3.03
		97.18	92.76	93.12	94.35	2.00
		98.94	90.99	95.26	95.06	3.25

Table S8. Continuous dye rejection measurement data for **CC3**-PAN in water and MeOH/water mixture (90/10 vol/vol) while MeOH was added to the retentate solution in Step 2 of ternary molecular separation process.

Solvent	Sample Collected	Volume (mL)	Dye	Wavelength (nm)	Absorption	Concentration (%)	Outcome
Water	Feed	100	NP	310	3.41	33.33	
			BB	551	1.43	33.33	
			DR	540	1.64	33.33	
	Permeate	50	NP	310	3.28	98.08 ± 1.27	NP collected
			BB	551	0.01	0.79 ± 0.22	
			DR	540	0.02	1.12 ± 0.32	
MeOH/ Water (90/10 vol/vol)	Feed (Retentate)	100	NP	313	0.28	0.98 ± 0.09	
			BB	588	0.73	50.59 ± 1.36	
			DR	535	0.82	49.8 ± 1.02	
	Permeate	50	NP	313	0.21	0.001 ± 0.001	BB collected
			BB	305	0.25	99.56 ± 0.28	
				535	0.42		
				588	0.73		
			DR	530	0.40	0.44 ± 0.28	
	Retentate	50	NP	312	0.20	0.001 ± 0.001	DR collected
			BB	588	0.14	0.44 ± 0.28	
			DR	530	0.70	99.56 ± 0.28	

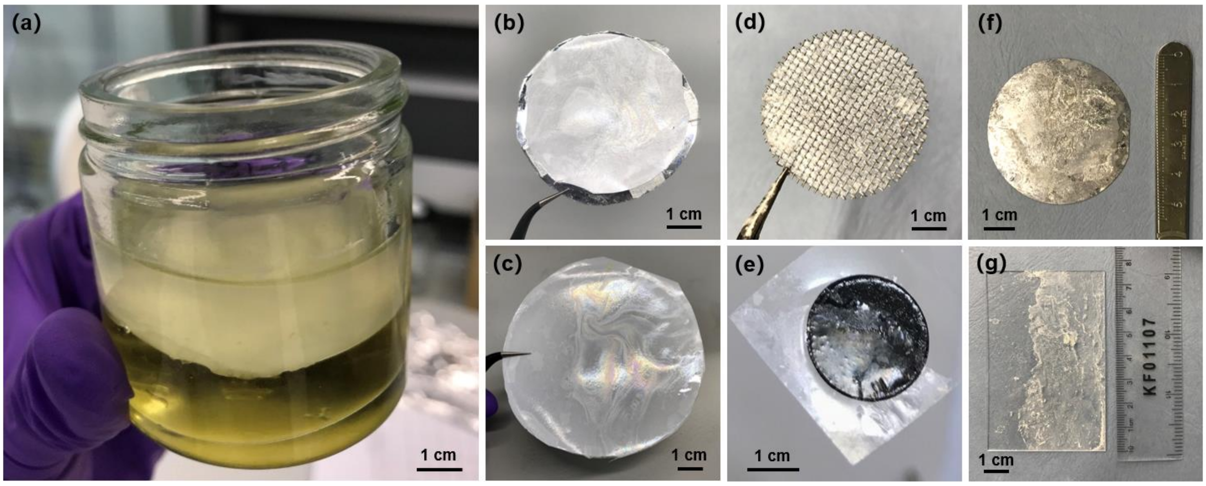

Figure S1. Photographs of as synthesised **CC3**α-PAN-24hr-0.8% film. (a) Free-standing **CC3** film grown in a glass dish at the liquid-liquid interface between water and dichloromethane. (b, c, d, e, f, g) **CC3** films transferred onto different substrates for further analysis: (b) PAN sheet fixed on an aluminum wafer; (c) PAN membrane; (d) stainless mesh; (e) carbon tape used for scanning electron microscopy; (f) silicon wafer; (g) glass slide.

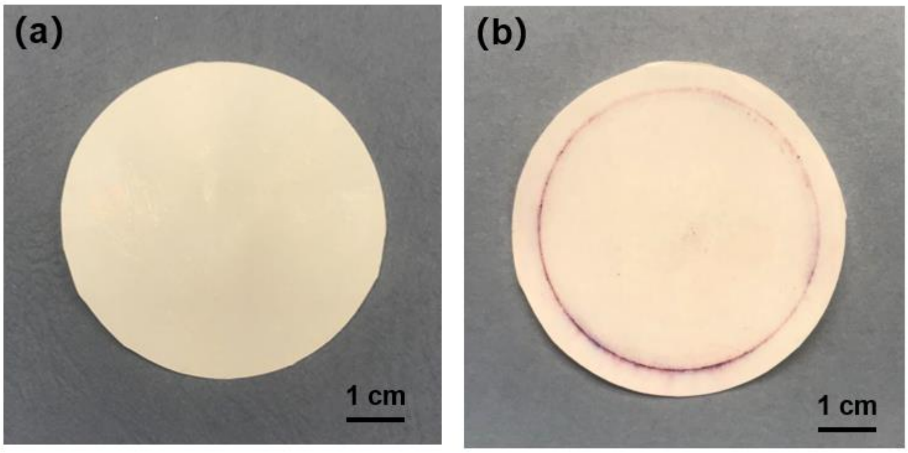

Figure S2. Photographs of **CC3**α-PAN-24hr-0.8% membrane. (a) As-prepared **CC3**α-PAN-24hr-0.8%, and (b) **CC3**α-PAN-24hr-0.8% after a series of dye filtration tests using a commercial bench-scale dead-end stirred-cell filtration unit (Sterlitech® HP4750 stirred cell) that was kept under 10 bar nitrogen atmosphere. The diameter of the filtration dish was 4.7 cm and a stirrer bar agitation speed of 400 rpm was used during the measurements.

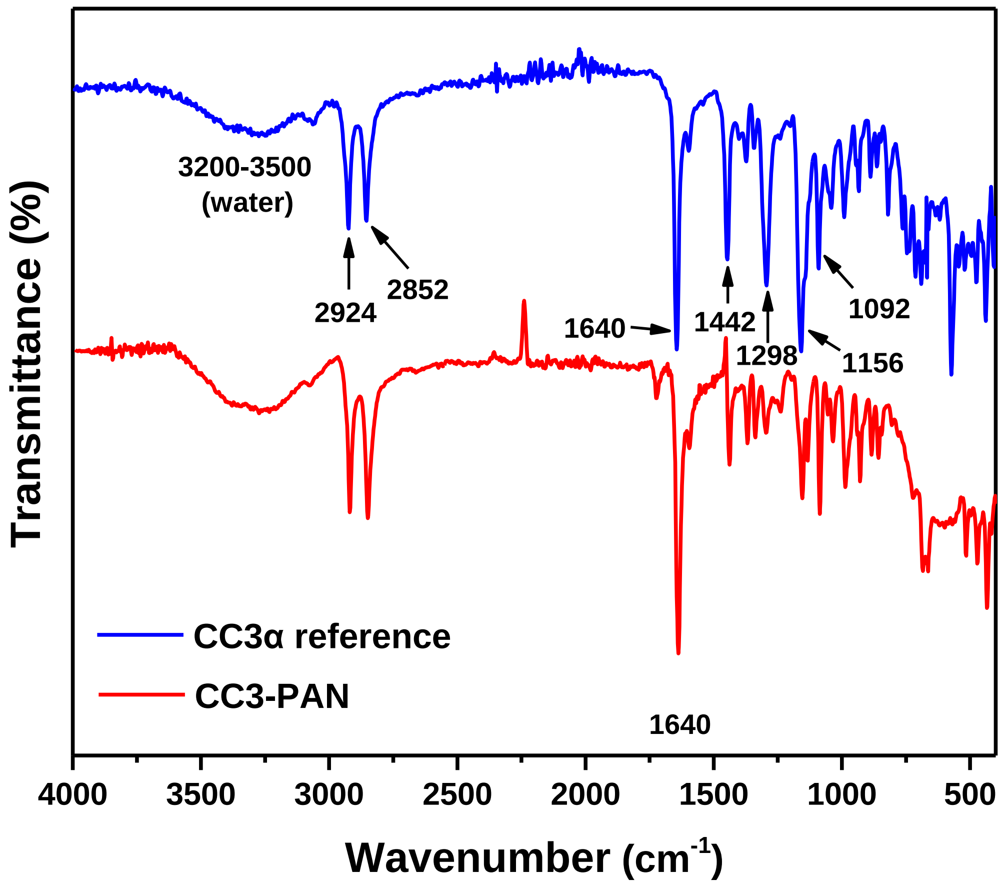

Figure S3. FT-IR spectra of **CC3**α-PAN-24hr-0.8% and crystalline **CC3**α[1] reference. The signal of blank PAN support was subtracted during the background measurement. Main peaks are labelled with the wavenumber values. Film synthesis conditions, reagent concentration: TFB 0.8 wt.% in DCM (30 mL), CHDA 0.8 wt.% in water (32 mL); reaction conditions: 24 hours at room temperature; dish diameter: 7.4 cm.

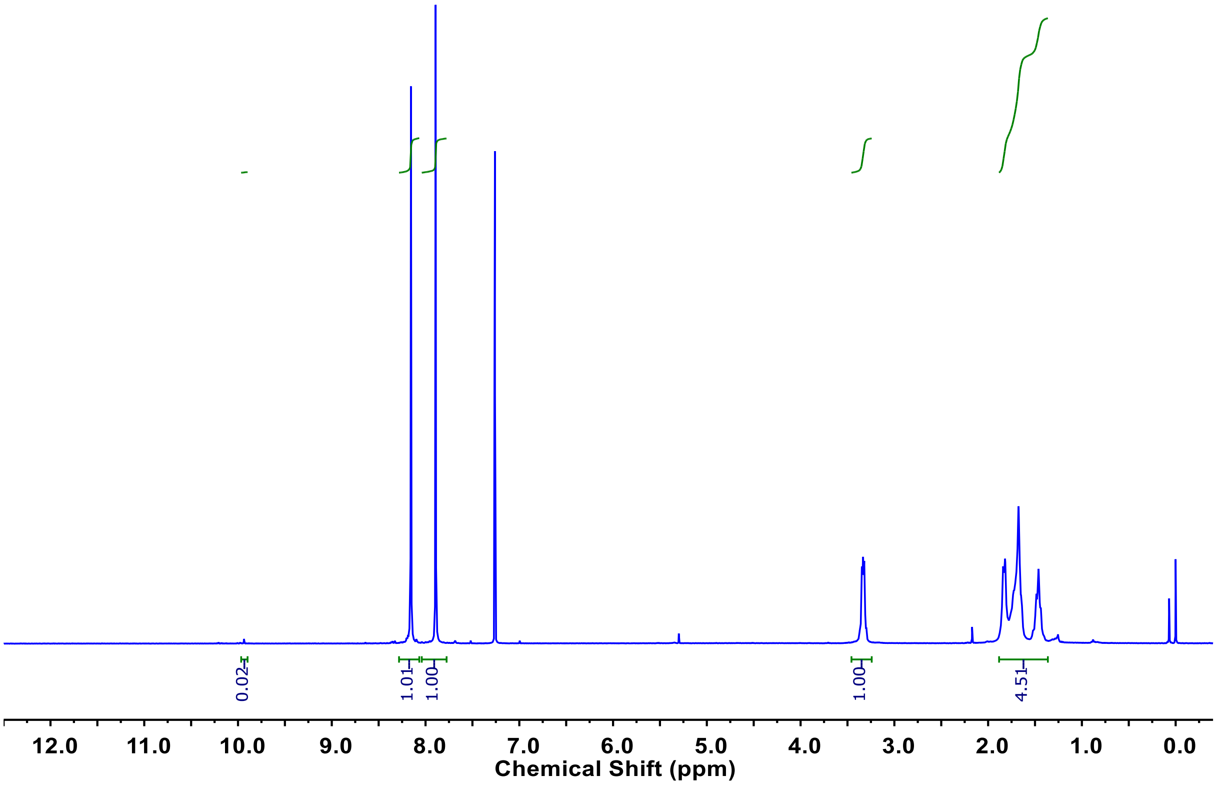

Figure S4. ^{1}H NMR (400 MHz, $CDCl_3$) spectrum for a solid sample of **CC3** film that had been deposited on glass and scraped off before being fully dissolved in $CDCl_3$: δ = 8.15 (s, CH=N, 12H), 7.89 (s, ArH, 12H), 3.33 (m, CHN, 12H), 1.9 – 1.4 (m, cyclohexyl CH and CH_2, 48H). Although the ^{1}H NMR spectrum was measured within 5 mins of dissolving the **CC3** film in $CDCl_3$, self-sorting of misaligned cage fragments can still occur on this timescale and can, therefore, not be ruled out. Film synthesis conditions, reagent concentration: TFB 0.8 wt.% in DCM (30 mL), CHDA 0.8 wt.% in water (32 mL); reaction conditions: 24 hours at room temperature; dish diameter: 7.4 cm.

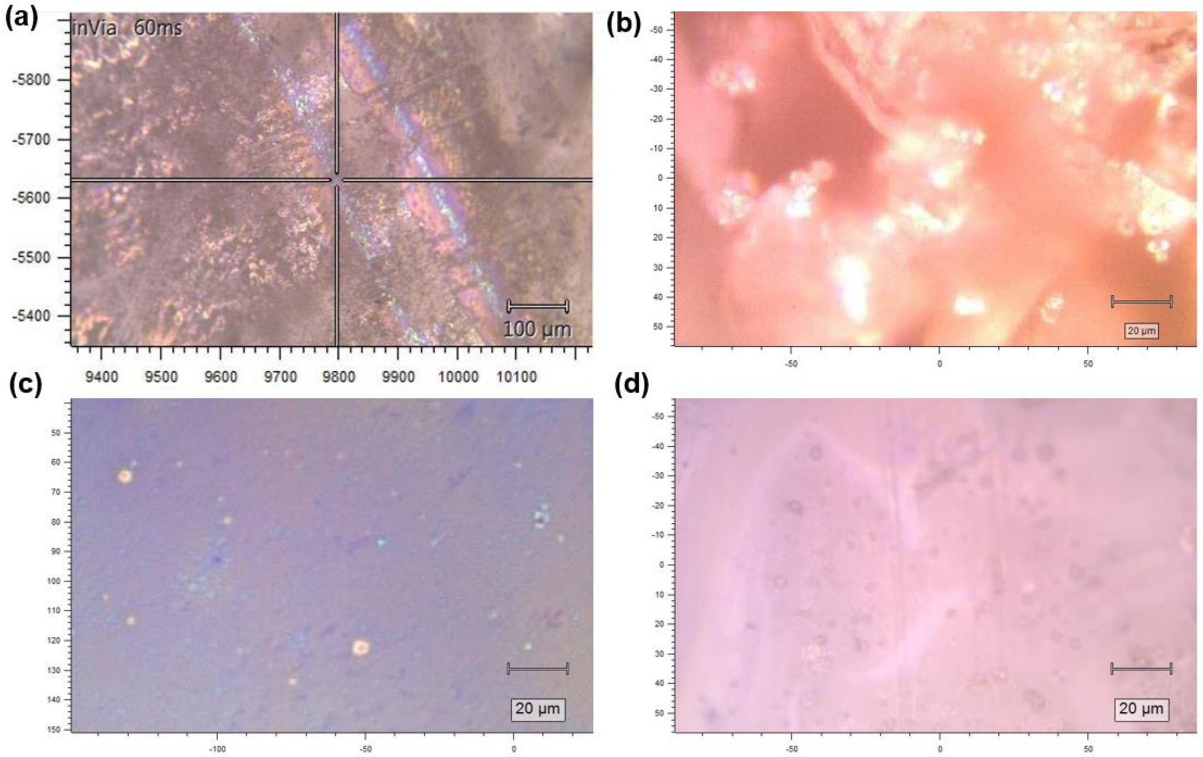

Figure S5. Microscopic images of **CC3** film deposited on a glass slide (50x objective), which indicate a homogenous surface morphology. Film synthesis conditions, reagent concentration: TFB 0.8 wt.% in DCM (30 mL), CHDA 0.8 wt.% in water (32 mL); reaction conditions: 24 hours at room temperature; dish diameter: 7.4 cm.

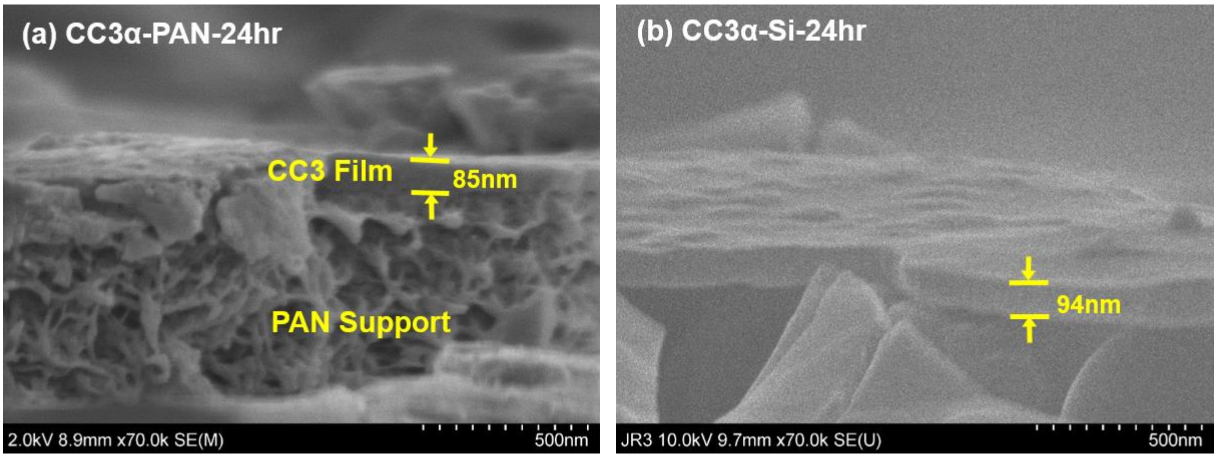

Figure S6. SEM images of continuous **CC3** film deposited on (a) PAN support (**CC3**α-PAN-24hr), and (b) silicon wafer (**CC3**α-Si-24hr), where the α symbol is used to denote the crystalline structure of the **CC3** film. The thicknesses of the **CC3**α films have been included in the images. Film synthesis conditions, reagent concentration: TFB 0.8 wt.% in DCM (30 mL), CHDA 0.8 wt.% in water (32 mL); reaction conditions: 24 hours at room temperature; dish diameter: 7.4 cm.

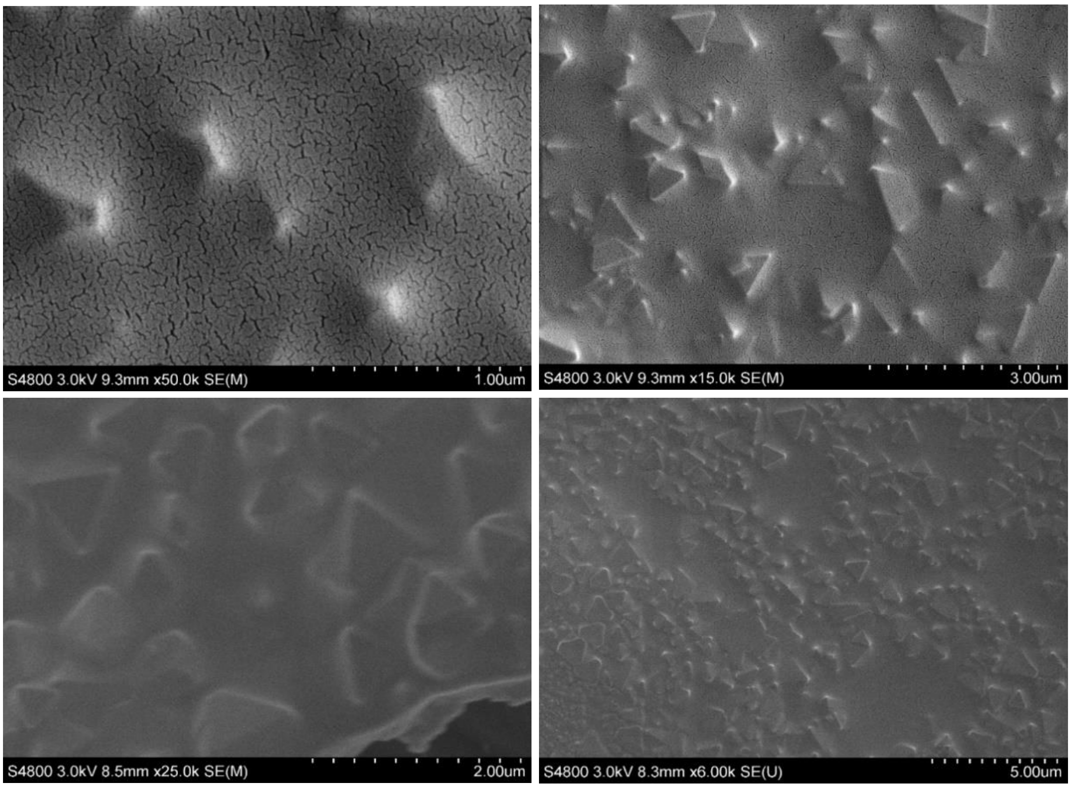

Figure S7. SEM images of continuous **CC3** film deposited on silicon wafers recorded at different resolutions. Octahedron-shaped crystals in the **CC3** film share the same crystal habit as the parent cage in its thermodynamically most stable crystalline form, **CC3**α[15]. Film synthesis conditions, reagent concentration: TFB 0.8 wt.% in DCM (30 mL), CHDA 0.8 wt.% in water (32 mL); reaction conditions: 24 hours at room temperature; dish diameter: 7.4 cm.

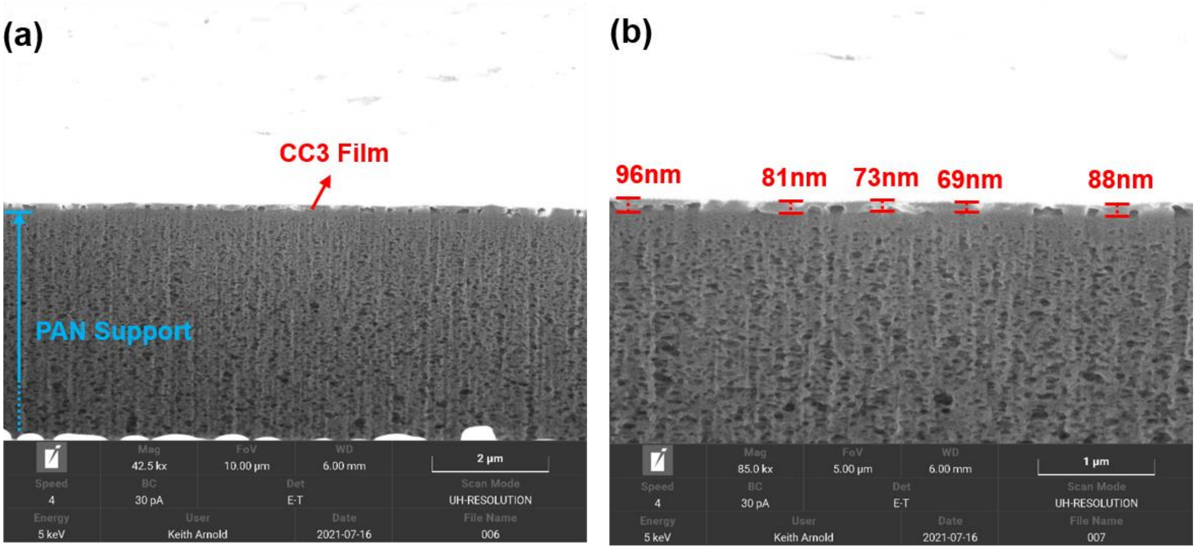

Figure S8. FIB-SEM images of **CC3**α-PAN-24hr showing its cross-sectional structure at different resolutions: (a) 42.5 k and (b) 85.0 k. The **CC3**α film thickness at different positions is included in part (b). Film synthesis conditions, reagent concentration: TFB 0.8 wt.% in DCM (30 mL), CHDA 0.8 wt.% in water (32 mL); reaction conditions: 24 hours at room temperature; dish diameter: 7.4 cm.

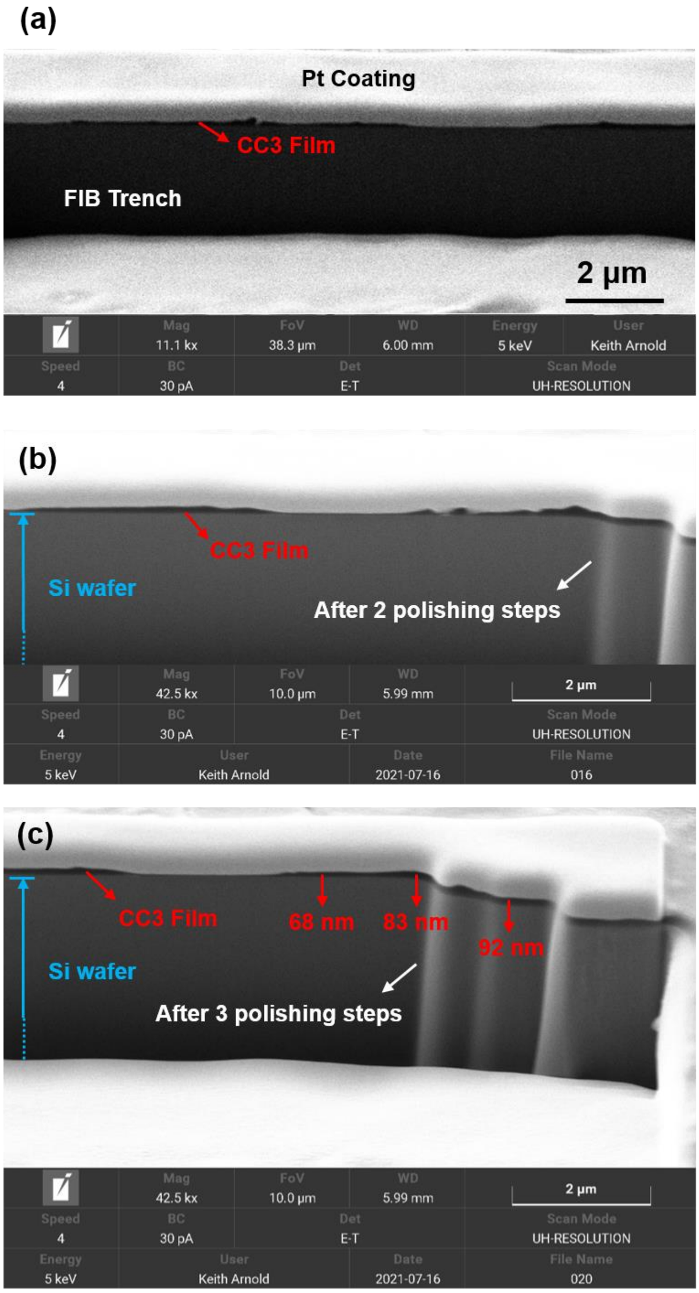

Figure S9. FIB-SEM images of **CC3**α-Si-24hr showing its cross-sectional structure: (a) FIB trench; (b) after two polishing steps; (c) after three polishing steps with the **CC3**α thickness at different positions included. The FIB was used for trenching and polishing (30kV and 1nA for trenching, and 30kV and 250pA for polishing), and the electron beam was used for imaging (5kV, 30pA, and 10kV, 60pA). Film synthesis conditions, reagent concentration: TFB 0.8 wt.% in DCM (30 mL), CHDA 0.8 wt.% in water (32 mL); reaction conditions: 24 hours at room temperature.

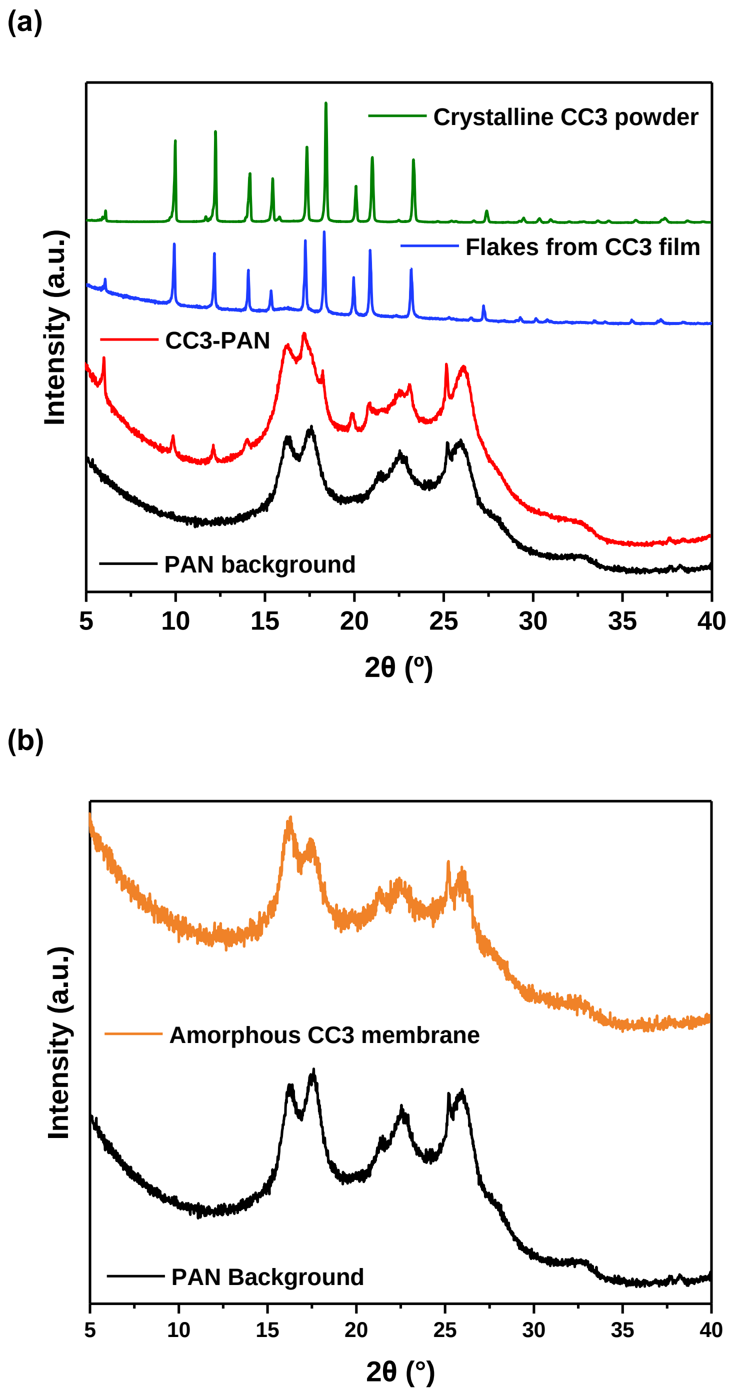

Figure S10. Structural characterisation of **CC3**α-PAN-24hr-0.8% and amorphous **CC3** membrane. PXRD pattern of, (a) blank PAN membrane (black), **CC3**α-PAN-24hr-0.8% (red), flakes collected from a free-standing **CC3** film that had been deposited on a glass substrate (blue), and crystalline **CC3**α reference (green); (b) blank PAN membrane (black) and amorphous **CC3** membrane (orange). PXRD patterns were collected in transmission mode with Cu-Kα radiation over the 2θ range of 5-40° using a step size of 0.013°. Data collection times, crystalline **CC3**α reference: 15 minutes; flakes from **CC3** film deposited on glass: 60 minutes; blank PAN and **CC3**α-PAN-24hr-0.8%: 300 minutes; amorphous **CC3** membrane: 60 minutes.

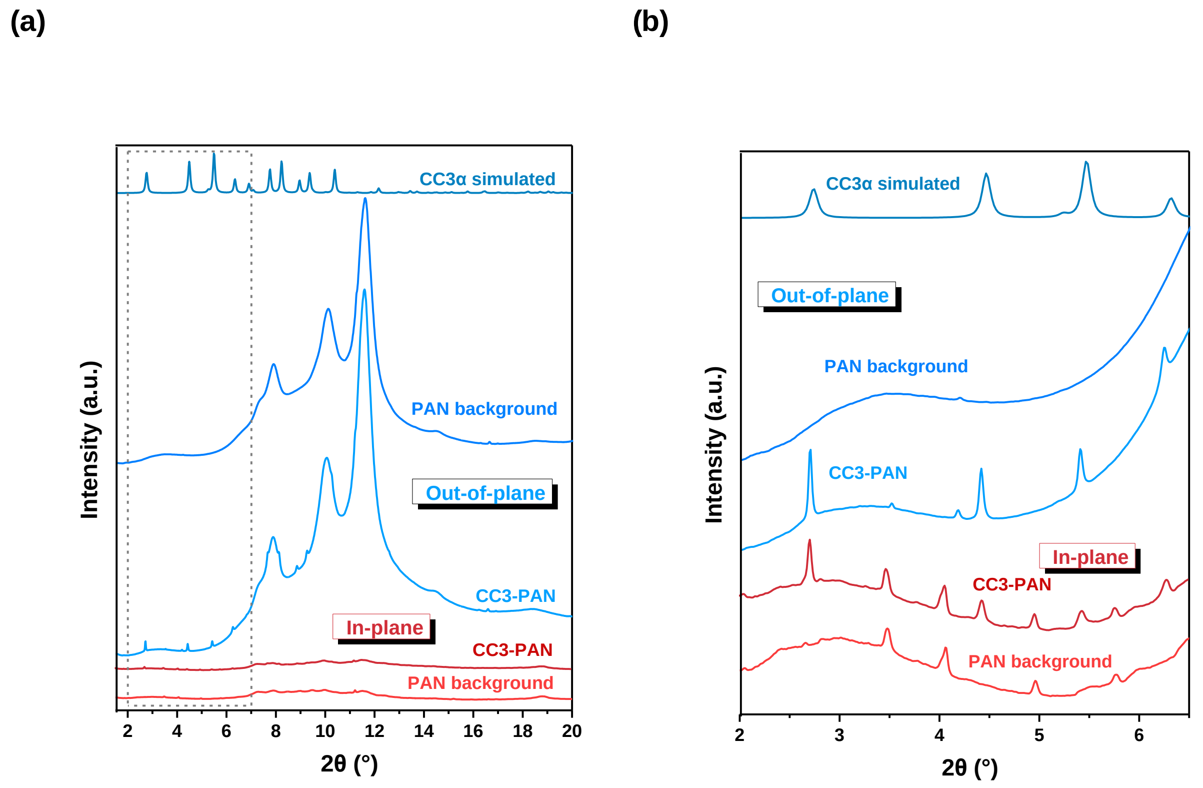

Figure S11. (a) In-plane and out-of-plane GIXRD patterns of **CC3**-PAN-24hr-0.8% membrane are shown below the simulated pattern for **CC3**α. A larger insert over the 2θ range 2 – 7° is shown in (b).

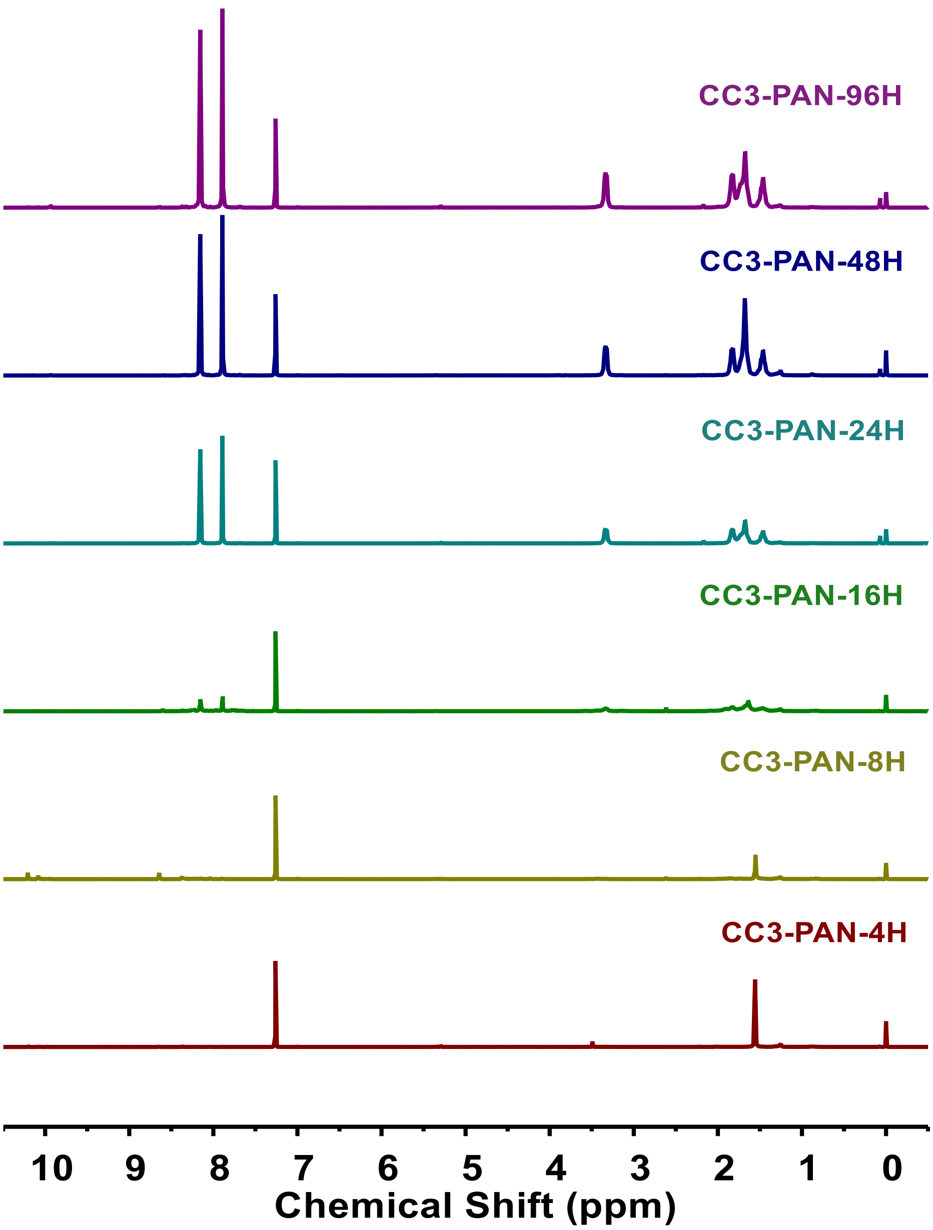

Figure S12. ^1H NMR ($CDCl_3$, 400 MHz) spectra for **CC3**α-PAN-Xhr-0.8% membranes fabricated using reaction times that ranged between 4–96 hours. Generic film synthesis conditions, reagent concentration: TFB 0.8 wt.% in DCM (30 mL), CHDA 0.8 wt.% in water (32 mL); reaction conditions: room temperature; dish diameter: 7.4 cm.

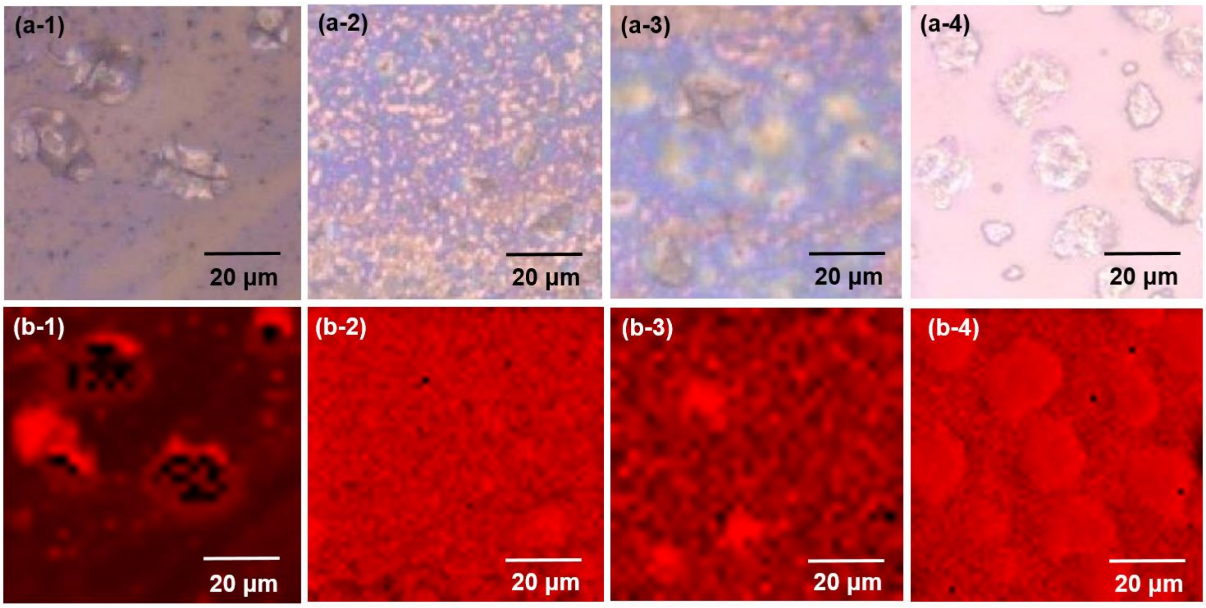

Figure S13. Raman microscope images (a) and maps (b) generated using an 80×80 μm^2 sized grid of **CC3**α-PAN-Xhr-0.8% that were fabricated over different reaction times, (1) 8 hours; (2) 16 hours; (3) 48 hours; (4) 96 hours, and deposited on glass substrates. The Raman spectra of **CC3**α and amorphous **CC3** were used as references, and the red regions represent matches with the Raman spectrum of **CC3**α. Generic film synthesis conditions, reagent concentration: TFB 0.8 wt.% in DCM (30 mL), CHDA 0.8 wt.% in water (32 mL); reaction conditions: room temperature; dish diameter: 7.4 cm. Scar bar: 20 µm. The Raman maps for **CC3**α-PAN-24hr-0.8% are shown in **Figure 2c** in the main text.

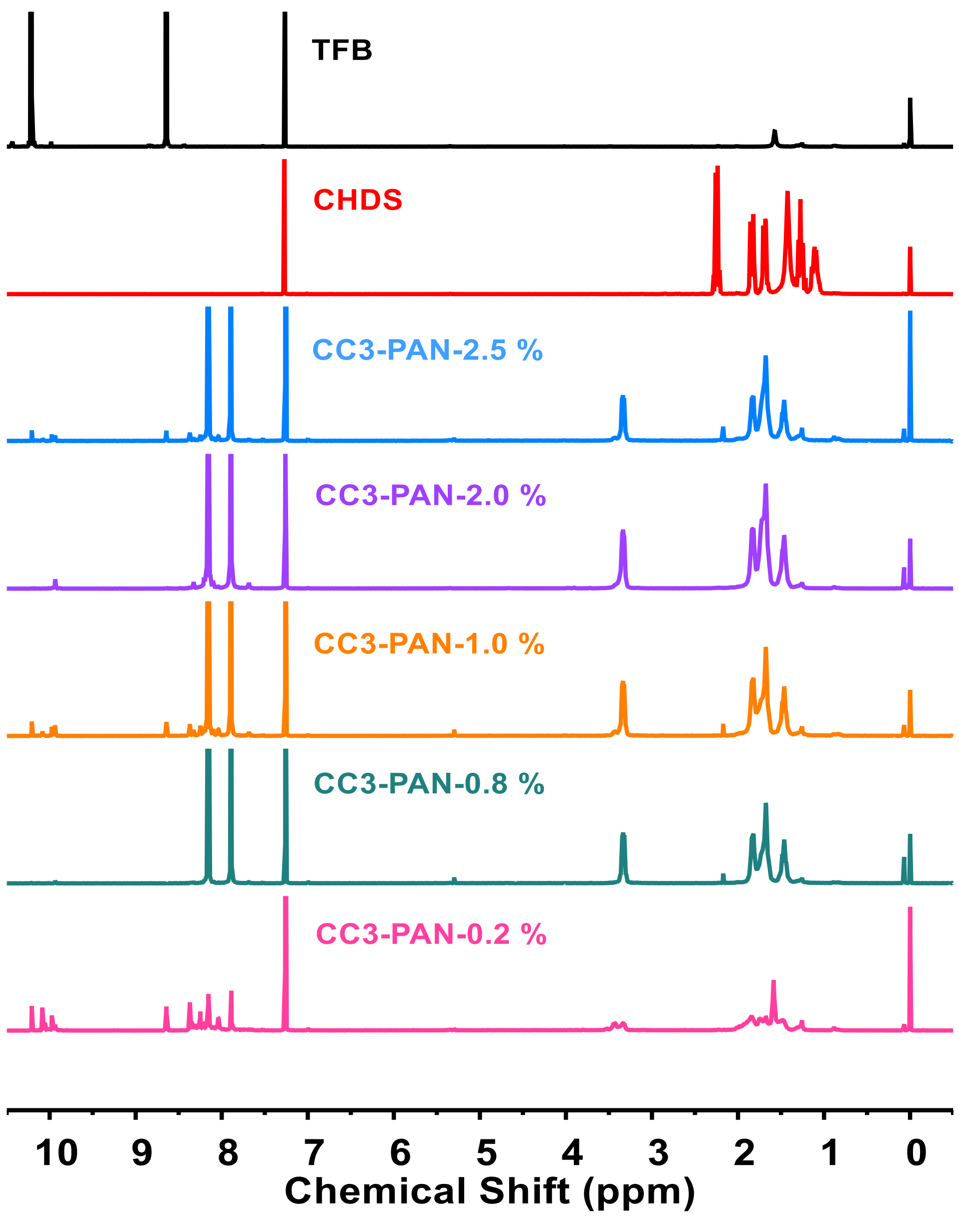

Figure S14. ^{1}H NMR ($CDCl_3$, 400 MHz) spectra for TFB, CHDA, and **CC3**α-PAN-24hr-Y% membranes fabricated using reagent concentrations that ranged between 0.2 wt.% to 2.5 wt.%. Generic film synthesis conditions, reagent concentration: TFB 0.2 wt.% to 2.5 wt.% in DCM (30 mL), CHDA 0.2 wt.% to 2.5 wt.% in water (32 mL); reaction conditions: 24 hours at room temperature; dish diameter: 7.4 cm.

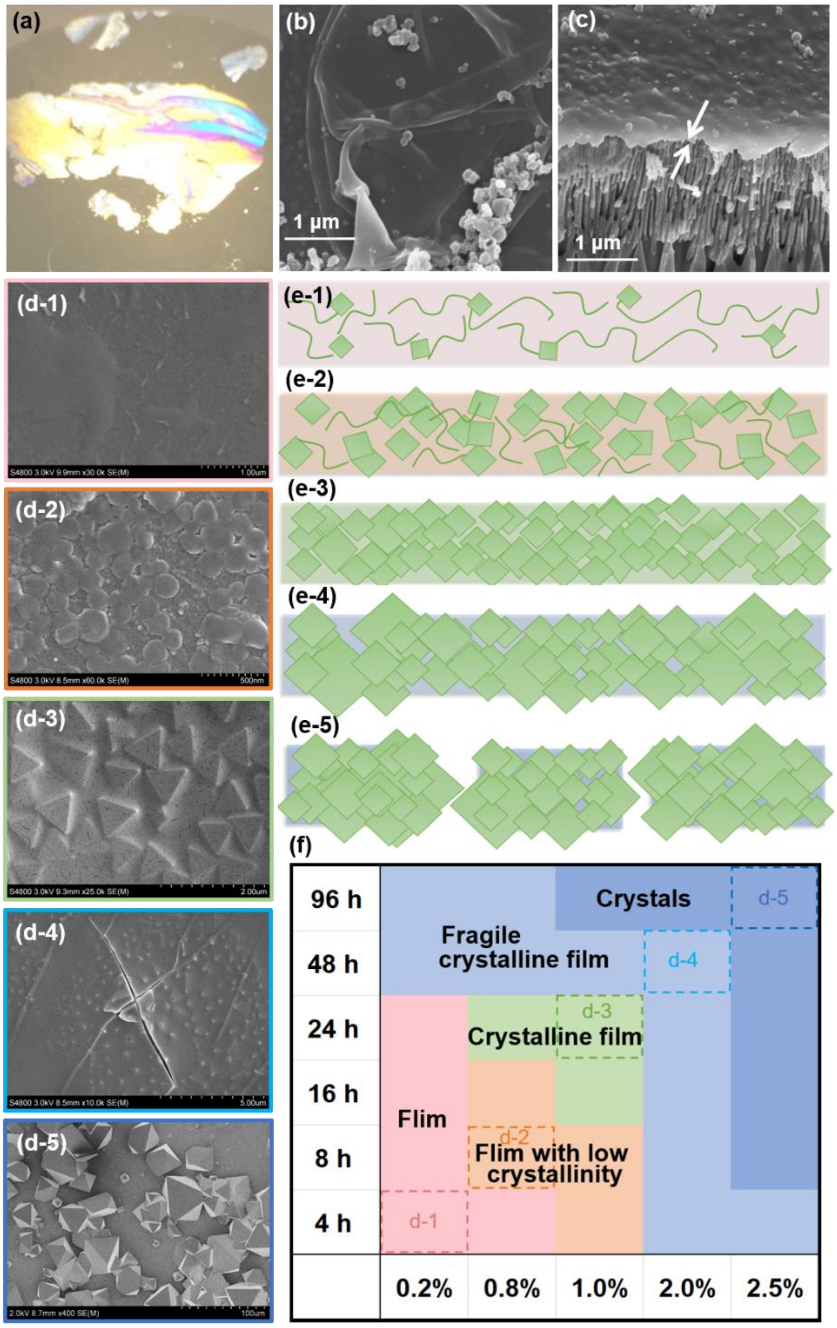

Figure S15. Morphology and proposed mechanism of **CC3** film formation. (a) Photography of a **CC3**α film deposited on an anodic aluminum oxide support, which was synthesised at a concentration of 0.2 wt.% for 24 hours at room temperature. (b, c) SEM images for this **CC3** film on an anodic aluminum oxide support showing its surface morphology and cross-sectional view. (d) SEM images of the **CC3** films that were synthesised using different reagent concentration and reaction times at room temperature and deposited on silicon wafers: (d-1) 0.2 wt.%, 4 hours (shown in pink in (f)); (d-2) 0.8 wt.%, 8 hours (orange in (f)); (d-3) 1.0 wt.%, 24 hours (green in (f)); (d-4) 2.0 wt.%, 48 hours (cyan in (f)); (d-5) 2.5 wt.%, 96 hours (blue in (f)). (e1-e5) Graphical representation of proposed mechanism, where green squares and lines represent **CC3** crystals and non-crystalline components, respectively. (f) Summary of crystalline **CC3** film formation using different reaction conditions, which is based on a combination of NMR, XRD, Raman, and SEM data. The pink region represents continuous amorphous film; the orange region represents film with low crystallinity; the green region represents the optimal synthesis conditions; cyan and blue regions represent highly crystalline and less continuous (more fragile) samples.

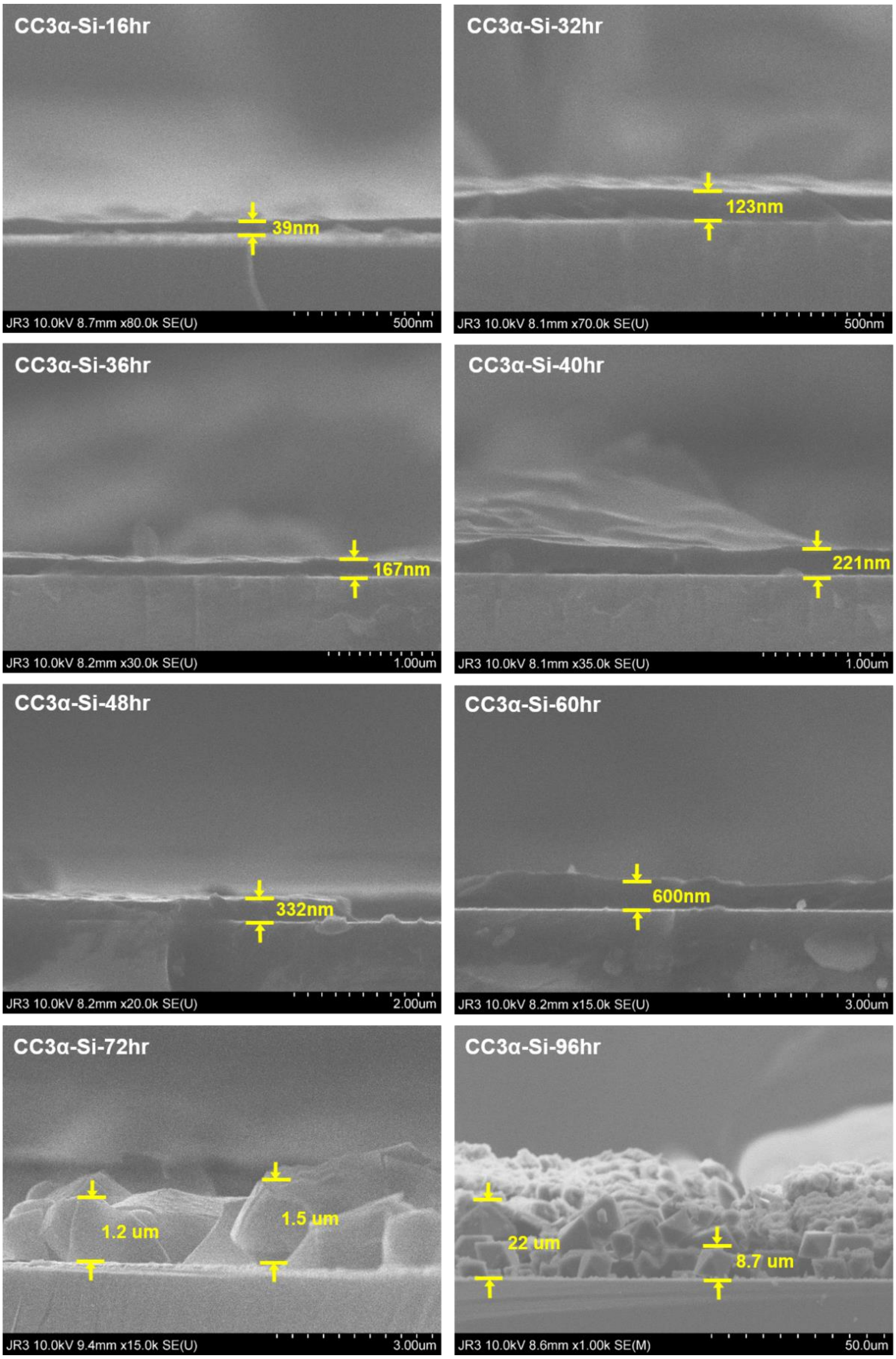

Figure S16. SEM images of **CC3**α films prepared at different reaction times (**CC3**α-Si-0.8%-Xhr, where X = 16-96 hr) and then deposited on silicon wafers. The **CC3**α film thickness is shown in the images. Generic film synthesis conditions, reagent concentration: TFB 0.8 wt.% in DCM (30 mL), CHDA 0.8 wt.% in water (32 mL); reaction conditions: room temperature. Note, the **CC3**-Si-0.8%-4hr was too thin to be observed under the SEM. Instead, AFM height images were obtained to determine the **CC3** film thickness.

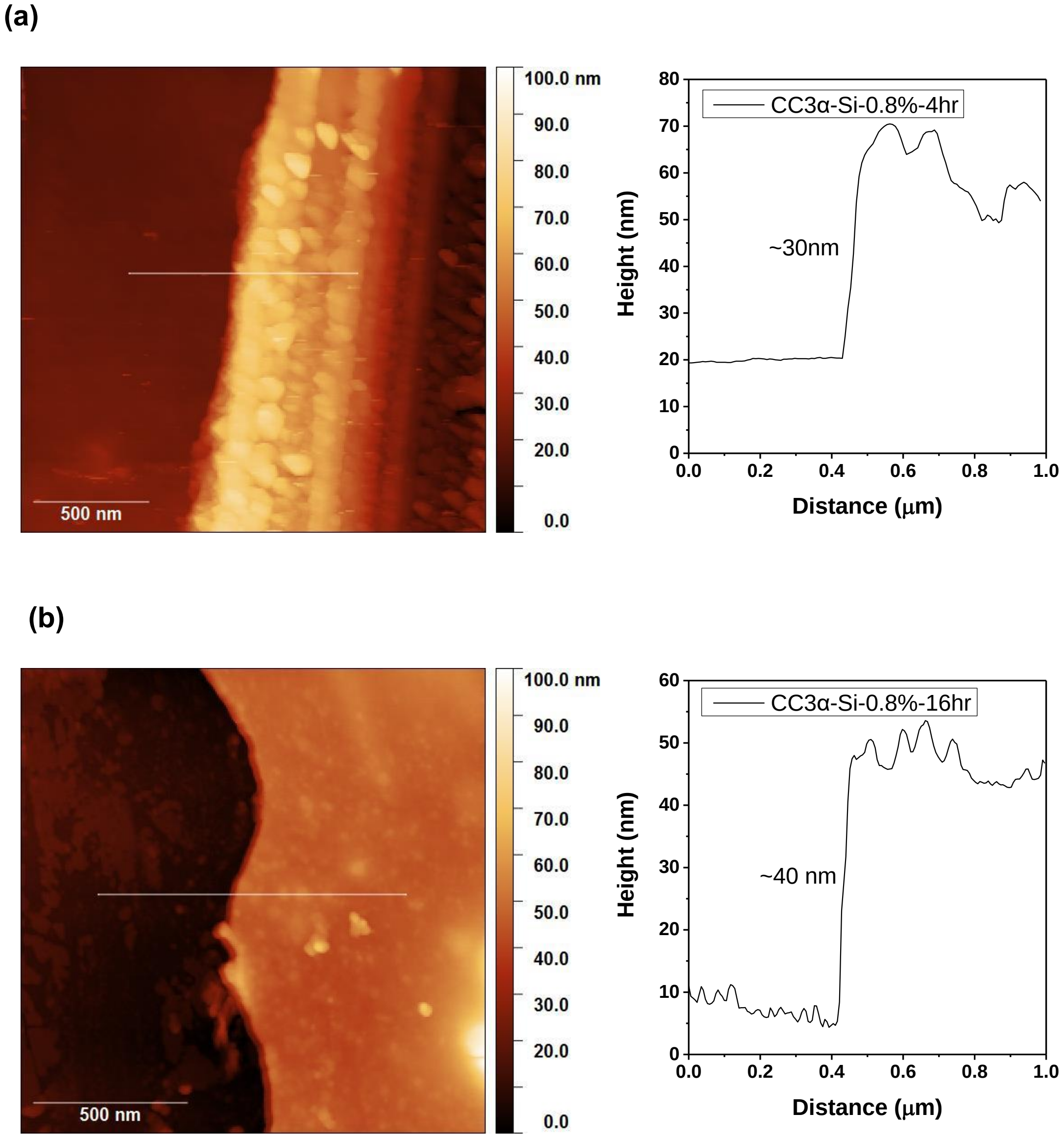

Figure S17. AFM height image and the height profile of **CC3**α films prepared under different conditions transferred onto a silicon (Si) wafer. **CC3**α-Si-0.8%-Xhr samples were fabricated using reaction times: (a) 4 hours; (b) 16 hours. Generic film synthesis conditions and reagent concentrations were as follows: TFB 0.8 wt.% in DCM (30 mL), CHDA 0.8 wt.% in water (32 mL); reaction conditions: room temperature. Note that the **CC3**α-Si-0.8% after 48 hours and 96 hours reaction were too fragile/loose for AFM measurements. Instead, cross-sectional SEM images were recorded to determine the **CC3** film thickness.

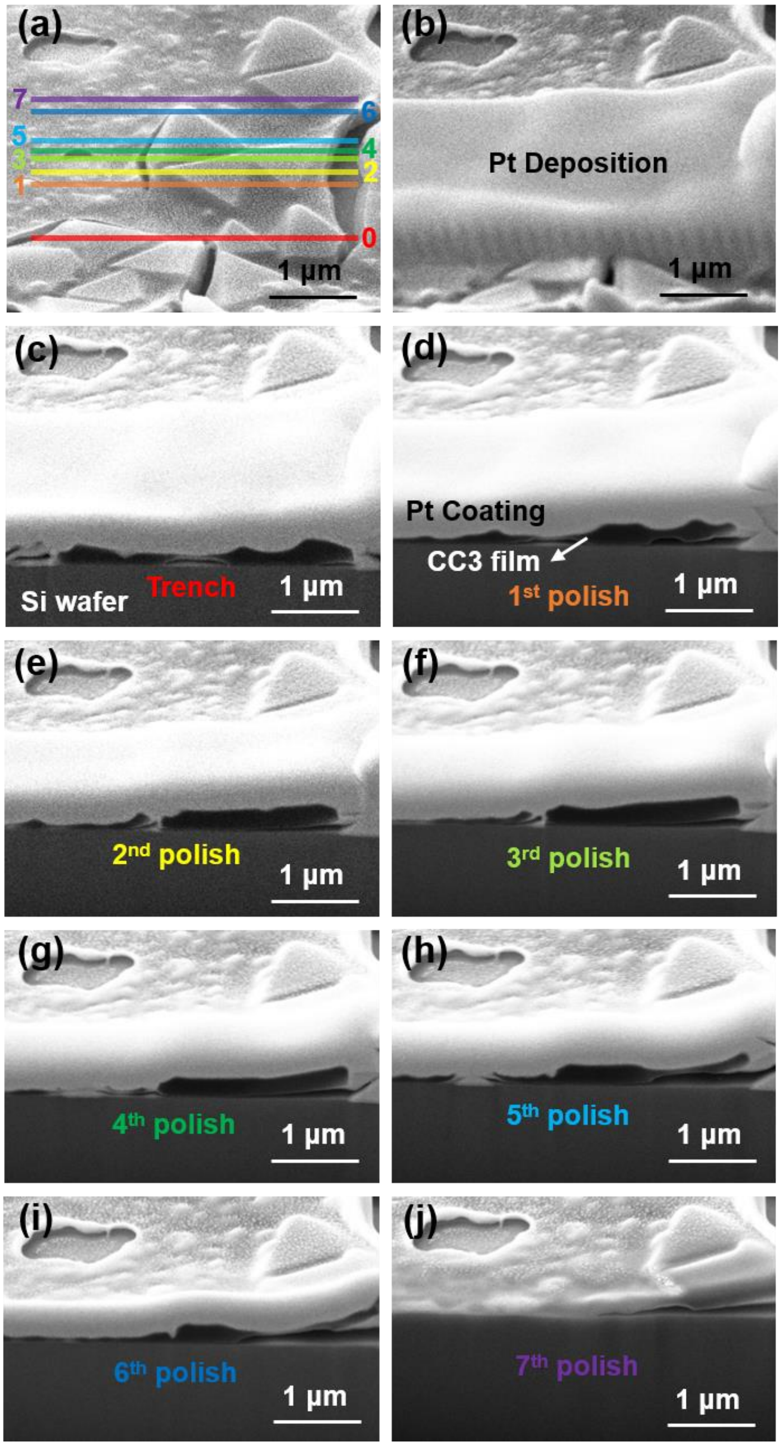

Figure S18. FIB-SEM images of **CC3**α-Si-32hr showing its cross-sectional structure during step-by-step trenching and polishing: (a) Surface marked with 0-7 polishing step tracks; (b) Pt-coated surface area; cross-section after FIB treatment: (c) after trenching (0 polishing step); (d) after 1 polishing step; (e) after 2 polishing steps; (f) after 3 polishing steps; (g) after 4 polishing steps; (h) after 5 polishing steps; (i) after 6 polishing steps; (j) after 7 polishing steps. The FIB was used for trenching and polishing (30kV and 1nA for trenching, and 30kV and 250pA for polishing), and the electron beam was used for imaging (5kV, 30pA, and 10kV, 60pA). Film synthesis conditions, reagent concentration: TFB 0.8 wt.% in DCM (30 mL), CHDA 0.8 wt.% in water (32 mL); reaction conditions: 32 hours at room temperature.

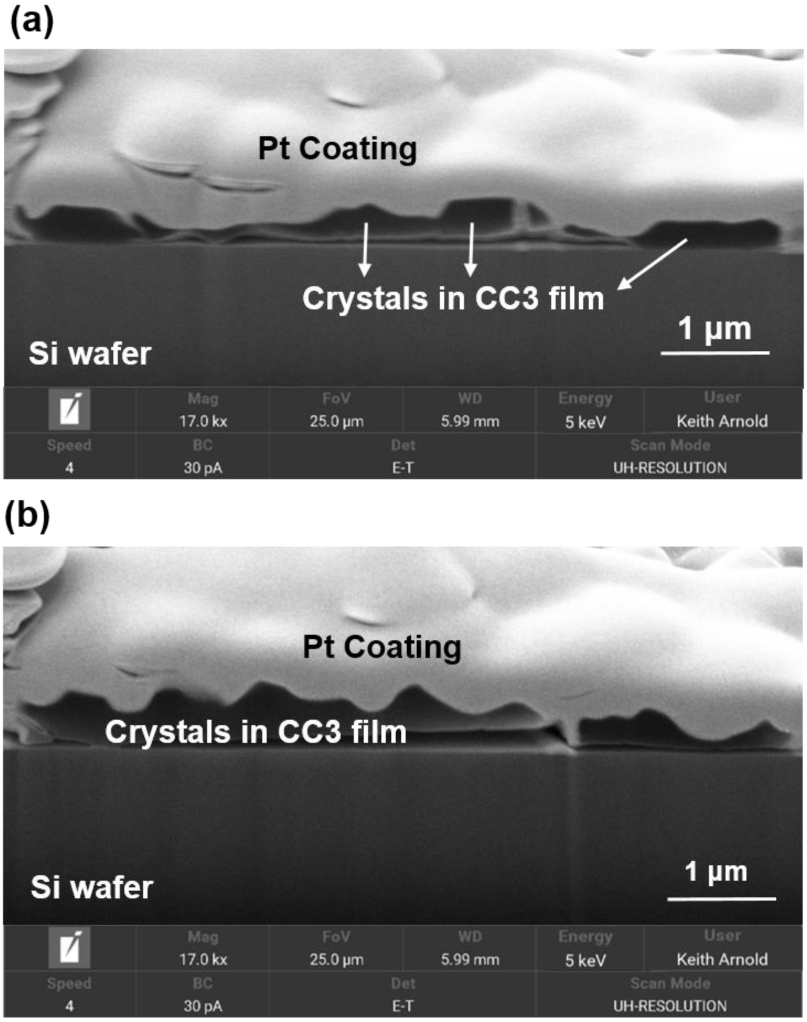

Figure S19. FIB-SEM images of **CC3**α-Si-48hr (a) showing its cross-sectional structure after FIB trenching, and (b) after polishing. The FIB was used for trenching and polishing (30kV and 1nA for trenching, and 30kV and 250pA for polishing), and the electron beam was used for imaging (5kV, 30pA, and 10kV, 60pA). Film synthesis conditions, reagent concentration: TFB 0.8 wt.% in DCM (30 mL), CHDA 0.8 wt.% in water (32 mL); reaction conditions: 48 hours at room temperature.

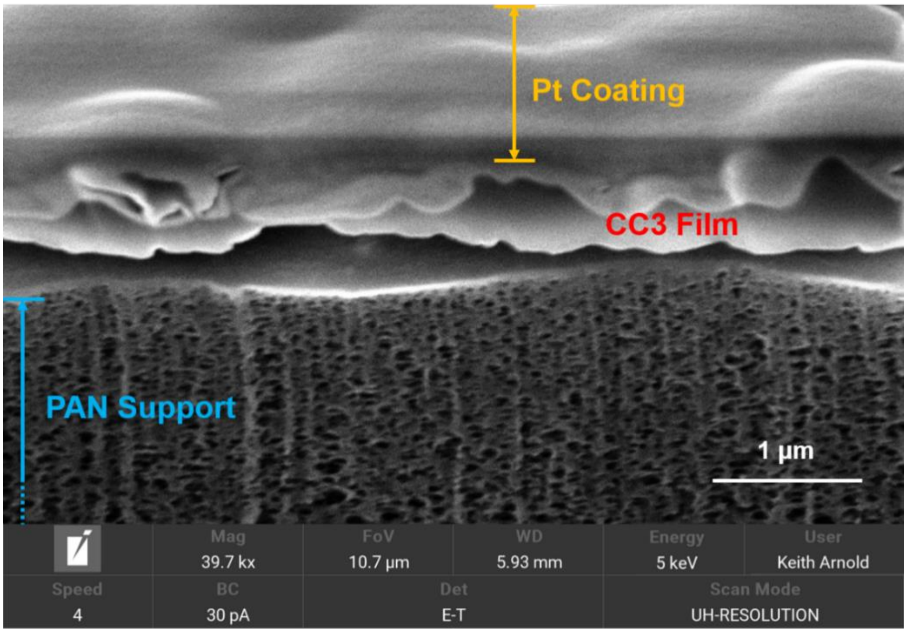

Figure S20. FIB-SEM images of **CC3**α-PAN-48hr showing its cross-sectional structure, using the FIB for trenching and polishing (30kV and 1nA for trenching, and 30kV and 250pA for polishing) and the electron beam for imaging (5kV, 30pA, and 10kV, 60pA). Film synthesis conditions, reagent concentration: TFB 0.8 wt.% in DCM (30 mL), CHDA 0.8 wt.% in water (32 mL); reaction conditions: 48 hours at room temperature; dish diameter: 7.4 cm.

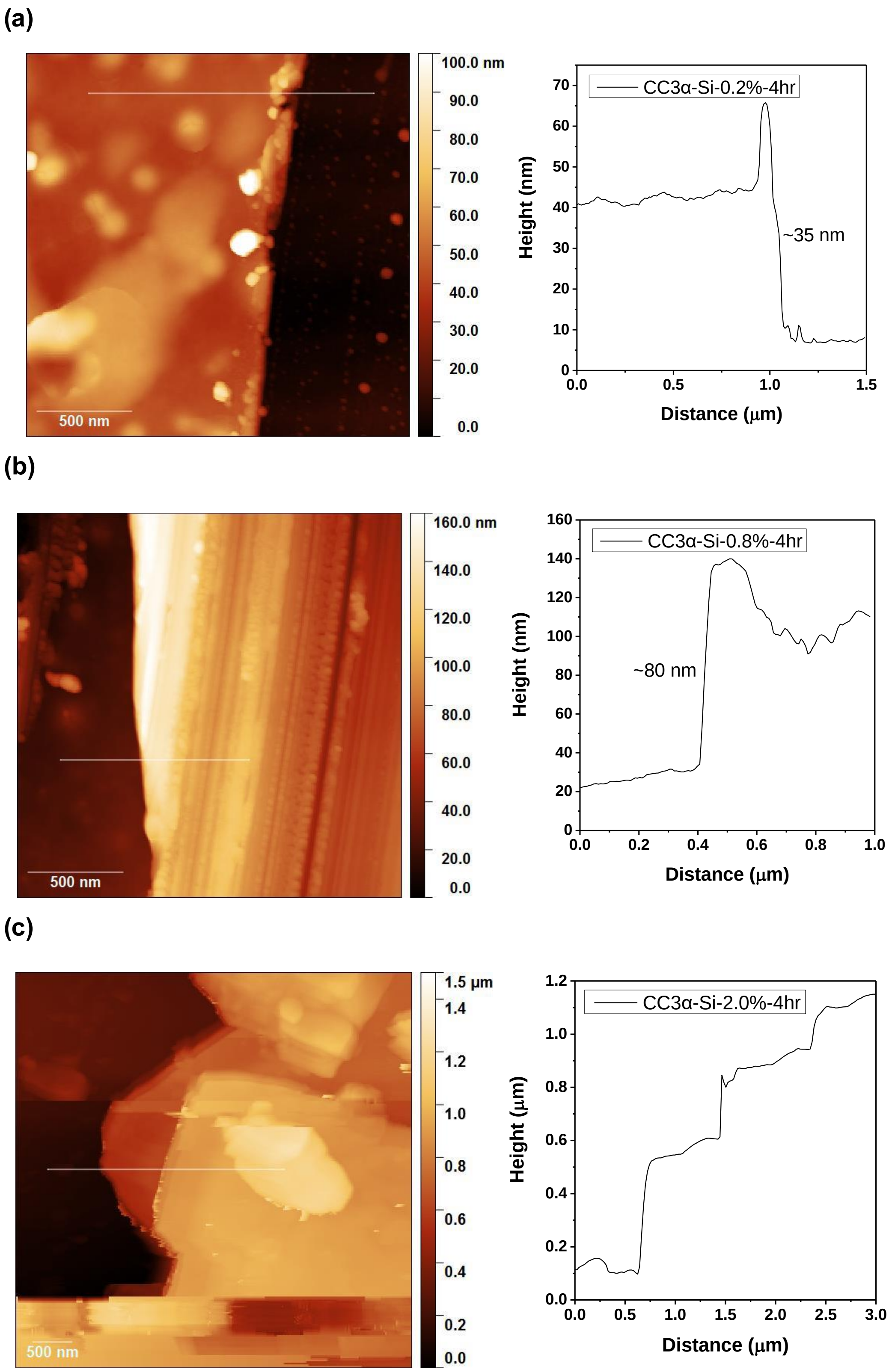

Figure S21. AFM height image and the height profile of **CC3α** films prepared under different conditions transferred onto a silicon (Si) wafer. **CC3α**-Si-X%-4hr samples were fabricated using reaction concentrations: (a) 0.2 wt.%; (b) 0.8 wt.%; (c) 2.0 wt.%. Reaction conditions: 4 hours at room temperature.

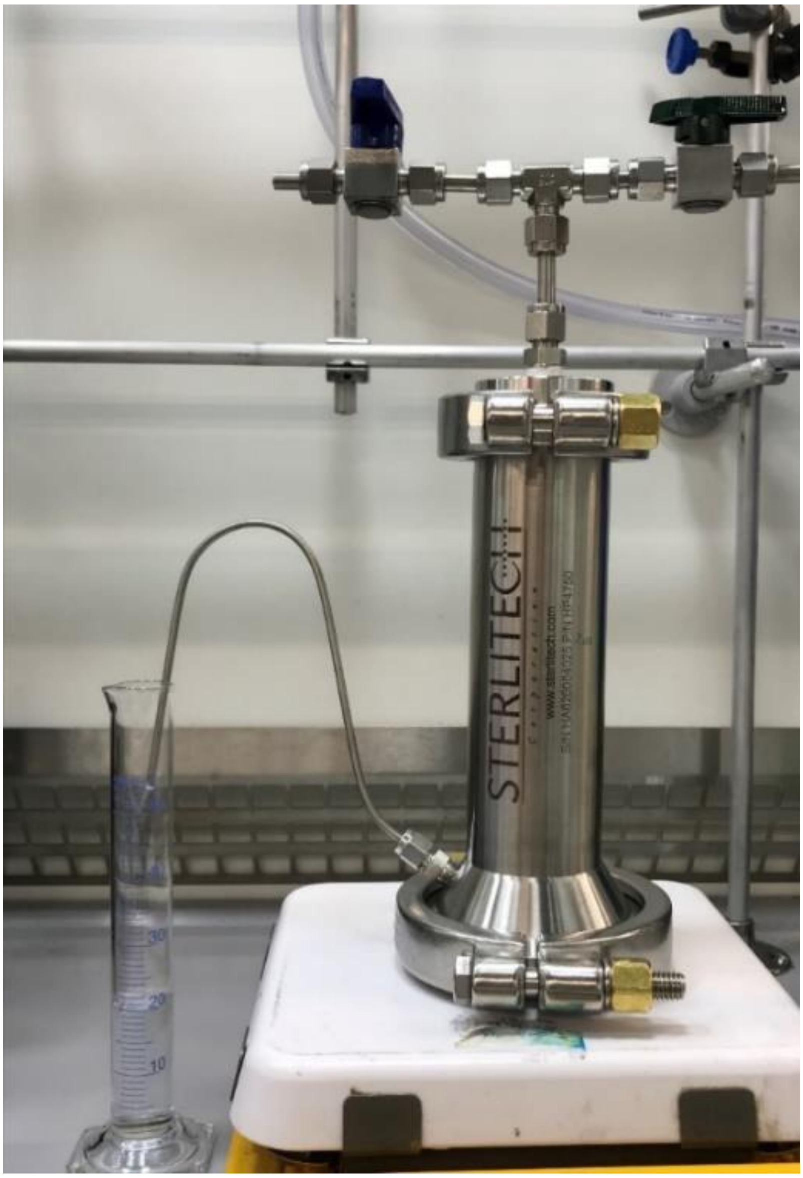

Figure S22. To evaluate the separation performance of the **CC3**-PAN membranes, the permeance and selectivity measurements were carried out using a commercial bench-scale dead-end stirred-cell filtration unit (Sterlitech® HP4750 stirred cell) that was kept under 10 bar upstream nitrogen pressure at room temperature. The agitation speed was kept at 400 rpm during the measurements. The membrane was cut into a coupon with a diameter of 4.7 cm to fix at the bottom of the cell.

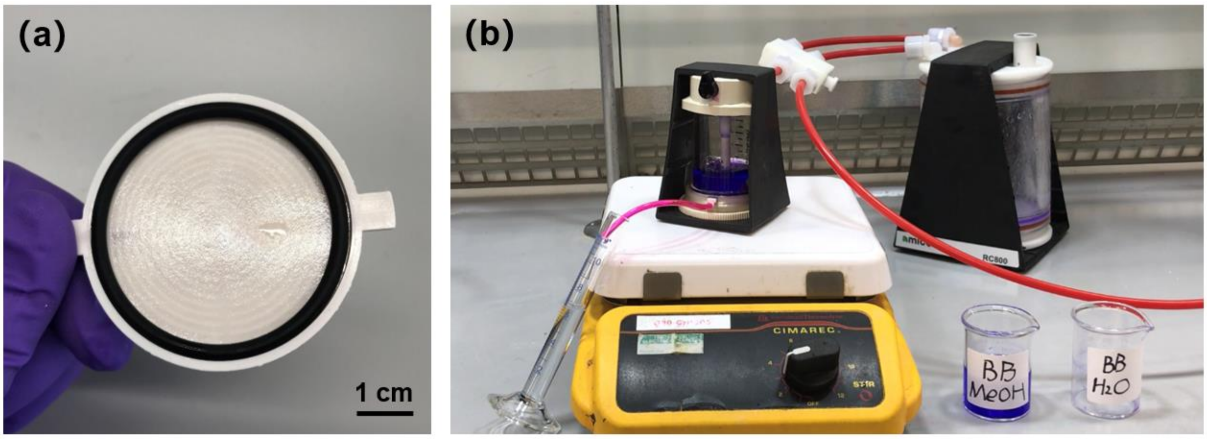

Figure S23. To visualise the filtration process in real-time, a commercial bench-scale 50 mL Merck Millipore Amicon® dead-end stirred cell which is connected to a 800 mL Merck Millipore Amicon® RC800 reservoir was used. (a) Photograph showing a **CC3**α-PAN membrane sample fixed on the support at the base of the cell that was sealed using an O-ring. The membrane was cut into a circle coupon with a diameter of 42 mm to fix at the bottom of the cell. Scale bar: 1cm. (b) Photograph showing the filtration set-up and connections of the dead-end filtration cell that was kept under a 3 bar nitrogen atmosphere. Feed solutions that contained 20 ppm BB in MeOH and 20 ppm BB in water were prepared and used for the filtration experiments.

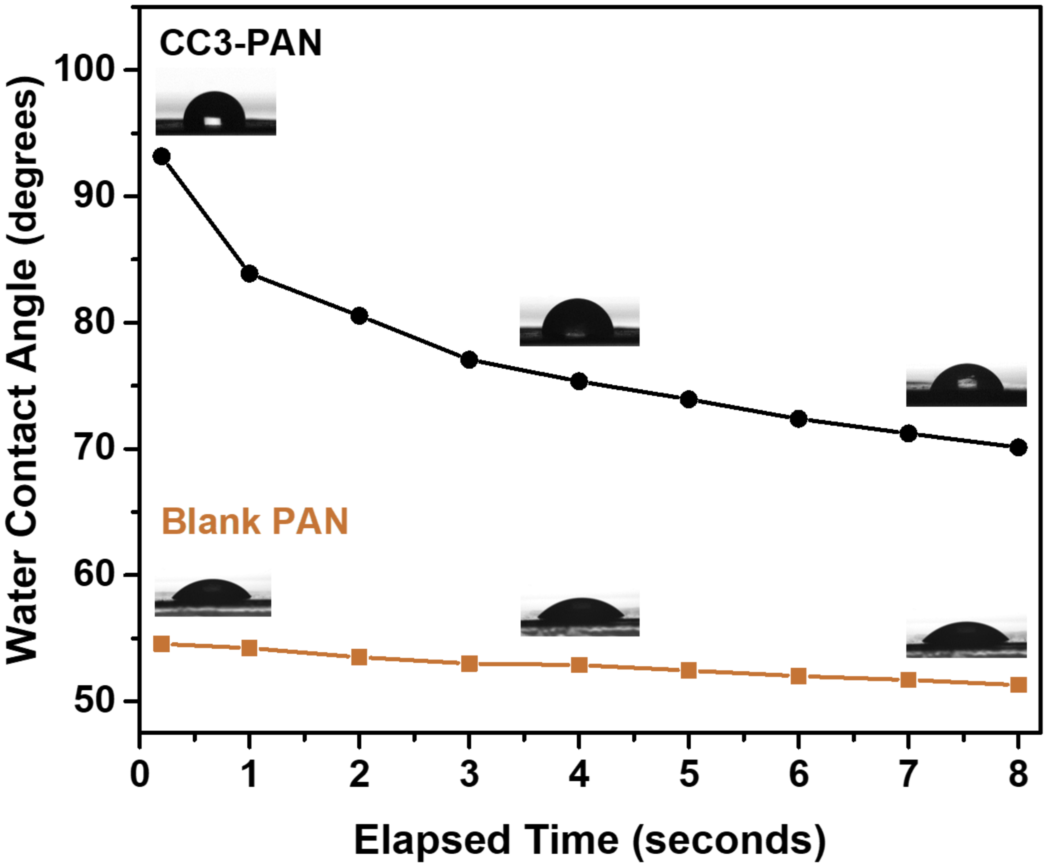

Figure S24. Water contact angles for **CC3**α-PAN membrane. Drop phase: water; surrounding phase: air; drop type: sessile drop; drop volume: 4.15 ± 0.5 μL; fitting method: Young–Laplace equation.

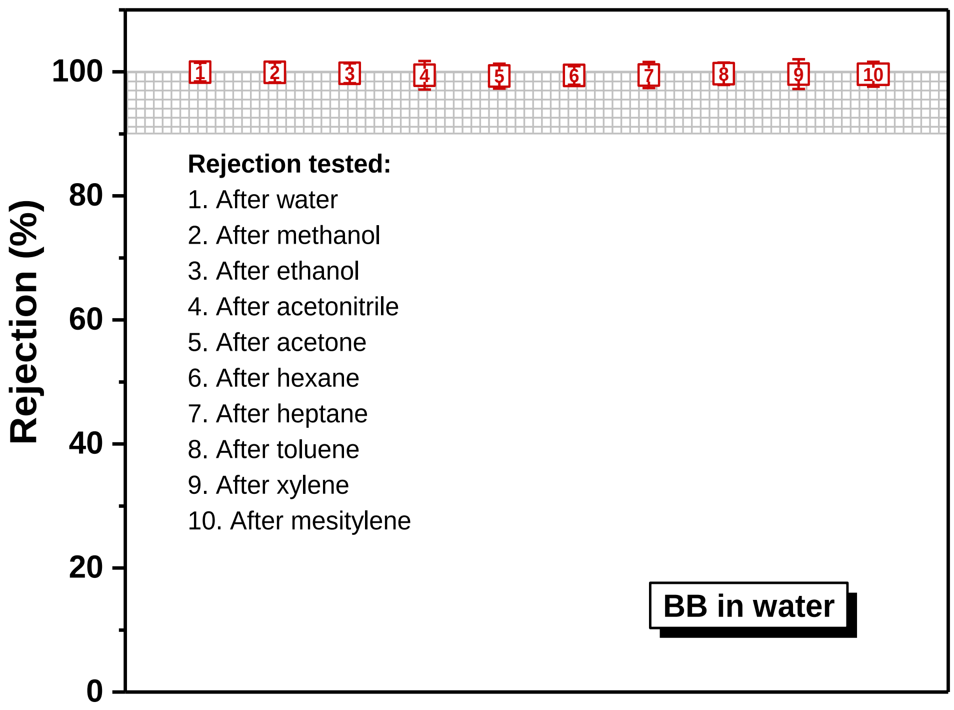

Figure S25. Dye rejection performance of **CC3α**-PAN, determined after measuring the permeance rates of organic solvents. A 20 ppm BB water feedstock was used to check the membrane stability of **CC3α**-PAN after being permeated 100 mL of different organic solvents. The upstream nitrogen pressure was kept at 10 bar during the measurement and the stirrer agitation speed was 400 rpm. All the error bars depict the standard deviations of the data from at least three independent membranes.

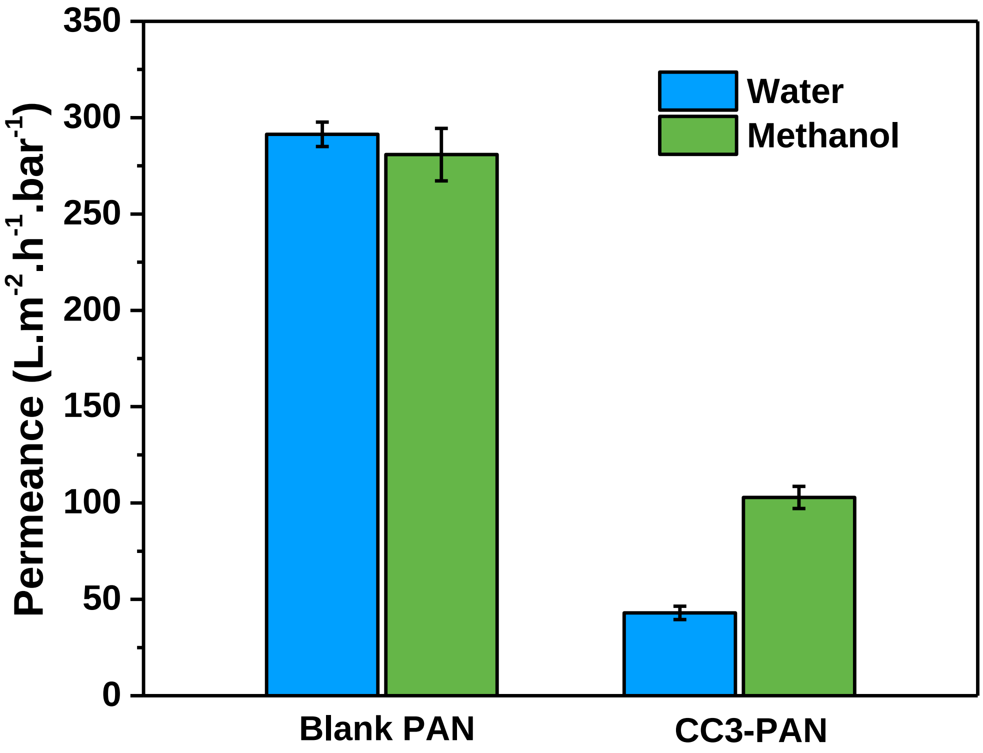

Figure S26. Water and MeOH permeance of a blank PAN membrane and the **CC3-**PAN membranes. The permeance values were measured under the same conditions with the upstream nitrogen pressure kept at 10 bar during the measurement, and the stirrer agitation speed was set at 400 rpm. The error bars denote the standard deviations for measurements from at least three independent membranes.

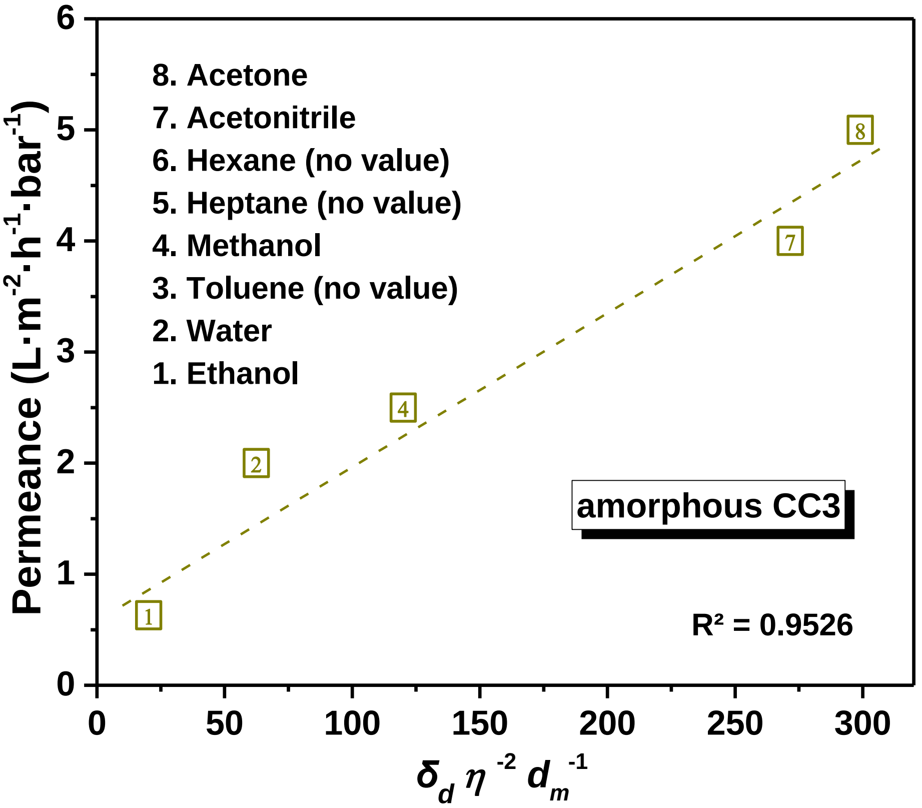

Figure S27. Plots showing pure solvent permeances *versus* their combined solvent properties (viscosity, molar diameter, and solubility parameter) for amorphous **CC3** membrane. The cell was kept at room temperature under 10 bar upstream pressure in a nitrogen atmosphere, with 400 rpm magnetic stirring.

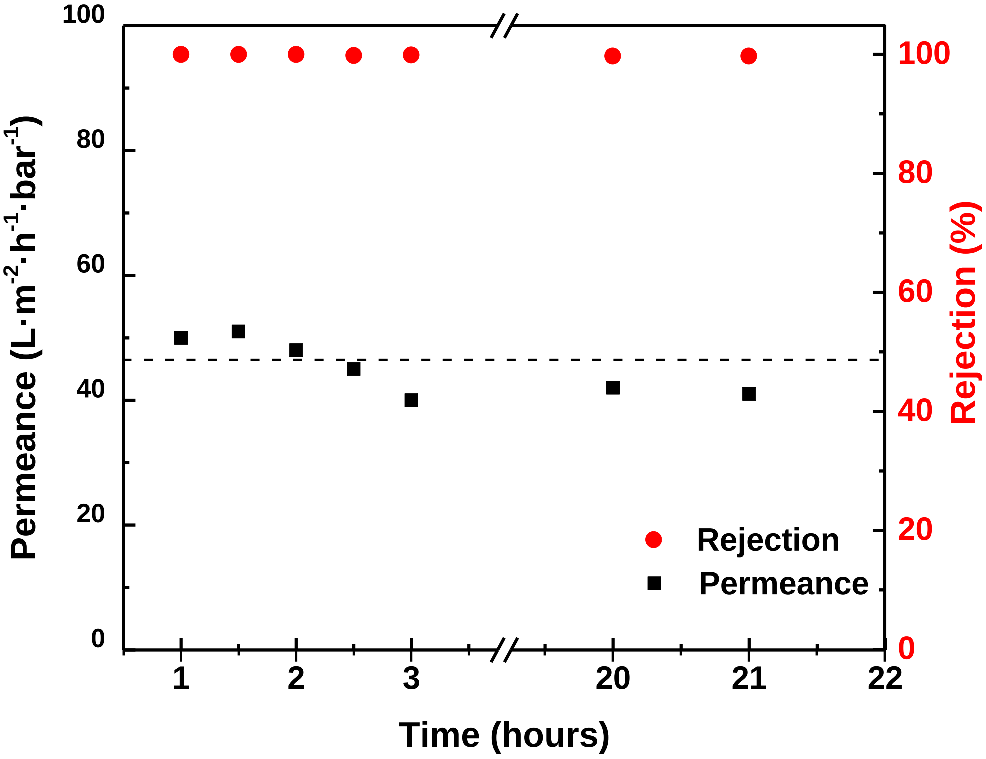

Figure S28. Membrane selectivity and stability of **CC3α**-PAN in water. The plot shows water permeance and dye rejection of RB from a 20 ppm feedstock for the **CC3α**-PAN membrane over ~ 20 hours in a 50 mL transparent Merck Millipore Amicon® dead-end stirred cell which is connected to an 800 mL Merck Millipore Amicon® RC800 reservoir. The upstream nitrogen pressure was kept at 1 bar during the measurement and the stirrer agitation speed was 400 rpm.

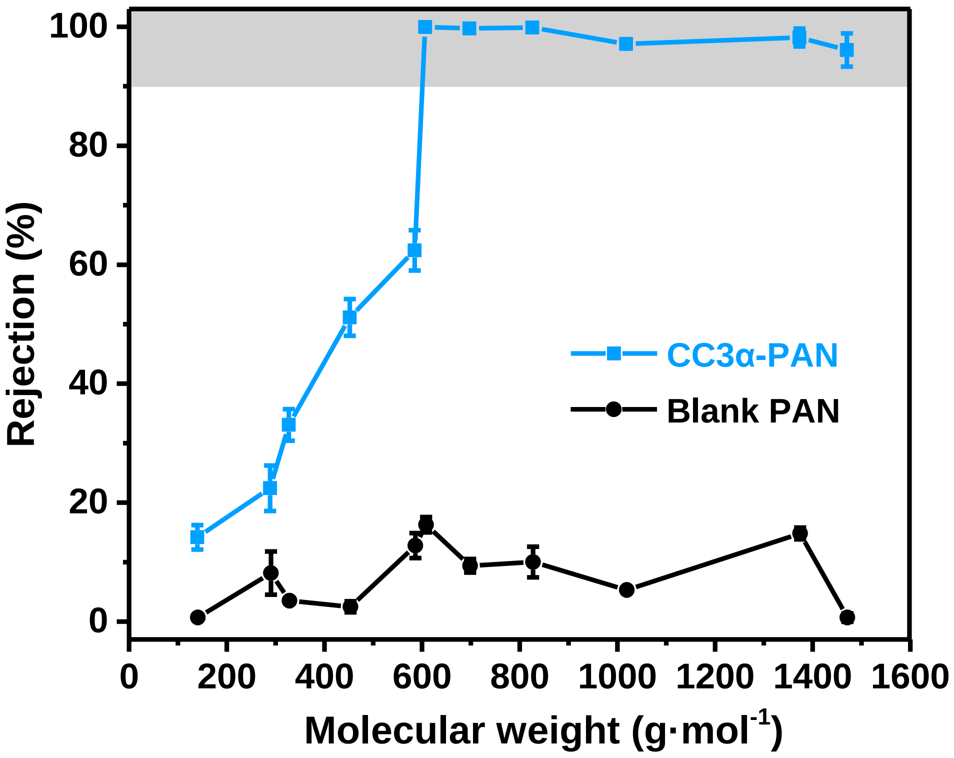

Figure S29. Dye rejection versus molecular weight cut-off curve for **CC3**α-PAN and blank PAN membrane in water containing 20 ppm dye solutes. The upstream nitrogen pressure was kept at 10 bar during the measurement, and the stirrer agitation speed was 400 rpm. The error bars denote the standard deviations for measurements from at least three independent membranes.

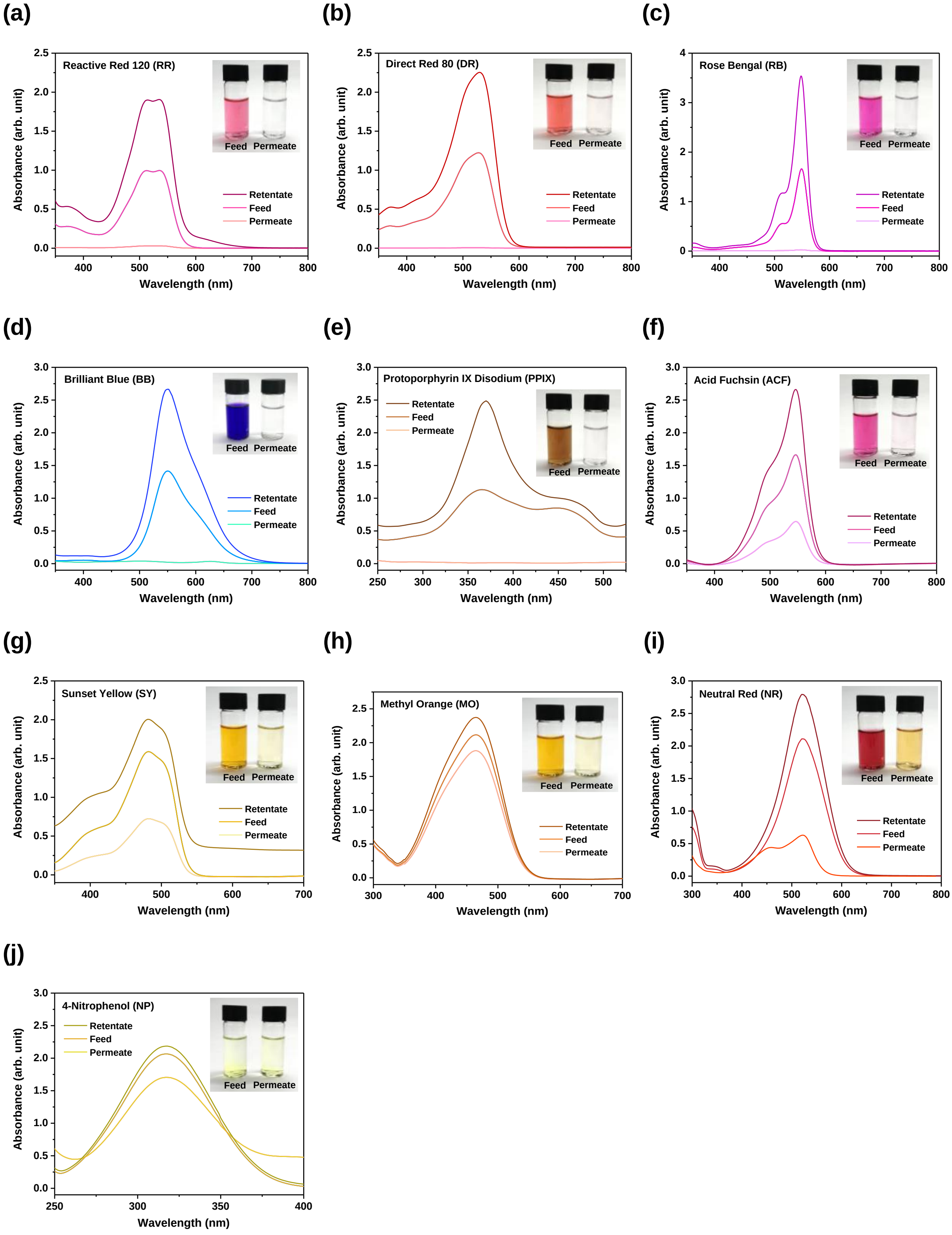

Figure S30. UV absorption spectra of dyes in water before and after selectivity tests performed with **CC3**α-PAN. Inserts show photographs of the feed and the permeate. Dye rejection was calculated using the intensity of the maximum absorption peak in the permeate and the feed and *Eq*. 3 in the Methods section. Mass balance was calculated using the maximum absorption peaks in the values of the feed, permeate, and retentate with *Eq*. 4 in the Methods section. Note, NR is a pH indicator, changing from red to yellow between pH 6.8 and 8.0; as the NR permeate was orange in colour the dye concentration was calculated using both yellow and red peaks in the UV absorption spectrum. The absorption spectra of CR are presented in **Figure 3f** in the main text.

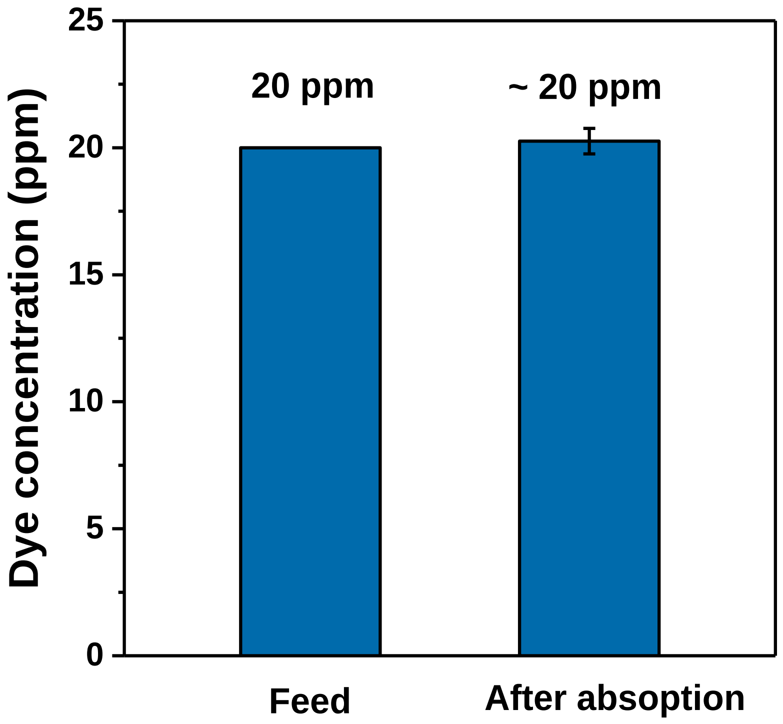

Figure S31. Dye absorption test. The concentration of the feed solution and the solution with powder crystals of **CC3**α (100 mg) immersed for one week, measured by UV absorption data and calculated by running five samples with known concentration as the standard line. The dye concentration remained at ~ 20 ppm after the absorption test was run continually for one week, showing there was no obvious absorption on the membrane surface. The error bar depicts the standard deviations of the data from at least three independent membranes.

(a)

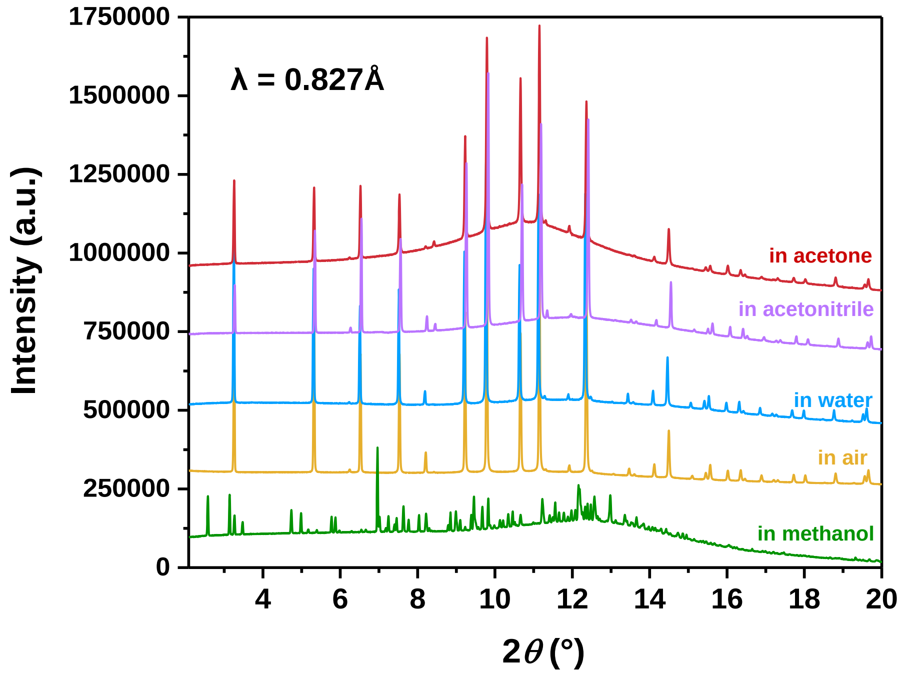

(b)

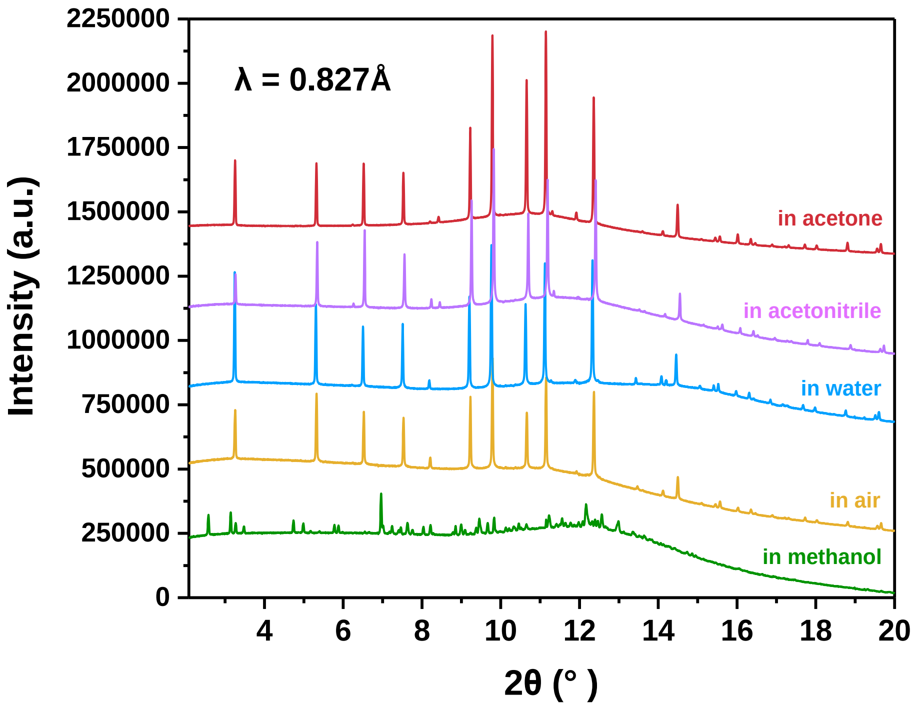

Figure S32. PXRD patterns recorded on, (a) bulk grown powders of as-synthesised **CC3**α crystals loaded into glass capillaries and then suspended in organic solvents; (b) **CC3**α film that was scaped of a glass substrate loaded in glass capillaries and then dispersed in organic solvents. TFB 0.8 wt.% in DCM (30 mL), CHDA 0.8 wt.% in water (32 mL); reaction conditions: 24 hours at room temperature; dish diameter: 7.4 cm. Wavelength: λ = 0.827 Å.

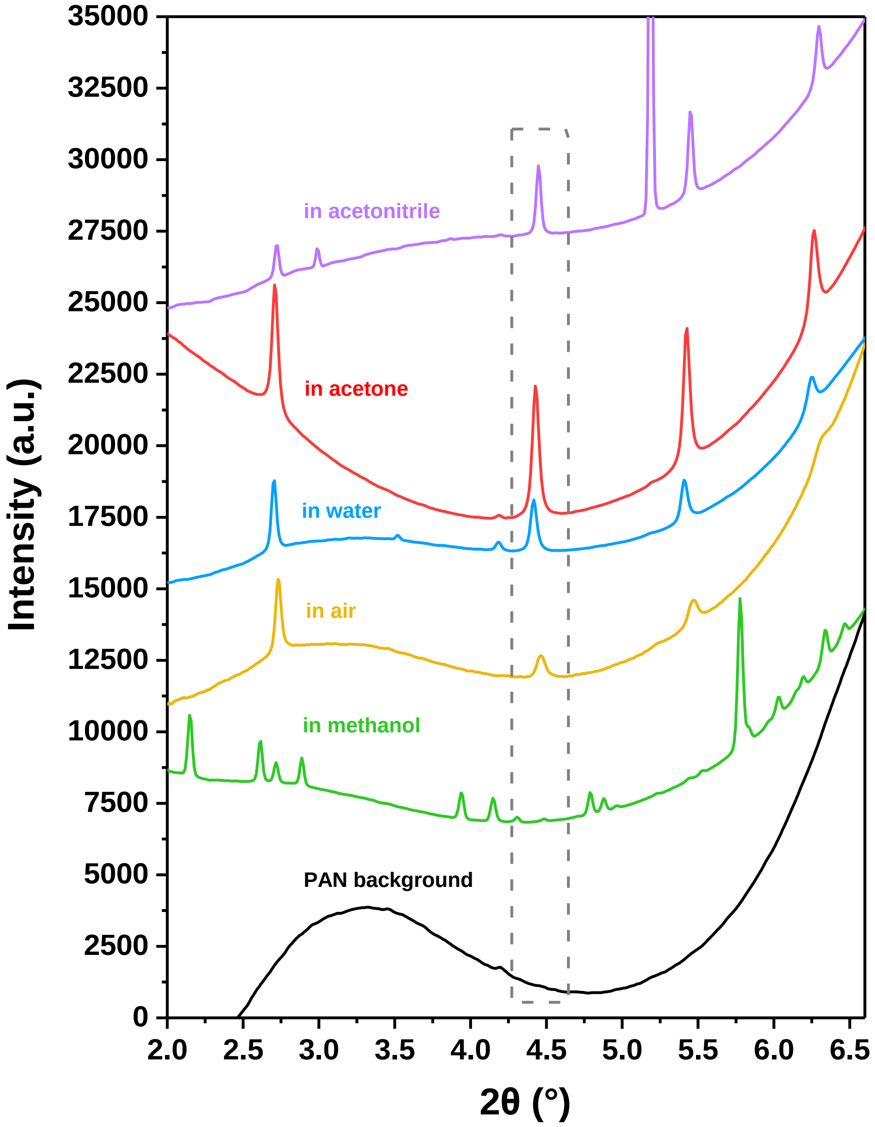

Figure S33. Out-of-plane GIXRD diffraction patterns (λ = 0.689 Å) that were recorded after coating the surface of **CC3**α-PAN-24hr-0.8% with different organic solvents. The solvent layer was covered with Mylar film during the measurements.

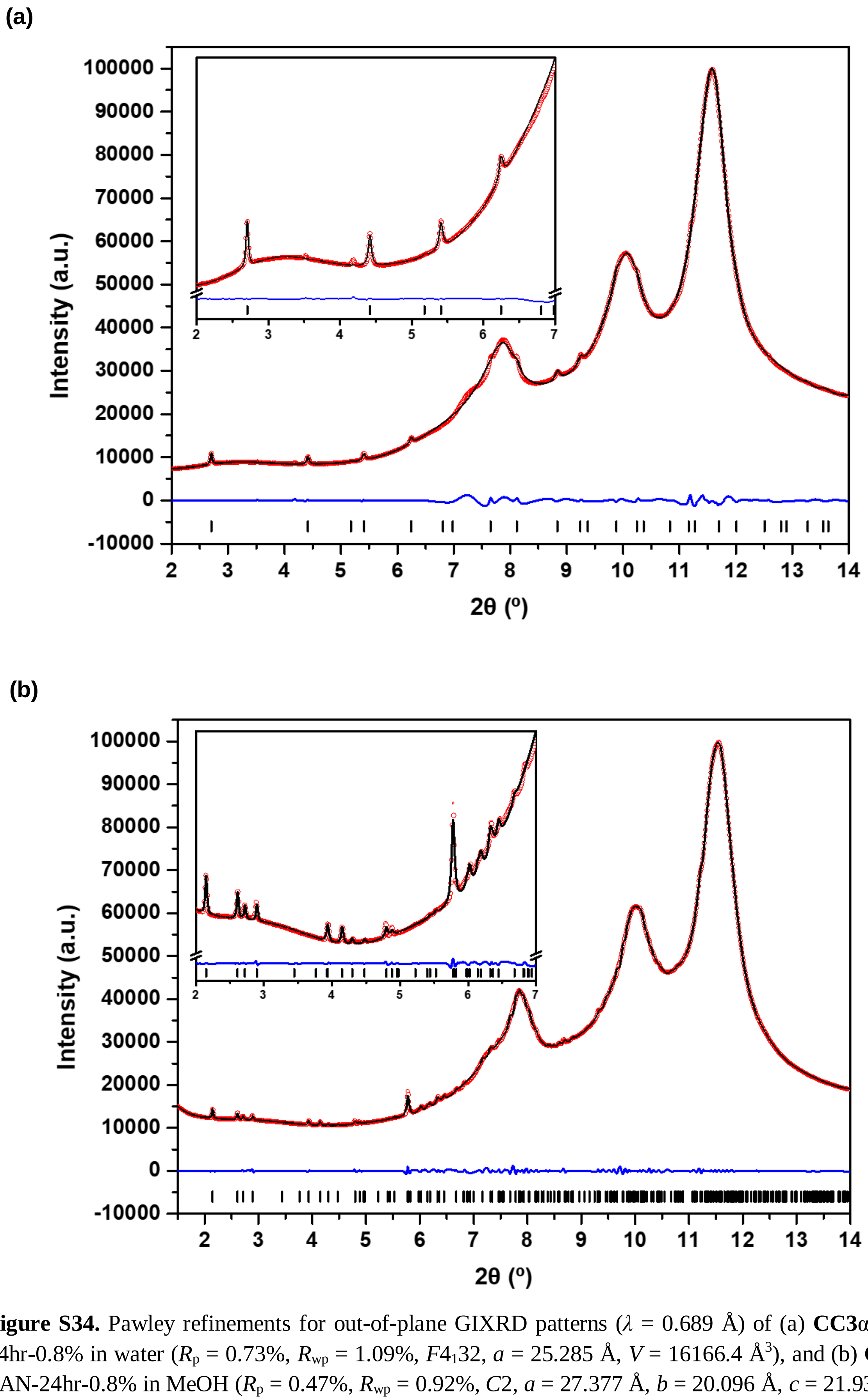

Figure S34. Pawley refinements for out-of-plane GIXRD patterns (λ = 0.689 Å) of (a) **CC3**α-PAN-24hr-0.8% in water (R_p = 0.73%, R_{wp} = 1.09%, $F4_132$, a = 25.285 Å, V = 16166.4 Å^3), and (b) **CC3**γ'-PAN-24hr-0.8% in MeOH (R_p = 0.47%, R_{wp} = 0.92%, $C2$, a = 27.377 Å, b = 20.096 Å, c = 21.936 Å, β = 123.1°, V = 10108.4 Å^3). Red circles: experimental PXRD pattern, black line: fitting pattern, blue curve: difference between experimental and refinement, black bars: reflection positions.

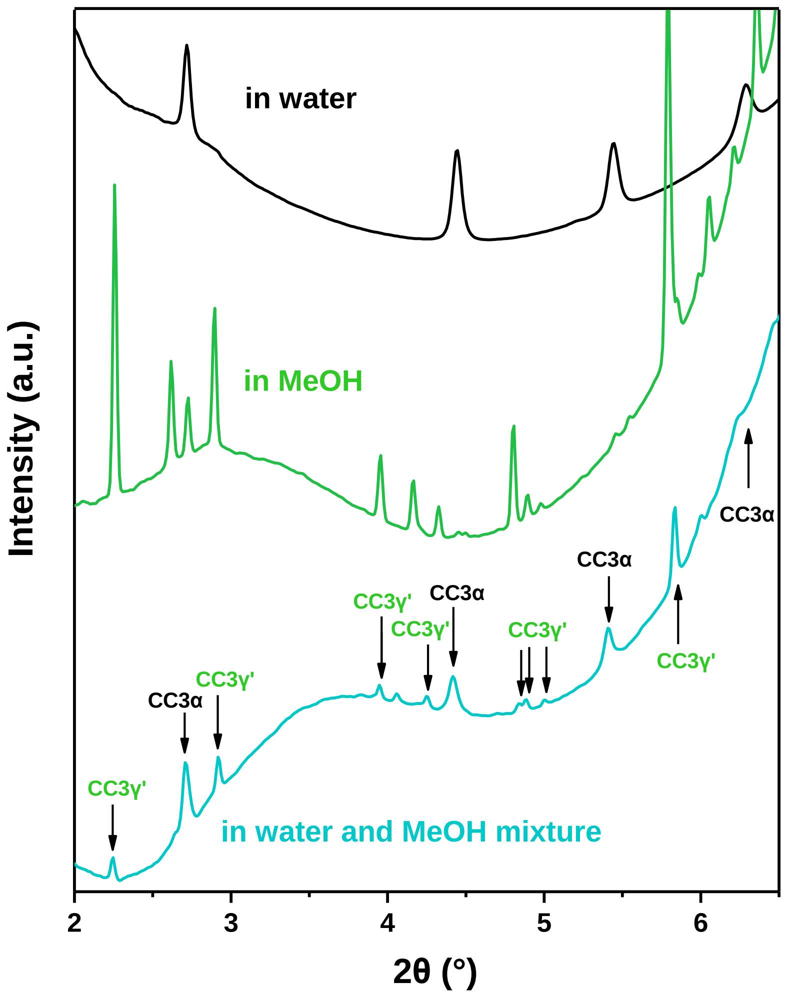

Figure S35. GIXRD characterisation of **CC3**-PAN, **CC3**α-PAN coated in a layer of water (black), **CC3**γ'-PAN coated in a layer of MeOH (green), **CC3**α-PAN that was immersed in water and then also covered in a MeOH solvent layer (cyan) with the diffraction peaks assigned to **CC3**α-PAN and **CC3**γ'-PAN included in the resulting GIXRD pattern.

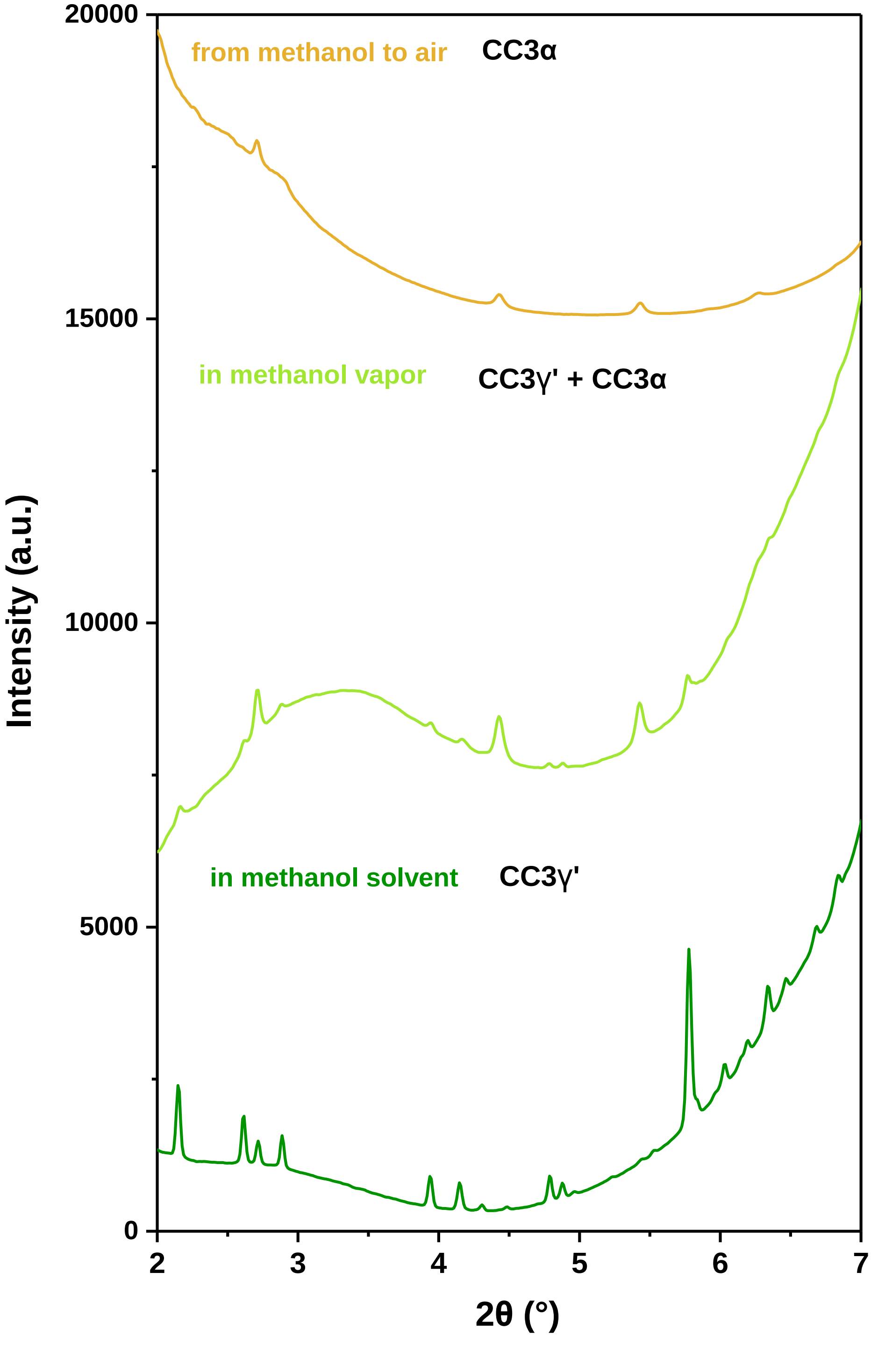

Figure S36. GIXRD characterisation of **CC3** membranes, **CC3**γ'-PAN coated in a layer of MeOH solvent (dark green), after exposing **CC3**α-PAN to MeOH vapour for 30 minutes, after drying **CC3**γ'-PAN in the air for 30 minutes (orange).

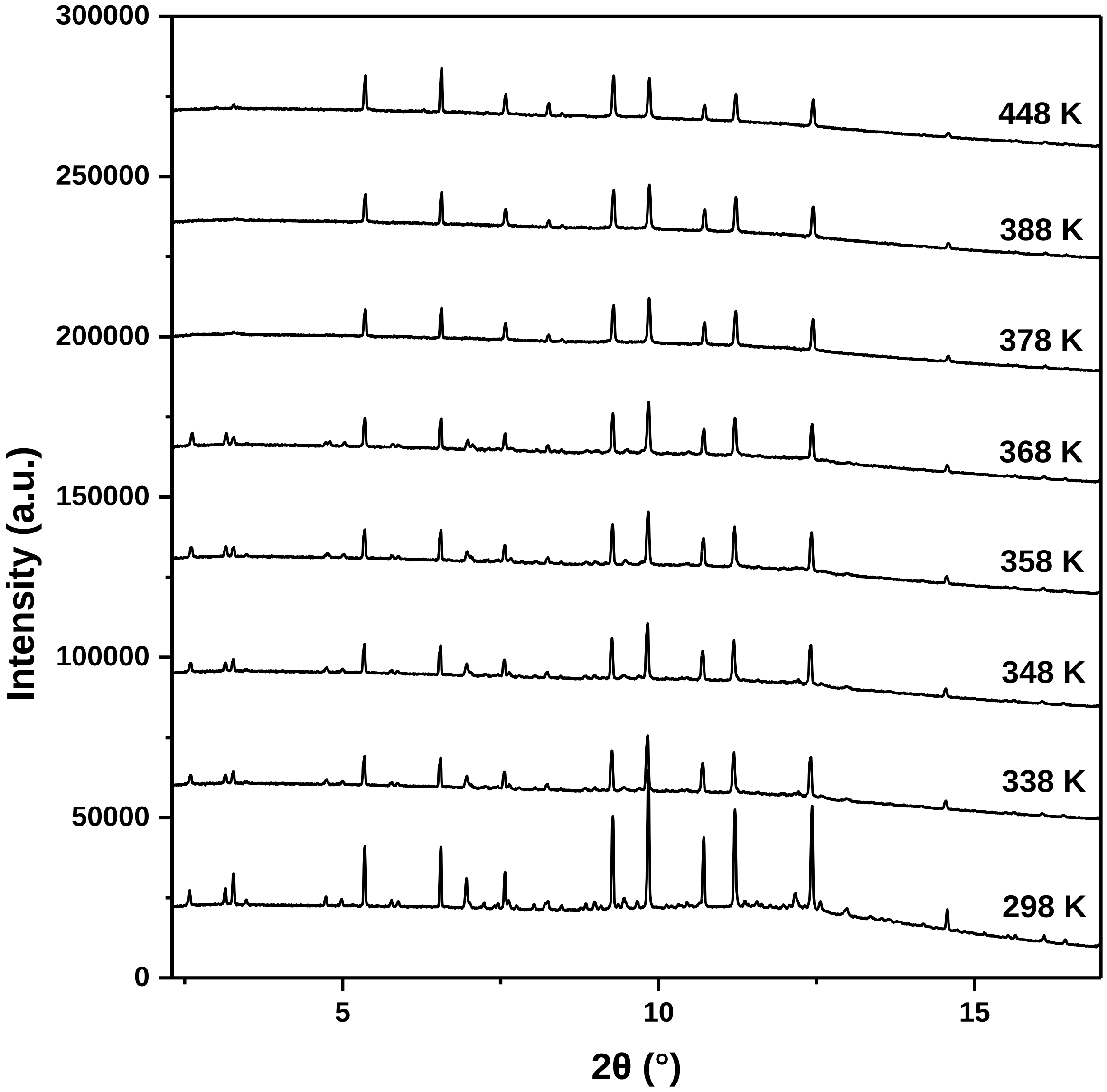

Figure S37. Variable temperature high-resolution PXRD characterisation of bulk powdered sample of the **CC3**γ' suspended in MeOH in a capillary. The **CC3**γ' sample was generated by scraping a **CC3** film off a glass substrate and then suspending the same sample in MeOH.

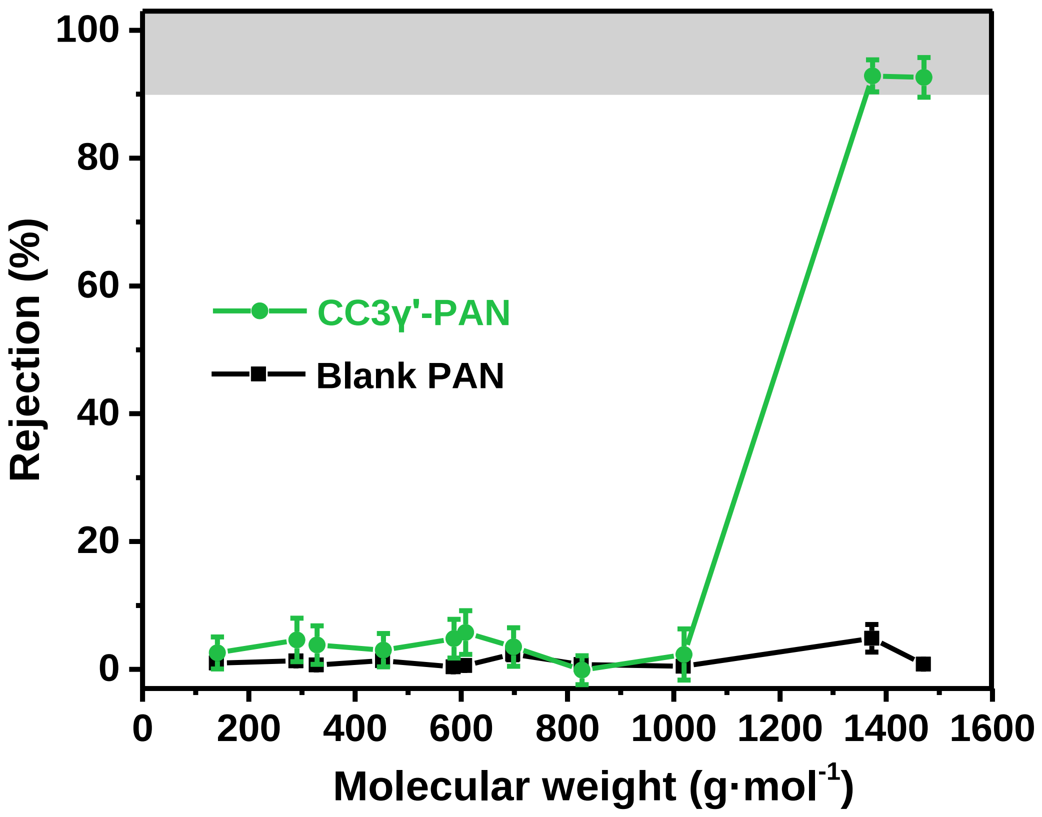

Figure S38. Dye rejection versus molecular weight cut-off curve for **CC3**γ'-PAN and blank PAN membrane in MeOH containing 20 ppm dye solutes. The upstream nitrogen pressure was kept at 10 bar during the measurement and the stirrer agitation speed was 400 rpm. The error bars denote the standard deviations for measurements from at least three independent membranes.

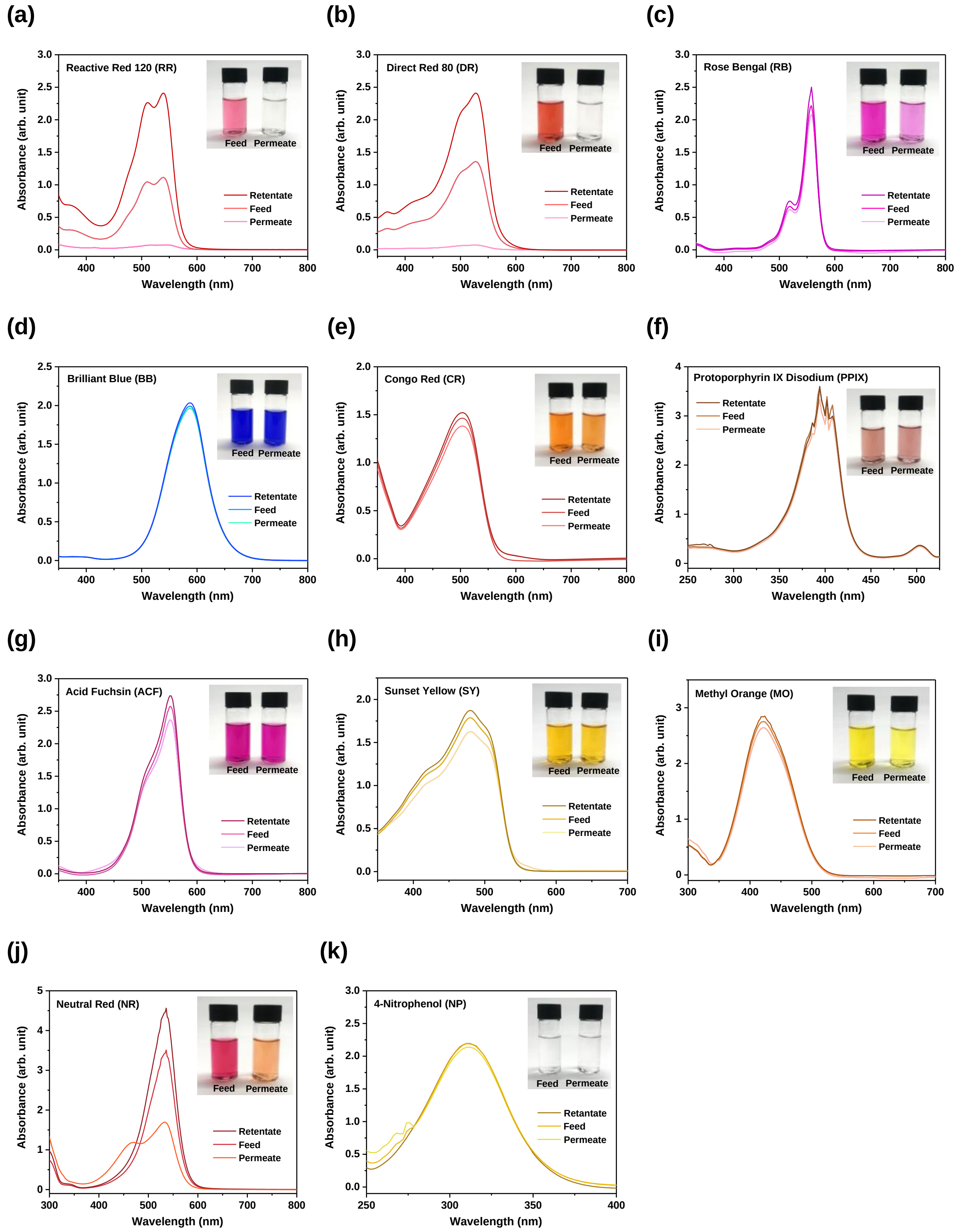

Figure S39. UV absorption spectra of dyes in methanol before and after selectivity tests performed with **CC3**γ'-PAN. Inserts show photographs of the feed and the permeate. Note, NR is a pH indicator, changing from red to yellow between pH 6.8 and 8.0; as the NR permeate was orange in colour the dye concentration was calculated using both yellow and red peaks in the UV absorption spectrum.

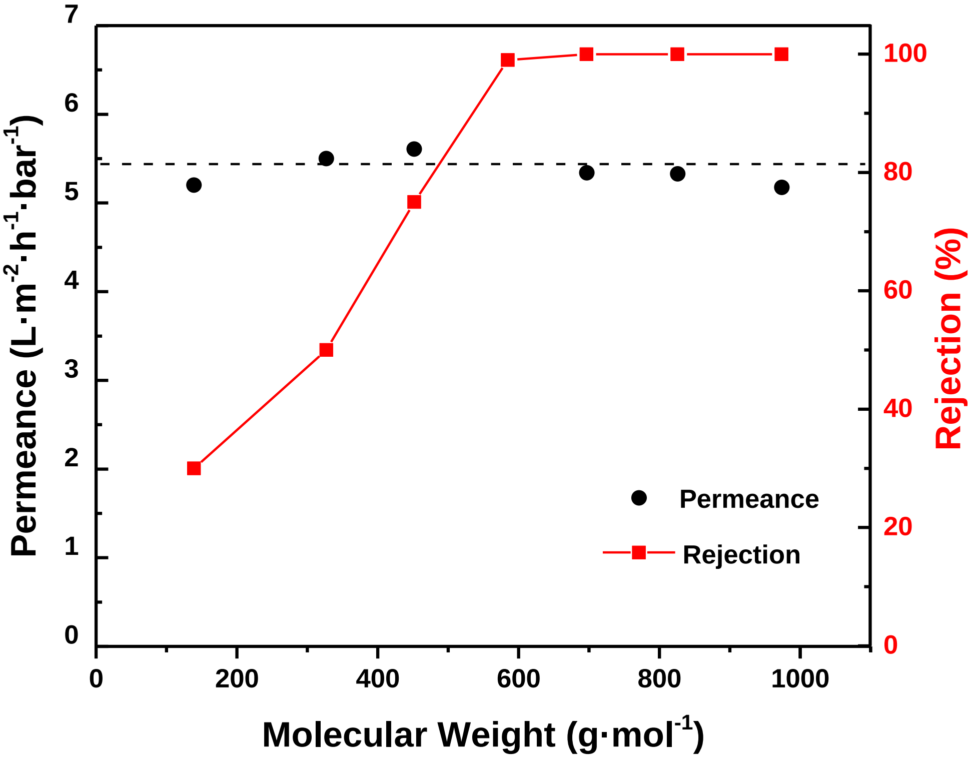

Figure S40. MWCO curve and water permeance for the reference Synder® NDX nanofiltration membrane that exhibits a MWCO cut-off between 500 and 700 g·mol^{-1}.

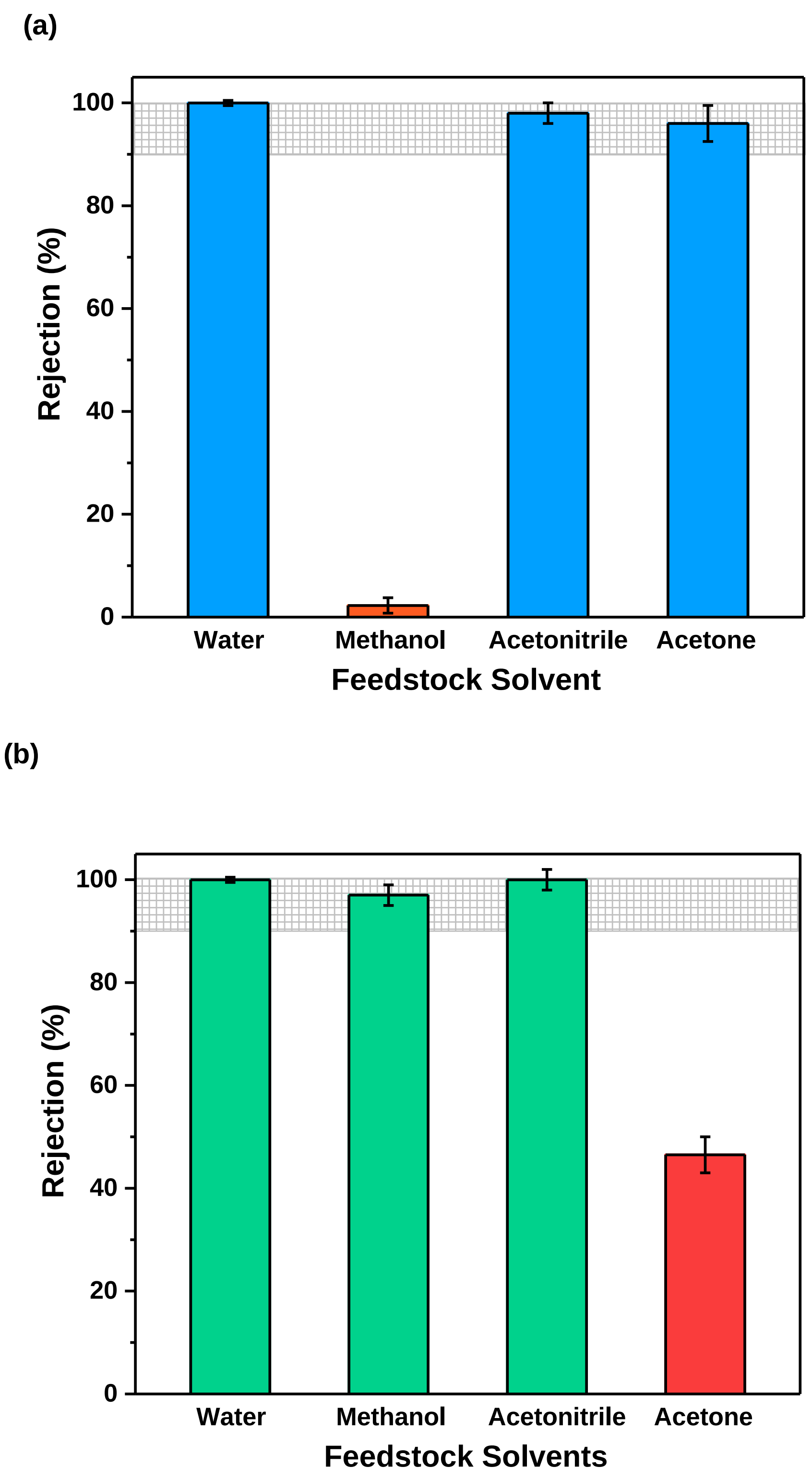

Figure S41. Dye rejection of BB dye in different solvents using, (a) **CC3**α-PAN; (b) the Synder® NDX membrane as the reference. Note, the low dye rejection of BB in acetone using Synder® NDX indicates that this membrane is not resistant to degradation in acetone. All the error bars depict the standard deviations of the data from at least three independent membranes.

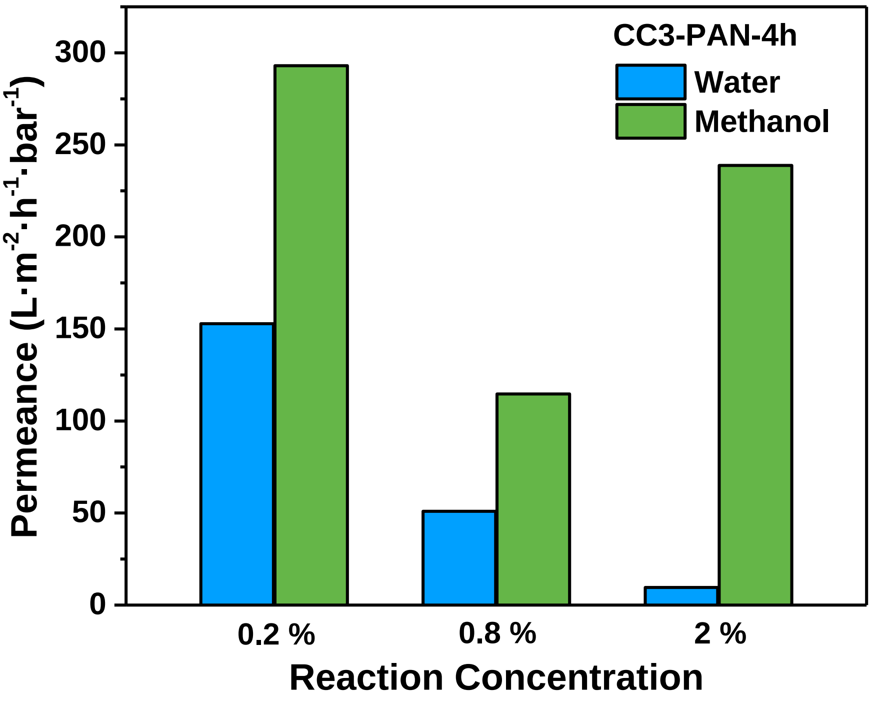

Figure S42. Water and MeOH permeance rates for **CC3**α-PAN-4hr-Y% membranes fabricated using different reagent concentrations: 0.2, 0.8, and 2.0 wt.%. The dead-end cell was kept under a 10 bar nitrogen atmosphere and the stirrer bar agitation speed was 400 rpm. Generic film synthesis conditions, reagent concentration: TFB 0.2, 0.8, or 2.0 wt.% in DCM (30 mL), CHDA 0.2, 0.8, or 2.0 wt.% in water (32 mL); reaction conditions: 4 hours at room temperature; dish diameter: 7.4 cm.

(a)

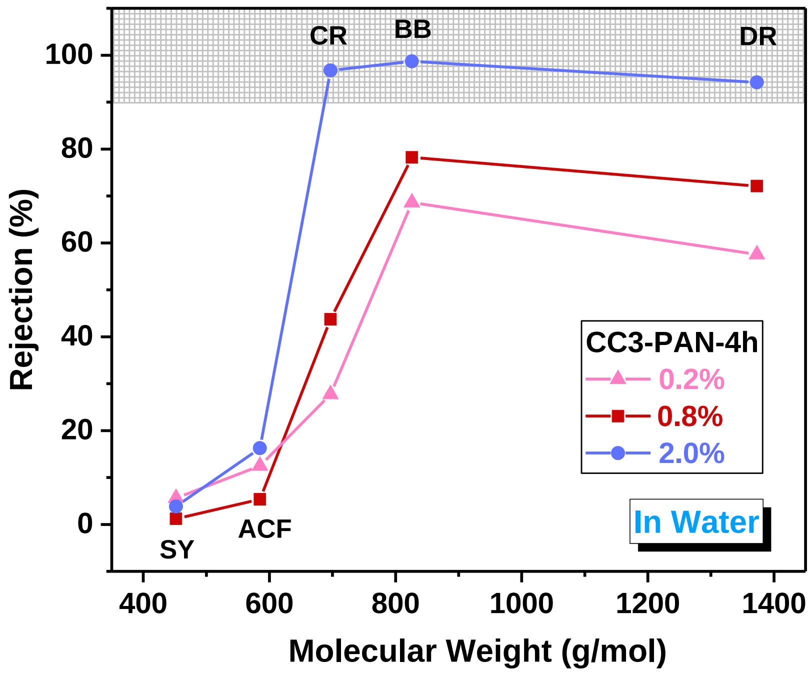

(b)

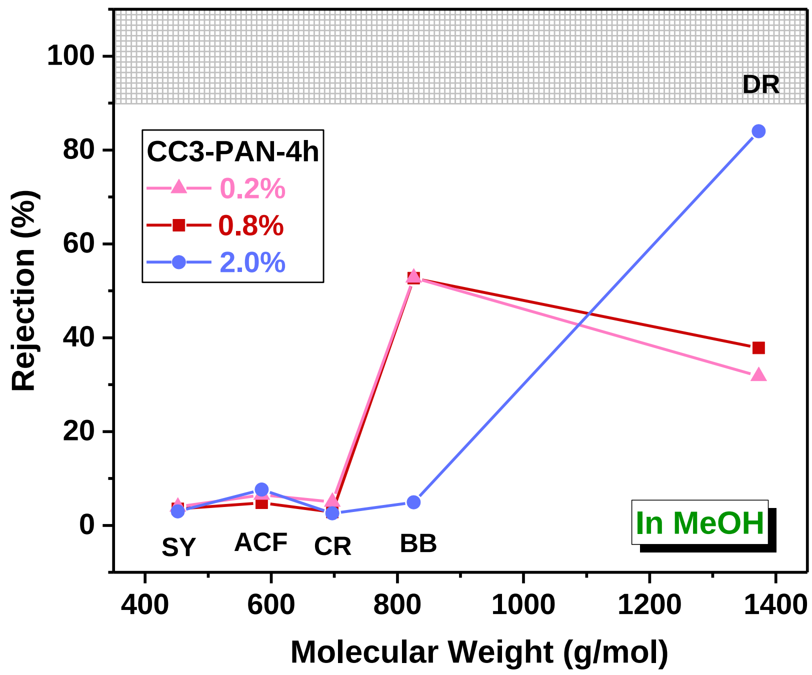

Figure S43. Dye rejection measurements for **CC3**α-PAN-4hr-Y% membranes fabricated using different reagent concentrations: 0.2, 0.8, and 2.0 wt.%. The dead-end cell was kept under a 10 bar nitrogen atmosphere and the stirrer bar agitation speed was 400 rpm. Generic **CC3** film synthesis conditions, reagent concentration: TFB 0.2, 0.8, or 2.0 wt.% in DCM (30 mL), CHDA 0.2, 0.8, or 2.0 wt.% in water (32 mL); reaction conditions: 4 hours at room temperature; dish diameter: 7.4 cm. Dye concentration: 20 ppm.

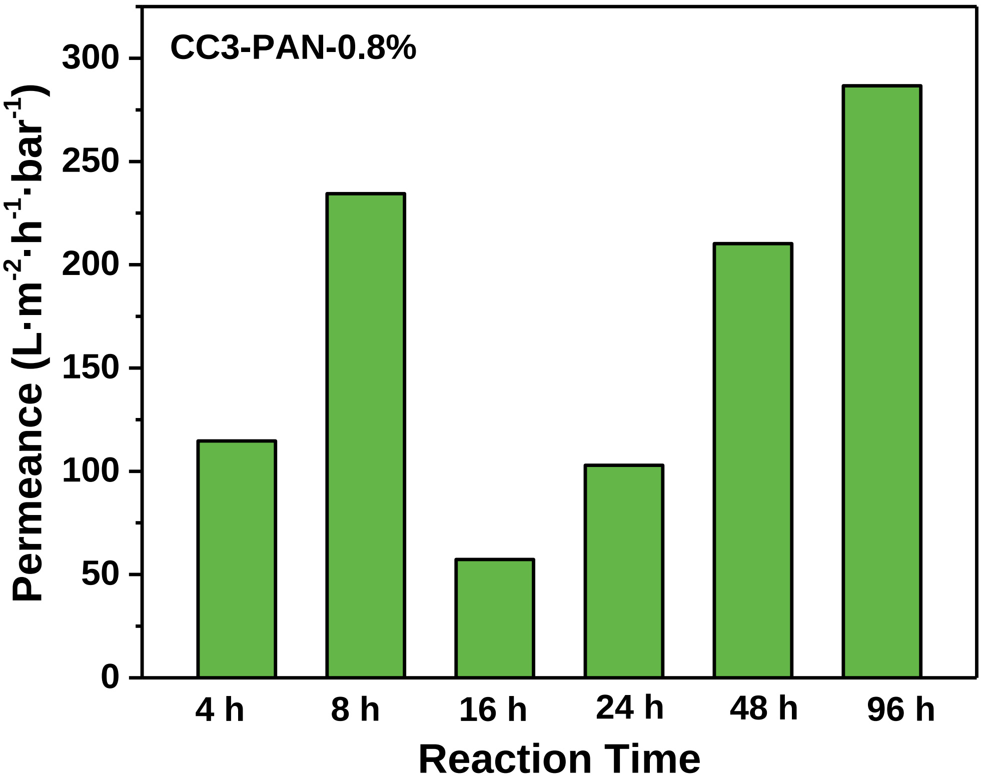

Figure S44. MeOH permeance rates for **CC3**α-PAN-Xhr-0.8% membranes fabricated using reaction times that ranged between 4–96 hours. The dead-end stirred cell was kept under a 10 bar nitrogen atmosphere and the stirrer bar agitation speed was 400 rpm. Generic film synthesis conditions, reagent concentration: TFB 0.8 wt.% in DCM (30 mL), CHDA 0.8 wt.% in water (32 mL); reaction conditions: room temperature; dish diameter: 7.4 cm.

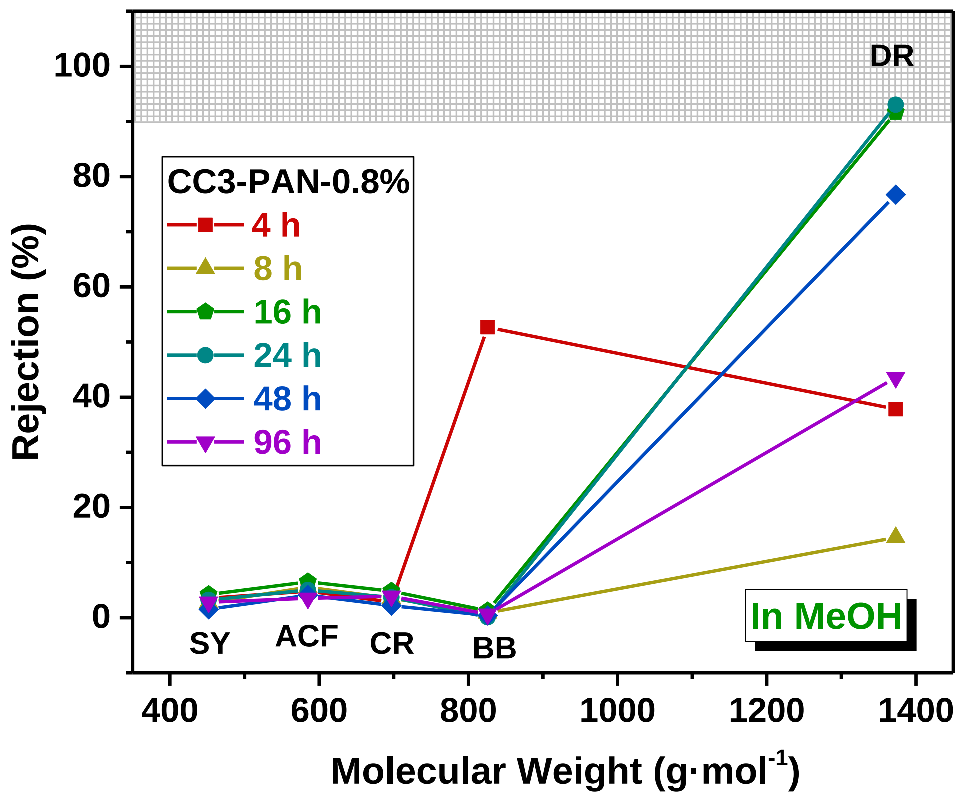

Figure S45. Dye rejection measurements for **CC3**α-PAN-Xhr-0.8% membranes fabricated using reaction times that ranged between 4–96 hours in MeOH. Reaction times of 4 hours were not long enough to form a defect-free **CC3** film and its dye rejection performance was poor. While cracks appeared in the membrane fabricated after 96 hours that contributed to its lower rejection performance. The dead-end cell was kept under a 10 bar nitrogen atmosphere and the stirrer bar agitation speed was 400 rpm. Dye concentration: 20 ppm.

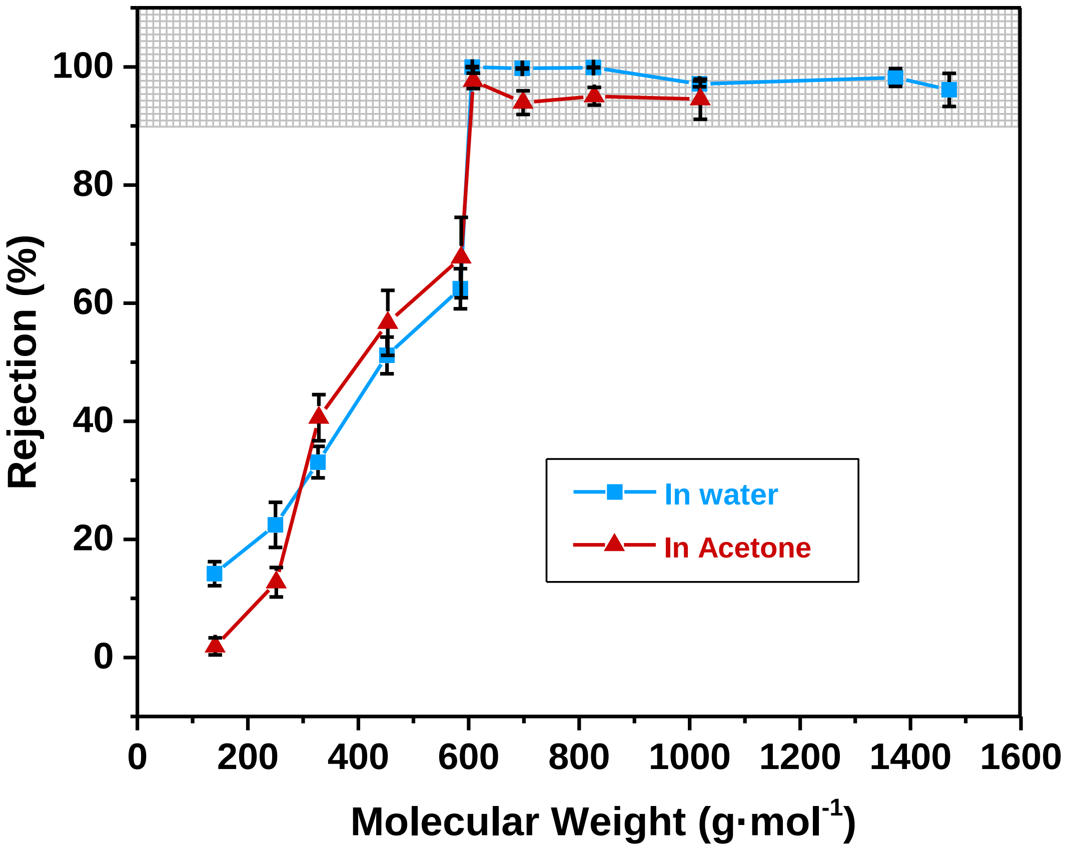

Figure S46. Molecular weight cut-off (MWCO) curve for **CC3**α-PAN determined using organic dyes dissolved in water or acetone. MWCO cut-off curves were obtained plotting dye rejection *versus* the molecular weight of the dyes. The molecular weight corresponding to a rejection of 90% is the MWCO. Dye feedstock concentration: 20 ppm. Note, DR and RR are insoluble in acetone and their dye rejection performance could not be measured. SY and CR have a maximum solubility of 5 ppm in acetone, thus 5 ppm CR in acetone dye solution was used instead of 20 ppm solution. All the error bars depict the standard deviations of the data from at least three independent membranes.

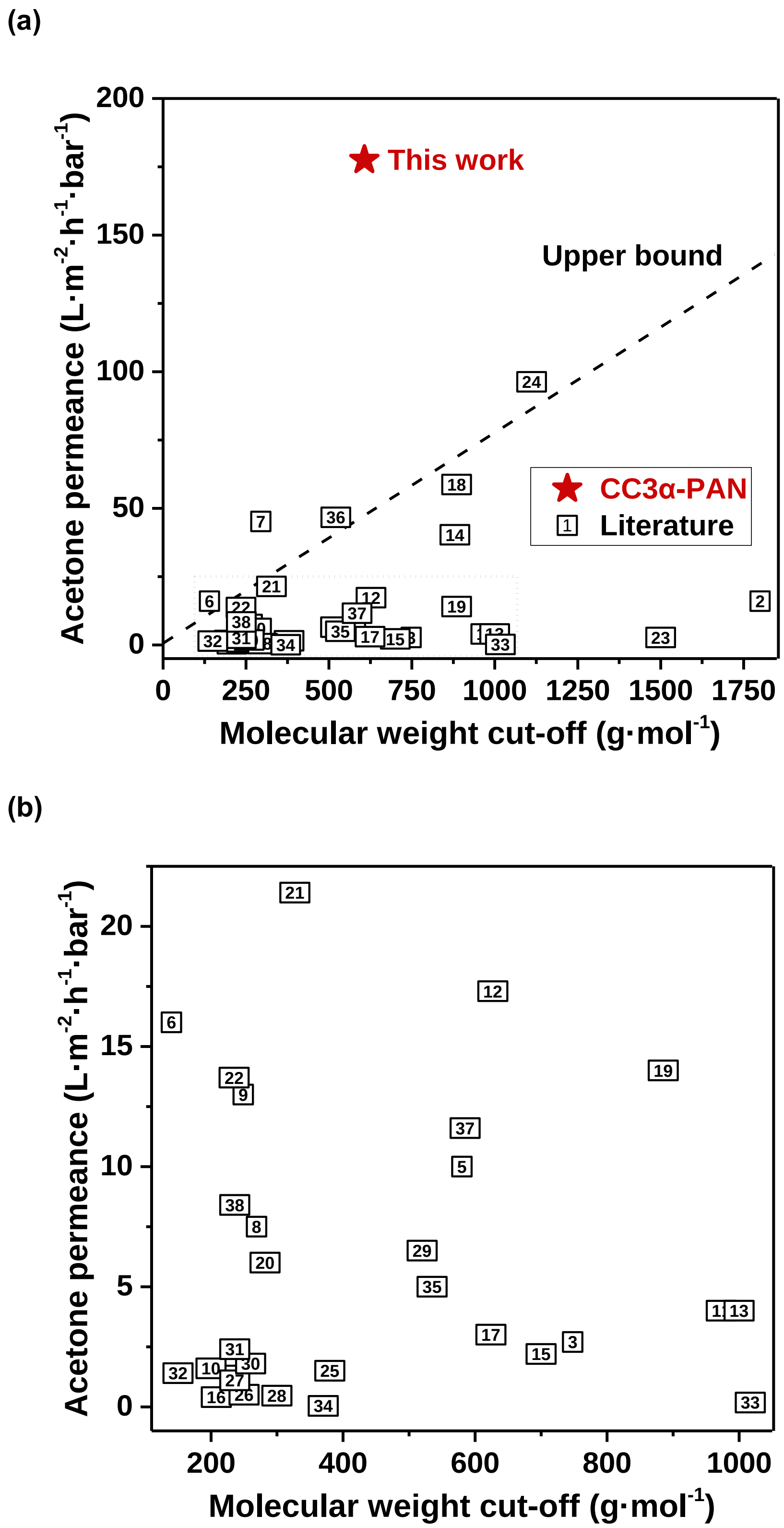

Figure S47. (a) Acetone permeance *versus* MWCO values for nanofiltration membranes reported in the literature in comparison to **CC3α**-PAN. The upper bound has been added to highlight the trade-off between permeance and rejection, where rejection performance negatively correlates with molecular weight cut-off. The area with dash square is shown in higher resolution in (b). Further information about the membranes shown in the plot is included in **Table S9**.

Table S9. Detailed information about the acetone permeance and MWCO values in acetone for the membranes reported in the literature or are commercially available. The permeance values with different units have been transferred to ($L \cdot m^{-2} \cdot h^{-1} \cdot bar^{-1}$) for comparison. All the membranes cited here were tested in acetone. POC, porous organic cage; MOF, metal-organic framework; MC, macrocycle; NPs, nanoparticles; GO, graphene oxide; PTMSP, poly[1-(trimethylsilyl)-1-propyne]; PA, polyamide; PEI, polyethyleneimine; PANI, polyaniline; PI, polyimide; PE, polyethylene; PEEK, Poly(ether ether ketone); PEO, poly(ethylene oxide); PIM, polymers of intrinsic microporosity; PIP, piperazine; PAR, polyarylate.

No.	MWCO ($g \cdot mol^{-1}$)	P ($L \cdot m^{-2} \cdot h^{-1} \cdot bar^{-1}$)	Solvent	Solute	Process configuration	Membrane type	Membrane Material	Year	Reference
This work	607	177.5	Acetone	Protoporphyrin IX disodium	Dead-end cell	POC	cages on polyacrylonitrile ultrafiltration membrane	2020	-
2	1800	16.0	Acetone	Polystyrene	Cross-flow	MOF	HKUST-1 / P84	2014	[16]
3	748	2.7	Acetone	Clarithromycin	Cross-flow	MC	β-cyclodextrin film composite (TFC)	2019	[17]
4	236	1.3	Acetone	Methylstyrene dimer	Cross-flow	NPs	P[N-isopropylacrylamide- 2-(hydroxy) ethyl methacrylate] nanoparticles /crosslinked P84	2013	[18]
5	580	10.0	Acetone	Styrene oligomers	Cross-flow	NPs	Grignard grafted TiO_2/ alumina	2014	[19]
6	140	16.0	Acetone	Oligostyrene	Cross-flow	GO	Covalently cross-linked polybenzimidazole/graphene oxide membranes	2018	[20]
7	295	45.2	Acetone	Oligostyrene	Cross-flow	GO	Non-crosslinked polybenzimidazole/graphene oxide membranes	2018	[20]

Table S9 cont.

8	269	7.5	Acetone	Methyl red	Dead-end cell	GO	Graphene oxide hollow fibre membranes with a porous poly(methyl methacrylate) sacrificial layer	2015	[21]
9	249	13.0	Acetone	Chrysoi dine G	Dead-end cell	GO	Highly laminated graphene oxide membranes	2017	[22]
10	200	1.6	Acetone	Polyeth ylene glycol	Dead-end cell	GO	Polyethyleneimin e-graphene oxide layer on dopamine coadedpolyacrylo nitrile ultrafiltration membrane	2015	[23]
11	973	4.0	Acetone	Rose bengal	Dead-end cell	GO	Graphene oxide-layered hollow fibers	2020	[24]
12	627	17.3	Acetone	Remazo l brilliant blue R	Cross-flow	PTM SP	Poly[1-(trimethylsilyl)-1-propyne] (PTMSP) / poly(acrylonitrile) (PAN)	2009	[25]
13	1000	4.0	Acetone	Soybean oil	Dead-end cell	Com merc ial	SolSep-NF030306	2011	[26]
14	880	40.3	Acetone	Erythros ine B	Cross-flow	Com merc ial	SolSep-169	2006	[27]
15	700	2.2	Acetone	Bromot hymol blue	Cross-flow	Com merc ial	MPF-50	2006	[27]
16	208	0.4	Acetone	Eusolex	Cross-flow	Com merc ial	HITK-T1	2006	[27]
17	624	3.0	Acetone	Bromot hymol blue	Cross-flow	Com merc ial	FSTi-128	2006	[27]
18	885	58.7	Acetone	Triglyce rides	Dead-end cell	Com merc ial	SolSep-NF010206	2018	[28]
19	885	14.0	Acetone	Triglyce rides	Dead-end cell and cross-flow	PA	[Poly(amide-b-ether) copolymer] top layer membrane	1999	[29]
20	282	6.0	Acetone	Oleic acid	Cross-flow	PA	Polyamide (PA)/PAN	2002	[30]

Table S9 cont.

21	327	21.4	Acetone	Methyl orange	Dead-end cell	PA	m-Xylylenediamine (m-XDA) - trimesoyl chloride (TMC) membranes	2020	[31]
22	235	13.7	Acetone	Styrene dimer	Cross-flow	PA	Plant-based monomer TFC membranes	2021	[32]
23	1500	2.7	Acetone	Polyethylene glycol	Dead-end cell	PEI	Polyethyleneimine	2015	[23]
24	1111	96.3	Acetone	Acid yellow 79	Dead-end cell	PEI	Polydopamine-polyethyleneimine on polyacrylonitrile ultrafiltration membrane	2018	[33]
25	380	1.5	Acetone	Oligostyrene	Hollow fibre testing module	PANI	Polyaniline hollow fibres	2008	[34]
26	250	0.5	Acetone	Oligostyrene	Spiral-wound module	PANI	Polyaniline spiral-wound module	2010	[35]
27	236	1.1	Acetone	Oligostyrene	Dead-end cell and cross-flow	PANI	Cross-linked Polyaniline (PANI)	2009	[36]
28	300	0.46	Acetone	Poly(propylene) glycols	Dead-end cell and cross-flow	PANI	PANI-poly(2-acrylamido-2-methyl-1-propanesulfonic acid) (PAMPSA) membranes	2019	[37]
29	520	6.5	Acetone	Oligostyrene	Cross-flow	PI	P84 (PI)	2014	[38]
30	260	1.8	Acetone	Oligostyrene	Cross-flow	PI	Crosslinked polyamide–imide (Torlon®)	2013	[39]
31	236	2.4	Acetone	Methylstyrene dimer	Dead-end cell	PI	Polyamide (PA)/crosslinked P84 PI	2012	[40]
32	150	1.4	Acetone	Triethylene glycol	Dead-end cell	PE	Teflon AF2400/polyethylene (PE) membranes	2020	[41]

Table S9 cont.

33	1017	0.18	Acetone	Rose bengal	Dead-end cell	PEEK	Poly(ether ether ketone) (PEEK) membranes crosslinked with diamines	2013	[42]
34	370	0.04	Acetone	Ethylene glycol oligomers	Dead-end cell	PEO	[polystyrene-block-poly(ethylene oxide)/ homopolymer] /alumina	2010	[43]
35	535	5.0	Acetone	Hexaphenylbenzene	Dead-end cell	PIM	[polymers of intrinsic microporosity (PIM-1)/ polyethyleneimine(PEI)]/PAN	2012	[44]
36	521	46.7	Acetone	Mometasone furoate	Molecular simulation	PIM	Functional PIM-1 membranes	2019	[45]
37	585	11.6	Acetone	Acid fuchsin	Cross-flow	PIP	Polyamide-based polyamide/piperazine (PEI/PIP) polyimide hollow fibre membranes	2020	[46]
38	236	8.4	Acetone	Methyl styrene dimer	Dead-end cell and cross-flow	PAR	Polyarylate (PAR) /PI nanofilm	2016	[47]

3.0 Additional Discussion

3.1 Pre-treatment Membrane Performance of CC3-PAN

Membrane Pre-treatment. The as-prepared **CC3**-PAN membranes were soaked in water (without drying) at room temperature for 1 day, 3 days, and 7 days, to generate membranes referred to hereafter as **CC3**-PAN, **CC3**-PAN-3, **CC3**-PAN-7, respectively, while the as-prepared membrane is abbreviated as **CC3**-PAN-0.

Membrane Performance. The native water permeance of **CC3**α-PAN-0 was 6.1 $L{\cdot}m^{-2}{\cdot}h^{-1}{\cdot}bar^{-1}$, but it improved to 43.0 $L{\cdot}m^{-2}{\cdot}h^{-1}{\cdot}bar^{-1}$ for **CC3**α-PAN, 60.2 $L{\cdot}m^{-2}{\cdot}h^{-1}{\cdot}bar^{-1}$ for **CC3**α-PAN-3, and 145.7 $L{\cdot}m^{-2}{\cdot}h^{-1}{\cdot}bar^{-1}$ for **CC3**α-PAN-7, as shown in **Figure S48**. Increased MeOH permeances were observed in methanol after soaking the membrane for longer durations in water beforehand and were determined to be 15.1 $L{\cdot}m^{-2}{\cdot}h^{-1}{\cdot}bar^{-1}$ for **CC3**γ'-PAN-0, 85.1 $L{\cdot}m^{-2}{\cdot}h^{-1}{\cdot}bar^{-1}$ for **CC3**γ'-PAN, 159.0 $L{\cdot}m^{-2}{\cdot}h^{-1}{\cdot}bar^{-1}$ for **CC3**γ'-PAN-3, and 324.8 $L{\cdot}m^{-2}{\cdot}h^{-1}{\cdot}bar^{-1}$ for **CC3**γ'-PAN-7. Despite the significant improvements in solvent permanence after pre-treatment in water, the dye rejection performance **CC3**α-PAN-0, **CC3**α-PAN, **CC3**α-PAN-3, **CC3**α-PAN-7 were comparable. As shown in **Figure S49**, these treated membranes were found to have comparable MWCO of ~ 600 $g{\cdot}mol^{-1}$ under the same measurement conditions.

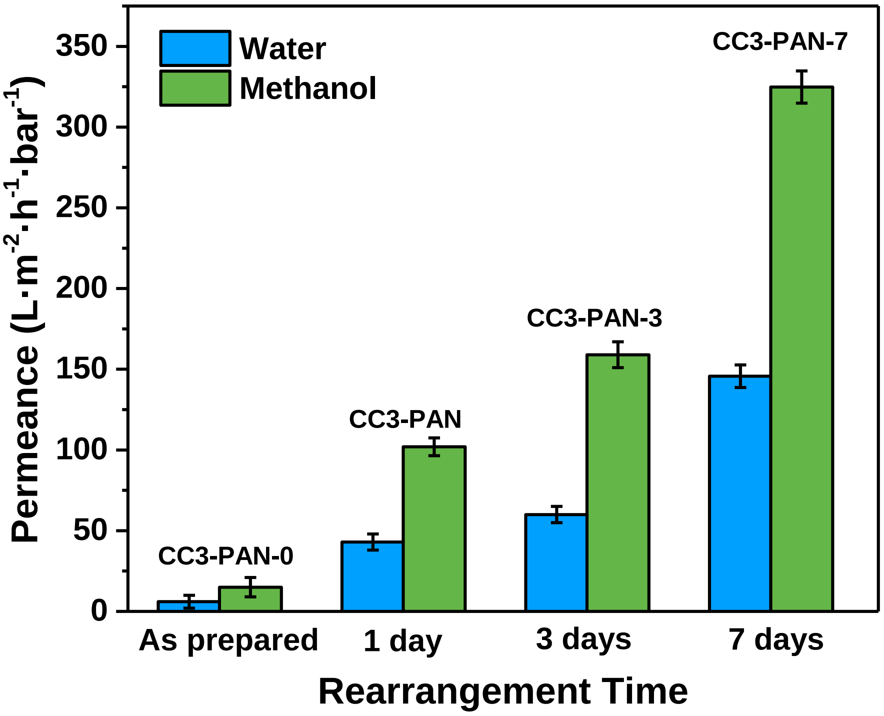

Figure S48. Water and MeOH permeance of the as-prepared membrane (**CC3**-PAN-0) and the water treated **CC3**-PAN, **CC3**-PAN-3, and **CC3**-PAN-7 membranes. All the error bars depict the standard deviations of the data from at least three independent membranes.

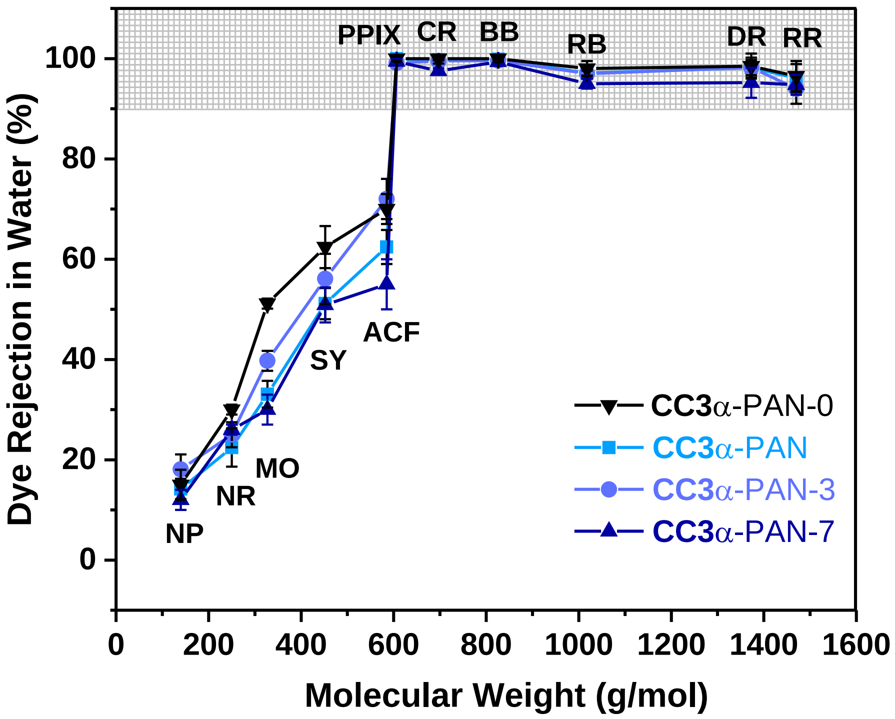

Figure S49. MWCO curves for dyes for as-synthesised **CC3**α-PAN-0, **CC3**α-PAN, **CC3**α-PAN-3, **CC3**α-PAN-7 in water. All the error bars depict the standard deviations of the data from at least three independent membranes.

Figures Showing Experimental and Characterisation Setup

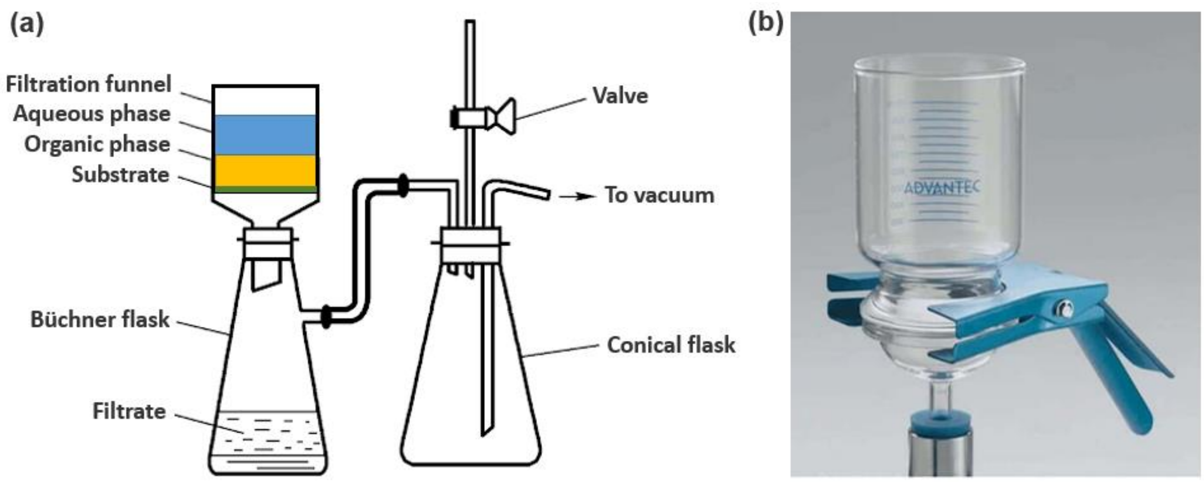

Figure S50. Reactions set-up for the fabrication of **CC3** films. (a) Schematic diagram of filtration funnel system connected to vacuum; (b) Photograph of the AdvanTec® glass filtration funnel with a diameter of 7.4 cm.

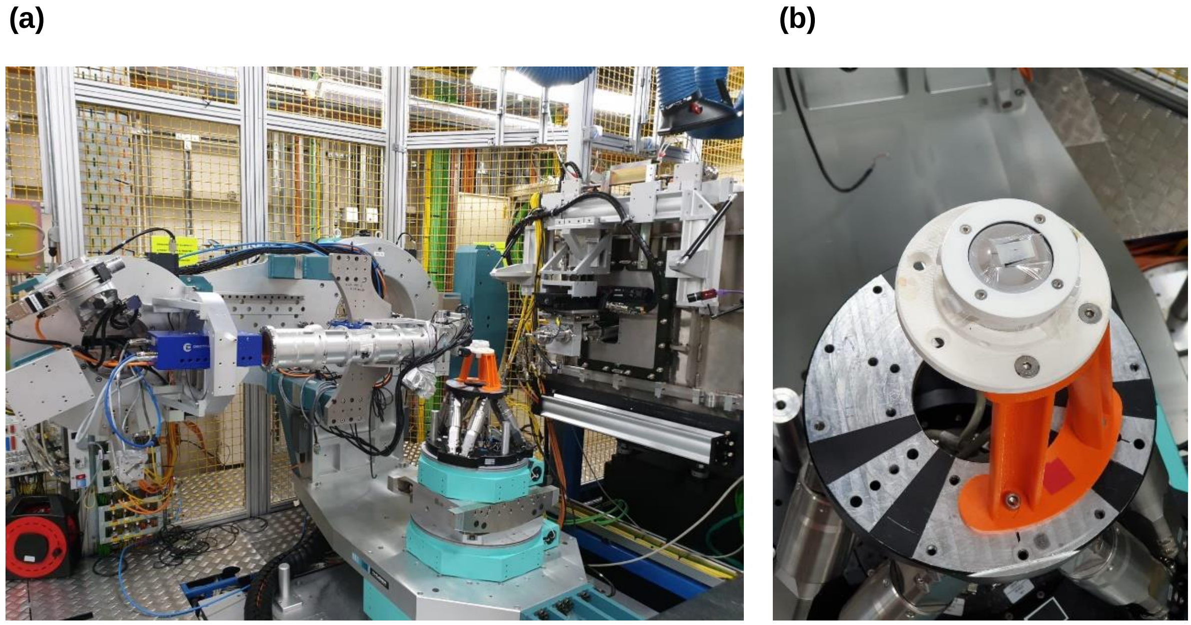

Figure S51. Image showing (a) experimental set-up at beamline I07; (b) a membrane on a glass substrate mounted on the hexapod sample holder and covered with a Mylar film.

4.0 References

1. Tozawa, T. *et al.* Porous organic cages. *Nat. Mater.* **8**, 973–978 (2009).
2. Jiang, S. *et al.* Molecular dynamics simulations of gas selectivity in amorphous porous molecular solids. *J. Am. Chem. Soc.* **135**, 17818–17830 (2013).
3. Thompson, S. P. *et al.* Fast X-ray powder diffraction on I11 at Diamond. *J. Synchrotron Radiat.* **18**, 637–648 (2011).
4. Parker, J. E., Potter, J., Thompson, S. P., Lennie, A. R. Tang, C. C. In situ gas supply system on the powder diffraction beamline I11 at Diamond Light Source. *Mater. Sci. Forum* (2012).
5. TOPAS Academic version 4.1. *Coelho Software, Brisbane, Australia* (2007).
6. R Core Team. R: A language and environment for statistical computing. (2020).
7. David R. Lide. CRC handbook of chemistry and physics: A ready-reference of chemical and physical data, 85th edition. *J. Am. Chem. Soc.* **127**, 4542–4542 (2005).
8. C. M. Hansen. Hansen solubility parameters: A user's handbook, Second edition. *CRC Press, Boca Raton, FL* (2007).
9. J. A. Riddick, W. B. B. Organic solvents, Third edition. *J. Pharm. Sci.* **60**, 1112 (1971).
10. B. E. Poling, J. M. Prausnitz, J. P. O. Properties of gases and liquids, Fifth edition. *McGraw-Hill Education, London* (2001).
11. Van Der Bruggen, B., Schaep, J., Wilms, D. & Vandecasteele, C. Influence of molecular size, polarity and charge on the retention of organic molecules by nanofiltration. *J. Memb. Sci.* **156**, 29–41 (1999).
12. Korson, L., Drost-Hansen, W. & Millero, F. J. Viscosity of water at various temperatures. *J. Phys. Chem.* **73**, 34–39 (1969).
13. Buekenhoudt, A. *et al.* Unravelling the solvent flux behaviour of ceramic nanofiltration and ultrafiltration membranes. *J. Memb. Sci.* **439**, 36–47 (2013).
14. Aminabhavi, T. M., Patil, V. B., Aralaguppi, M. L. & Phayde, H. T. S. Density, viscosity, and refractive index of the binary mixtures of cyclohexane with hexane, heptane, octane, nonane, and decane at (298.15, 303.15, and 308.15) K. *J. Chem. Eng. Data* **41**, 521–525 (1996).
15. Hasell, T., Chong, S. Y., Jelfs, K. E., Adams, D. J. & Cooper, A. I. Porous organic cage nanocrystals by solution mixing. *J. Am. Chem. Soc.* **134**, 588–598 (2012).
16. Campbell, J., Székely, G., Davies, R. P., Braddock, D. C. & Livingston, A. G. Fabrication of hybrid polymer/metal organic framework membranes: Mixed matrix membranes versus in situ growth. *J. Mater. Chem. A* **2**, 9260–9271 (2014).
17. Xu, S. J., Shen, Q., Xu, Z. L. & Dong, Z. Q. Novel designed TFC membrane based on host-guest interaction for organic solvent nanofiltration (OSN). *J. Memb. Sci.* **588**, 117227 (2019).
18. Siddique, H. *et al.* Membranes for organic solvent nanofiltration based on preassembled nanoparticles. *Ind. Eng. Chem. Res.* **52**, 1109–1121 (2013).
19. Rezaei Hosseinabadi, S. *et al.* Organic solvent nanofiltration with Grignard functionalised

ceramic nanofiltration membranes. *J. Memb. Sci.* **454**, 496–504 (2014).

20. Fei, F., Cseri, L., Szekely, G. & Blanford, C. F. Robust covalently cross-linked polybenzimidazole/graphene oxide membranes for high-flux organic solvent nanofiltration. *ACS Appl. Mater. Interfaces* **10**, 16140–16147 (2018).
21. Chong, J. Y., Aba, N. F. D., Wang, B., Mattevi, C. & Li, K. UV-enhanced sacrificial layer stabilised graphene oxide hollow fibre membranes for nanofiltration. *Sci. Rep.* **5**, 1–11 (2015).
22. Yang, Q. *et al.* Ultrathin graphene-based membrane with precise molecular sieving and ultrafast solvent permeation. *Nat. Mater.* **16**, 1198–1202 (2017).
23. Ding, R. *et al.* Graphene oxide-embedded nanocomposite membrane for solvent resistant nanofiltration with enhanced rejection ability. *Chem. Eng. Sci.* **138**, 227–238 (2015).
24. Mahalingam, D. K. *et al.* Spray-coated graphene oxide hollow fibers for nanofiltration. *J. Memb. Sci.* **606**, 118006 (2020).
25. Volkov, A. V. *et al.* High permeable PTMSP/PAN composite membranes for solvent nanofiltration. *J. Memb. Sci.* **333**, 88–93 (2009).
26. Darvishmanesh, S., Robberecht, T., Luis, P., Degrè, J. & Van Der Bruggen, B. Performance of nanofiltration membranes for solvent purification in the oil industry. *J. Am. Oil Chem. Soc.* **88**, 1255–1261 (2011).
27. Geens, J., Boussu, K., Vandecasteele, C. & Van der Bruggen, B. Modelling of solute transport in non-aqueous nanofiltration. *J. Memb. Sci.* **281**, 139–148 (2006).
28. Nur Fitrah Awang Ismail, D. & Faisal Ghazali, N. Separation of fatty acids from palm oil using organic solvent nanofiltration. *Malaysian J. Anal. Sci.* **22**, 561–569 (2018).
29. Zwijnenberg, H. J., Krosse, A. M., Ebert, K., Peinemann, K. V. & Cuperus, F. P. Acetone-stable nanofiltration membranes in deacidifying vegetable oil. *JAOCS, J. Am. Oil Chem. Soc.* **76**, 83–87 (1999).
30. Kim, I.-C., Jegal, J. & Lee, K.-H. Effect of aqueous and organic solutions on the performance of polyamide thin-film-composite nanofiltration membranes. *J. Polym. Sci. Part B Polym. Phys.* **40**, 2151–2163 (2002).
31. Li, Y., Li, S., Zhu, J., Volodine, A. & Van der Bruggen, B. Controllable synthesis of a chemically stable molecular sieving nanofilm for highly efficient organic solvent nanofiltration. *Chem. Sci.* **11**, 4263–4271 (2020).
32. Park, S.-H. *et al.* Hydrophobic thin film composite nanofiltration membranes derived solely from sustainable sources. *Green Chem.* (2021) doi:10.1039/d0gc03226c.
33. Wu, X. *et al.* Adsorption-assisted interfacial polymerisation toward ultrathin active layers for ultrafast organic permeation. *ACS Appl. Mater. Interfaces* **10**, 10445–10453 (2018).
34. Loh, X. X. *et al.* Polyaniline hollow fibres for organic solvent nanofiltration. *Chem. Commun.* 6324–6326 (2008).
35. Sairam, M. *et al.* Spiral-wound polyaniline membrane modules for organic solvent nanofiltration

(OSN). *J. Memb. Sci.* **349**, 123–129 (2010).

36. Loh, X. X. *et al.* Crosslinked integrally skinned asymmetric polyaniline membranes for use in organic solvents. *J. Memb. Sci.* **326**, 635–642 (2009).
37. Sarihan, A. *et al.* Exploiting the electrical conductivity of poly-acid doped polyaniline membranes with enhanced durability for organic solvent nanofiltration. *J. Memb. Sci.* **579**, 11–21 (2019).
38. Siddique, H., Bhole, Y., Peeva, L. G. & Livingston, A. G. Pore preserving crosslinkers for polyimide OSN membranes. *J. Memb. Sci.* **465**, 138–150 (2014).
39. Dutczak, S. M., Cuperus, F. P., Wessling, M. & Stamatialis, D. F. New crosslinking method of polyamide-imide membranes for potential application in harsh polar aprotic solvents. *Sep. Purif. Technol.* **102**, 142–146 (2013).
40. Jimenez Solomon, M. F., Bhole, Y. & Livingston, A. G. High flux membranes for organic solvent nanofiltration (OSN)-Interfacial polymerisation with solvent activation. *J. Memb. Sci.* **423–424**, 371–382 (2012).
41. Shi, G. M. & Chung, T. S. Teflon AF2400/polyethylene membranes for organic solvent nanofiltration (OSN). *J. Memb. Sci.* **602**, 117972 (2020).
42. Hendrix, K., Van Eynde, M., Koeckelberghs, G. & Vankelecom, I. F. J. Crosslinking of modified poly(ether ether ketone) membranes for use in solvent resistant nanofiltration. *J. Memb. Sci.* **447**, 212–221 (2013).
43. Li, X. *et al.* Ordered nanoporous membranes based on diblock copolymers with high chemical stability and tunable separation properties. *J. Mater. Chem.* **20**, 4333–4339 (2010).
44. Fritsch, D., Merten, P., Heinrich, K., Lazar, M. & Priske, M. High performance organic solvent nanofiltration membranes: Development and thorough testing of thin film composite membranes made of polymers of intrinsic microporosity (PIMs). *J. Memb. Sci.* **401–402**, 222–231 (2012).
45. Xu, Q. & Jiang, J. Effects of functionalisation on the nanofiltration performance of PIM-1: Molecular simulation investigation. *J. Memb. Sci.* **591**, 117357 (2019).
46. Goh, K. S. *et al.* Thin-film composite hollow fibre membrane for low pressure organic solvent nanofiltration. *J. Memb. Sci.* **597**, 117760 (2020).
47. Jimenez-Solomon, M. F., Song, Q., Jelfs, K. E., Munoz-Ibanez, M. & Livingston, A. G. Polymer nanofilms with enhanced microporosity by interfacial polymerisation. *Nat. Mater.* **15**, 760–767 (2016).
